# ATAD-3 couples plasma membrane injury to mitochondrial Ca^2+^ signaling

**DOI:** 10.1038/s41467-026-77690-y

**Published:** 2026-09-10

**Authors:** Shiqi Xu, Yi Peng, Hengda Zhou, Xuecong Ren, Xin Wang, Zhenguang Ying, Meijiao Li, Yujie Sun, Bing Yang, Mao Zhang, Chonglin Yang, Weirui Ma, Suhong Xu

**Affiliations:** 1 Center for Stem Cell and Regenerative Medicine and Department of Burn and Wound Repair of the Second Affiliated Hospital, Zhejiang University-University of Edinburgh Institute, Zhejiang University School of Medicine, Hangzhou, 310058 China; 2 Department of Anatomy, Histology and Embryology, School of Basic Medical Sciences, Zhejiang University School of Medicine, Hangzhou, 310058 China; 3 School of Life Sciences, Yunnan University, Kunming, 650500 China; 4 Life Sciences Institute, Zhejiang University, Hangzhou, 310058 China; 5 Zhejiang Key Laboratory of Trauma, Burn, and Medical Rescue, Hangzhou, 310058 China; 6 Biomedical Sciences, College of Medicine and Veterinary Medicine, University of Edinburgh, Edinburgh, UK; 7 Present Address: Department of Geriatrics, Zhejiang Key Laboratory of Traditional Chinese Medicine for the Prevention and Treatment of Senile Chronic Diseases, Affiliated Hangzhou First People’s Hospital, School of Medicine, Westlake University, Hangzhou, Zhejiang China

**Keywords:** Mitochondria, Imaging, Cellular imaging

## Abstract

Mitochondrial Ca^2+^ uptake couples cellular Ca^2+^ signals to mitochondrial metabolism and stress responses and is primarily mediated by the mitochondrial Ca^2+^ uniporter (MCU). Here, we identify an ATAD-3-dependent mechanism that promotes mitochondrial Ca^2+^ uptake following plasma membrane injury in *C. elegans*. Living imaging reveals robust, albeit delayed, mitochondrial Ca^2+^ influx following injury in *mcu-1* mutants, indicating the existence of a compensatory Ca^2+^ uptake mechanism. RNAi screen identifies the conserved AAA+ ATPase ATAD-3 as an essential regulator of this pathway, whereas MICU-3 acts as a negative regulator. Wounding induces ATAD-3 oligomerization and mitochondrial enrichment, which are enhanced in *mcu-1* mutants. Disrupting ATAD-3 oligomerization or expression of disease-associated ATAD-3 variants abolishes wounding-induced mitochondrial Ca^2+^ uptake. Structure modeling predicts that oligomeric ATAD-3 adopts a channel-like architecture. These findings uncover ATAD-3 as a context-dependent regulator of mitochondrial Ca^2+^ homeostasis during cellular stress and provide insight into how this process may be disrupted in disease.

## Abstract (web summary)

In this study, Xu et al. identify ATAD-3 as a context-dependent regulator of mitochondrial Ca²⁺ uptake that is activated by cellular injury through oligomerization and mitochondrial enrichment, independent of MCU-1.

## Introduction

Calcium (Ca²⁺) signaling is a fundamental mechanism of cellular communication, regulating diverse processes ranging from energy metabolism and apoptosis to cell motility and tissue repair^1^. Among the organelles orchestrating Ca^2+^ dynamics, mitochondria play a pivotal role^2^. By buffering cytosolic Ca^2+^, mitochondria prevent toxic overload while linking Ca^2+^ signals to ATP production, thereby sustaining cellular energy demands, particularly under stress conditions^2,3^. Consistent with this essential role, dysregulation of mitochondrial Ca^2+^ dynamics is implicated in various pathological conditions, including neurodegenerative diseases, cardiovascular disorders, and metabolic syndromes^4^.

The mitochondrial Ca^2+^ uptake has been classically attributed to the mitochondrial Ca^2+^ uniporter (MCU), a highly conserved, inner mitochondrial membrane channel^4,5^. The MCU complex comprises the pore-forming subunit MCU1 together with several regulatory components, including MICU1, MICU2, MCUR1, and EMRE, which together ensure precise control of mitochondrial Ca^2+^ levels^5,6^. MICU1 and MICU2 act as Ca^2+^ sensitive gatekeepers in response to cytosolic Ca^2+^ concentrations^3,5^, whereas EMRE is essential for stabilizing the complex and enabling its function^7^. Intriguingly, genetic ablation of MCU in model organisms, such as *C. elegans*^8^, Drosophila^9^, zebrafish^10^, and mice^11^, produces no developmental defects or significant alterations in basal mitochondrial Ca^2+^ levels. These observations suggest the existence of a compensatory mechanism. Indeed, emerging evidence points to an alternative pathway for mitochondrial Ca^2+^ uptake^6,8^, yet the molecular identity and regulation of these pathways remain largely elusive.

Cellular injury represents a physiologically relevant context in which mitochondrial Ca²⁺ uptake is both rapid and functionally critical^9^. Plasma membrane damage triggers immediate cytosolic Ca²⁺ influx, which in turn activates repair pathways and reorganizes intracellular organelles^10,12^. Mitochondria rapidly respond to such Ca²⁺ surges, influencing local Ca²⁺ buffering, reactive oxygen species (ROS) production, and cytoskeletal remodeling, and metabolic adaptation required for efficient wound repair^13–17^. How mitochondria maintain Ca²⁺ uptake during injury, particularly when canonical MCU function is compromised or absent, remains an open question.

In this study, we investigate mitochondrial Ca²⁺ dynamics in vivo using the *C. elegans* epidermis as a genetically tractable and physiologically relevant model of cellular injury. We identify a robust, delayed mitochondrial Ca²⁺ uptake response that persists in the absence of MCU-1. Through an in vivo RNAi screen, we uncover ATAD-3, an inner mitochondrial membrane AAA+ ATPase, as a critical regulator of mitochondrial Ca²⁺ entry following wounding. We show that ATAD-3 rapidly accumulates at mitochondria and undergoes injury-induced oligomerization, a process that is required for mitochondrial Ca²⁺ uptake. Our findings reveal a distinct mechanism by which mitochondria adapt Ca²⁺ permeability in response to membrane injury and establish ATAD-3 as a context-dependent regulator of mitochondrial Ca²⁺ signaling in vivo.

## Results

### Wounding triggers delayed mitochondrial Ca^2+^ uptake in the absence of MCU

Epidermal wounding induces rapid cytosolic Ca²⁺ fluxes, followed by mitochondrial Ca^2+^ uptake that is classically mediated by MCU-1^18^. To examine mitochondrial Ca^2+^ dynamics in vivo, we inflicted controlled epidermal wounds in *C. elegans* using a Micropoint UV laser and monitored mitochondrial Ca^2+^ using a single-copy insertion (SCI) mitochondrial Ca^2+^ sensor (mito::GCaMP5). Upon small-scale laser wounding, wild-type (WT) animals exhibited an immediate, localized increase in mitochondrial Ca²⁺ at the wound site (Supplementary Fig. 1a and b; Supplementary Movie 1). As expected, this rapid response was abolished in *mcu-1* deletion mutants^18^(Supplementary Fig. 1a and b; Supplementary Movie 2), confirming the essential role of MCU-1 in acute mitochondrial Ca^2+^ uptake after wounding.

**Fig. 1:**
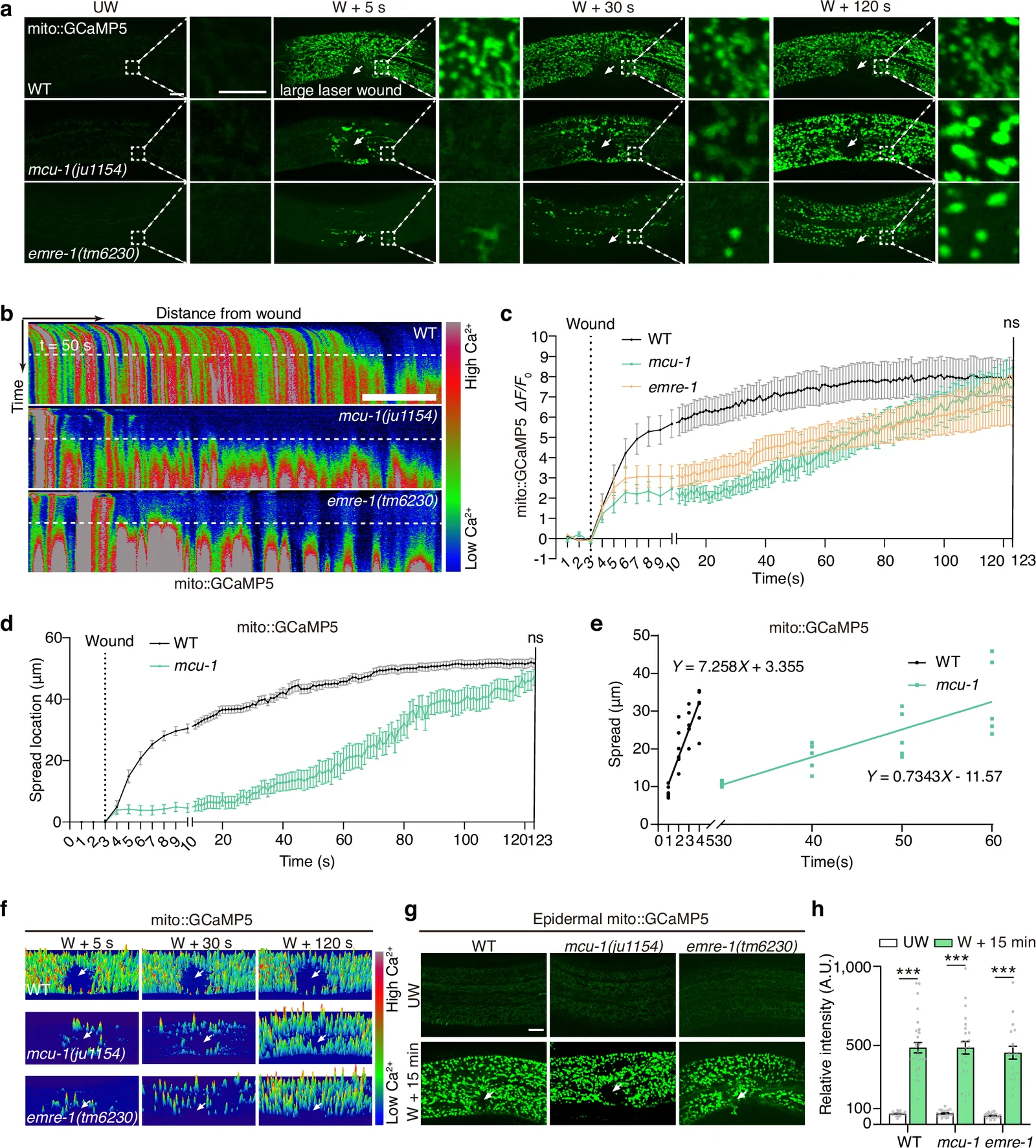
Wounding induces delayed mitochondrial Ca^2+^ uptake in the absence of MCU-1. **a** Representative confocal images of mito::GCaMP5 before and after large-scale laser wounding in wild-type (WT), *mcu-1(ju1154)*, and *emre-1(tm6230)* mutants. *Pcol-19*-mito::GCaMP5*(juSi103)* transgenic animals were used. The white arrows indicate the wound sites. Scale: 10 μm (overview) and 5 μm (zoom-in). UW: Unwounded, W: Wounded. n > 10 independent worms per genotype across at least 3 independent experiments with similar results. **b** Kymograph of mito::GCaMP5 intensity before and after large-scale laser wounding in WT, *mcu-1(ju1154)*, and *emre-1(tm6230)* mutants. Scale: 10 μm. **c** Quantification of *ΔF/F*_0_ in mito::GCaMP5 intensity throughout the continuous 2-minute period following large-scale laser wounding. WT, *n* = 10; *mcu-1*, *n* = 14; *emre-1*, *n* = 19 worms. *mcu-1, P* = 0.9562; *emre-1*, *P* = 0.7172. Two-way ANOVA with Dunnett’s multiple-comparisons test. **d** Time-lapse analysis of mito::GCaMP5 intensity change spread in WT and *mcu-1(ju1154)* mutants following large-scale laser wounding. WT, *n* = 12; *mcu-1*, *n* = 16 worms. *P* = 0.1772. Two-sided Student’s t-test. **e** Quantification of mito::GCaMP5 intensity spread speed in WT (7.258 ± 3.355 μm /s) and *mcu-1(ju1154)* mutants (0.7343 ± 11.57 μm /s) following large-scale laser wounding. WT, *n* = 6; *mcu-1*, *n* = 5 worms. **f** Surface plots of mito::GCaMP5 signal at 5, 30, and 120 seconds following large-scale laser wounding in WT, *mcu-1(ju1154)*, and *emre-1(tm6230)* mutants. The white arrows indicate the wound sites. **b**, **f** False coloring was applied in ImageJ using the Rainbow RGB LUT; the color scale bar indicates pixel intensity. **g** Confocal images of mito::GCaMP5 before and 15 minutes after needle wounding in WT, *mcu-1(ju1154)*, and *emre-1(tm6230)* mutants. The white arrows indicate the wound sites. Scale: 10 μm. **h** Quantification of mito::GCaMP5 intensity before and 15 minutes post-needle wounding. *n* = 21, 29, 22, 25, 24, 19 worms. WT, *P* < 0.0001; *mcu-1*, *P* < 0.0001; *emre-1*, *P* < 0.0001. Two-sided Student’s t-test. All data are presented as mean ± SEM. ns, *P* > 0.05. ***P < 0.001.

Strikingly, following large-scale laser wounding, WT animals displayed rapid and robust waves of mitochondrial Ca^2+^ uptake that propagated from the wound site to neighboring regions within seconds (Fig. 1a, Supplementary Movie 3). In contrast, *mcu-1* mutants showed no detectable mitochondrial Ca²⁺ uptake and spreading within the first ~ 30 seconds post-wounding (Fig. 1a and b, Supplementary Fig. 1c and d, Supplementary Movie 4). Only a few individual mitochondria exhibited increased mito::GCaMP5 signal (Fig. 1a, Supplementary Fig. 1c, Supplementary Movie 4), likely reflecting direct mitochondrial damage^18^.

Unexpectedly, after this initial latency, *mcu-1* mutants exhibited a pronounced delayed mitochondrial Ca^2+^ uptake that originated at the wound site and spread to surrounding regions, ultimately resembling the spatial pattern observed in WT animals (Fig. 1a, b, Supplementary Fig. 1c and d, Supplementary Movie 4). This delayed signal was strictly localized to the mitochondria, as confirmed by colocalization with TMRM and Tomm-20::mKate2 both before and after wounding (Supplementary Fig. 1e and f). Quantitative analysis demonstrated that the delayed increase of mito::GCaMP5 in *mcu-1* mutants ultimately reached similar levels and spread area compared to the WT (Fig. 1c–f), albeit at a slower kinetics (Fig. 1e, Supplementary Fig. 1d). This phenomenon was independently confirmed using a second mitochondrial Ca^2+^ sensor, mito::NEMOer^19^, which showed a similar delayed uptake pattern in *mcu-1* mutant (Supplementary Fig. 1g). Together, these observations suggest that wounding triggers a delayed and slower yet robust mitochondrial Ca^2+^ uptake in the absence of MCU-1, challenging the prevailing view that MCU is the sole mediator of this process.

To further explore this delayed Ca^2+^ uptake phenotype, we analyzed the mitochondrial Ca²⁺ responses in *emre-1* mutants, which lack a key regulatory subunit of MCU-1^20^. Similar to *mcu-1* mutants and previous reports, *emre-1* deletion animals failed to show immediate Ca^2+^ uptake following wounding (Fig. 1a and b, Supplementary Movie 5). Notably, *emre-1* mutants also exhibited a delayed mitochondrial Ca²⁺ uptake response (Fig. 1a–c, Supplementary Fig. 1c and d), with the Ca^2+^ signal eventually reaching a similar level to the WT and spreading from the wound site to adjacent regions (Fig. 1b and c, Supplementary Fig. 1c and d). Interestingly, unlike the *mcu-1* mutant, the *emre-1* mutant exhibited a distinct pattern: spontaneous, spike-like Ca^2+^ elevation that gradually expanded to cover a similar area (Fig. 1a and f, Supplementary Movie 5), suggesting mechanistic heterogeneity among MCU-deficient conditions.

To further validate this alternative uptake mechanism, we employed diverse wounding approaches, including needle-induced puncture and ultrasonically induced injury, in WT, *mcu-1*, and *emre-1* mutant animals. Consistent with the laser-induced wounding, we observed that 15 minutes post-wounding, mito::GCaMP5 signal in *mcu-1* and *emre-1* mutants reached levels comparable to WT animals, accompanied by fragmented mitochondrial morphology and elevated mitochondrial Ca^2+^ levels (Fig. 1g and h, Supplementary Fig. 1h). Importantly, pharmacological dissipation of Δψm with FCCP reduced, rather than enhanced, mitochondrial Ca²⁺ signal (Supplementary Fig. 1i), arguing against loss of Δψm as the primary driver of the observed Ca²⁺ uptake. Collectively, these results provide compelling evidence for a compensatory, MCU-independent mechanism that supports mitochondrial Ca^2+^ uptake following cellular injury.

### An RNAi screen identifies ATAD-3 and MICU-3 as regulators for mitochondrial Ca^2+^ uptake

**Fig. 2:**
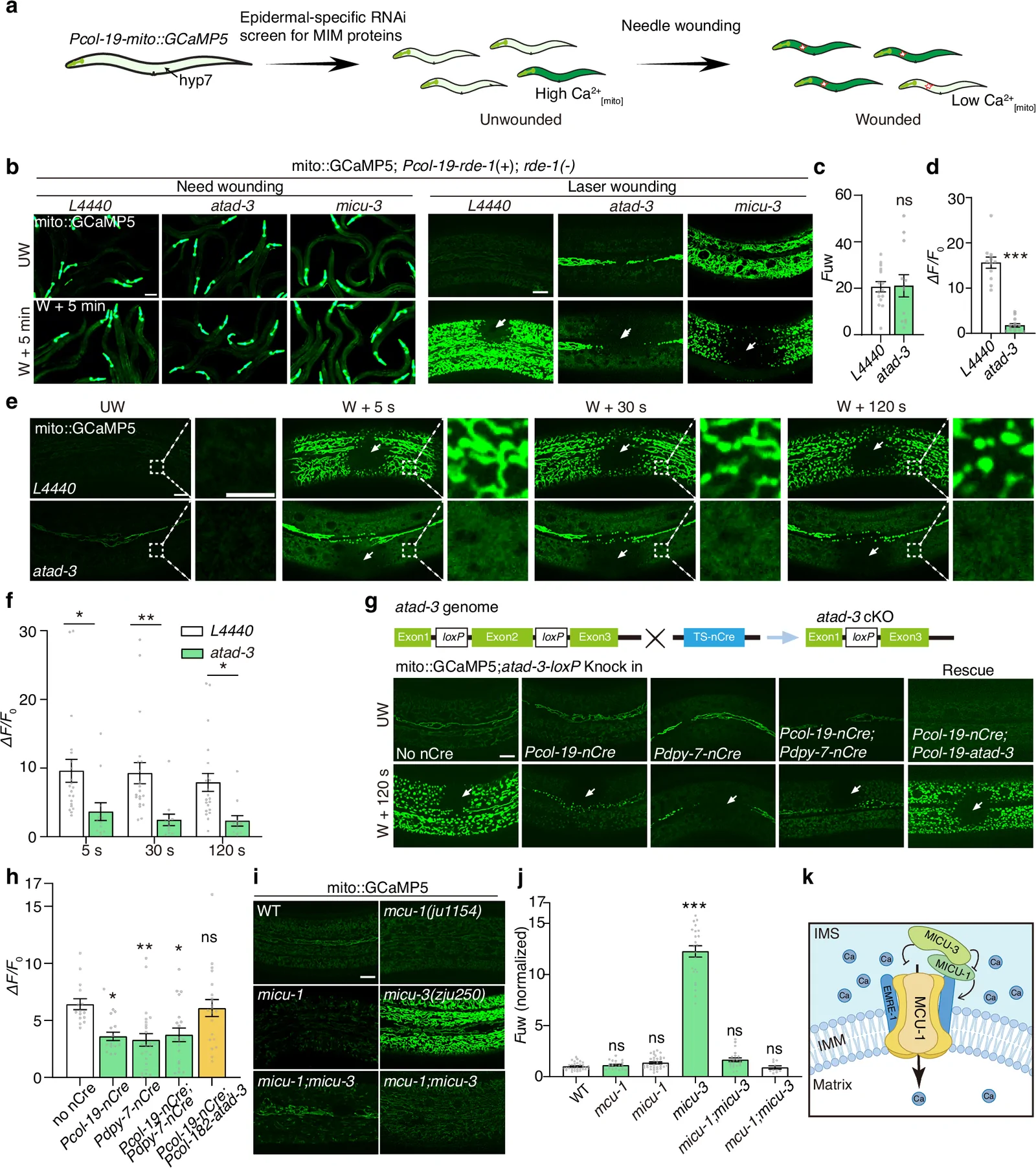
RNAi screen identifies ATAD-3 and MICU-3 as regulators of mitochondrial Ca^2+^ uptake. **a** Schematic of RNAi screen identifying regulators of mitochondrial Ca^2+^ uptake. **b** Representative confocal images of mito::GCaMP5 in *L4440, atad-3*, and *micu-3* RNAi animals before and 5 minutes after needle (left, overview) or large-scale laser (right, zoom-in) wounding. White arrows: wound sites. Scale: 100 μm and 10 μm (zoom-in). **c**, **d** Quantification of basal mito::GCaMP5 intensity (**c**) and *ΔF/F*₀ at 2 min after laser wounding (d) in *L4440* and *atad-3* RNAi animals. (**c**): *n* = 15, 12 worms. *P* = 0.9315. **d**: *n* = 12, 14 worms. *P* < 0.0001. Two-sided Student’s t-test. **e** Representative time-course confocal images of mito::GCaMP5 at indicated time points after laser wounding in control and *atad-3* RNAi animals. Scale: 10 μm and 5 μm (zoom-in). **f** Quantification of *ΔF/F*_0_ at 5, 30, and 120 seconds following laser wounding. *L4440*, *n* = 21; *atad-3*, *n* = 12. 5 s, *P* = 0.0251; 30 s, *P* = 0.0016; 120 s, *P* = 0.0026. Two-way ANOVA with Šídák’s multiple-comparisons test. **g** Top: schematic of *atad-3* conditional knockout (cKO) strategy using epidermal-specific nCre expression and CRISPR-Cas9-generated *loxP sites*. Bottom: representative confocal images of mito::GCaMP5 before and after laser wounding in WT and *atad-3* cKO animals carrying *Pcol-19*-*nCre (juSi262)*, *Pdpy-7-nCre(zjuEx3269)*, and *Pcol-19*-*nCre*;P*dpy-7*-*nCre(zjuEx3284)*. Scale: 10 μm. **h** Quantification of *ΔF/F*_0_ at 120 seconds following large-scale laser wounding in WT and *atad-3* cKO strains. *n* = 15, 14, 24, 20, 21 worms. vs no nCre: *P* = 0.0161, 0.0018, 0.0120, 0.9841. One-way ANOVA with Dunnett’s multiple-comparisons test. **i**, **j** Representative confocal images (**i**) and quantification (**j**) of basal mito::GCaMP5 signal in WT, *mcu-1(ju1154)*, *micu-1(ju1155)*, *micu-3(zju250)*, *mcu-1(ju1154)*;*micu-3(zju250)*, and *micu-1(ju1154)*;*micu-3(zju250)* strains. Scale: 10 μm. *n* = 33, 22, 44, 24, 22, 10 worms. vs WT: *P* = 0.9962, 0.6760, <0.0001, 0.1804, 0.9996. One-way ANOVA with Dunnett’s multiple-comparisons test. ** k** Schematic of the MCU complex: MCU-1 forms the pore. MICU-1 and MICU-3, and the stabilizer EMRE-1 regulate Ca²⁺ uptake. All data are presented as mean ± SEM. ns, *P* > 0.05. * *P* < 0.05, **P < 0.01, ***P < 0.001.

To identify molecular players mediating MCU-independent Ca^2+^ uptake, we conducted an in vivo RNAi screen targeting inner mitochondrial membrane (IMM) proteins (Supplementary Fig. 2a, Supplementary Table 4). Because the IMM serves as the primary gateway for Ca^2+^ entry into the mitochondria^21–23^, we hypothesized that a distinct IMM-associated regulator might facilitate Ca²⁺ uptake in the absence of the canonical MCU pathway. As many mitochondrial proteins are essential for mitochondrial integrity, cell viability, and organism development, we performed the screen in an adult epidermal-specific RNAi strain, in which *rde-1* is selectively restored in adult epidermal cells of *rde-1* mutant animals (Fig. 2a). This approach allowed us to bypass developmental lethality and specifically assess mitochondrial Ca^2+^ dynamics in adult tissues.

Following RNAi treatment, we quantified changes in mitochondrial Ca^2+^ levels using the mito::GCaMP5 reporter before and after needle-induced wounding (Fig. 2a, Supplementary Fig. 2b). Most candidate knockdowns did not affect the baseline of mito::GCaMP5 intensity. However, knockdown of *micu-3*, the *C. elegans* homolog of mammalian MICU2/3, markedly increased the baseline mito::GCaMP5 signal (Fig. 2b, Supplementary Fig. 3a), suggesting a negative regulatory role in mitochondrial Ca^2+^ homeostasis.

Strikingly, knockdown of *atad-3* had minimal effect on the basal level of mito::GCaMP5, but significantly abolished wounding-induced increases of the mito::GCaMP5 signal (Fig. 2b–d, Supplementary Movie 6). Time-lapse images further confirmed that the wound-induced increase of the mito::GCaMP5 signal was markedly suppressed in *atad-3* RNAi animals compared to controls at both early and late time points (Fig. 2e and f, Supplementary Fig. 3b). Importantly, no delayed mito::GCaMP5 signal increase was detected even 60 minutes post-wounding (Supplementary Fig. 3c and d), suggesting that mitochondrial Ca^2+^ uptake was not merely delayed but entirely blocked. Together, these results suggest that ATAD-3 is an essential regulator of wound-induced mitochondrial Ca²⁺ uptake, while MICU-3 appears to function as a negative regulator maintaining Ca²⁺ homeostasis.

To genetically validate these RNAi results, we next generated genetic deletion mutants of both *atad-3* and *micu-3*. *atad-3* encodes a conserved mitochondrial-localized AAA^+^ ATPase homologous to human ATAD3A, which has been implicated in mitochondrial ultrastructure, mtDNA stability, and Ca^2+^ homeostasis^24–26^. However, attempts to obtain homozygous *atad-3* null mutants were unsuccessful, likely due to embryonic lethality^27^. To overcome this limitation, we engineered a conditional *atad-3* allele by inserting *loxP* sites flanking two introns, enabling tissue-specific deletion via Cre recombinase (Fig. 2g). Epidermal specific expression of nuclear Cre (nCre) driven by the *dyp-7* or *col-19* promoters generated viable epidermal *atad-3* conditional knockout (cKO) animals (Fig. 2g and h, Supplementary Fig. 3e). Epidermal deletion of *atad-3* did not significantly affect mitochondrial Ca^2+^ baseline, but strongly impaired wounding-induced elevation of mito::GCaMP5 (Fig. 2g and h, Supplementary Fig. 3f). Importantly, this defect was fully rescued by epidermal expression *atad-3* cDNA (Fig. 2g and h), confirming a cell-autonomous requirement for ATAD-3 in wounding-induced mitochondrial Ca^2+^ uptake.

To examine *micu-3* function, we generated three independent *micu-3* deletion alleles using the CRISPR-Cas9-mediated editing method (Supplementary Fig. 4a). Consistent with RNAi results, all *micu-3* mutants displayed a superficial WT phenotype but exhibited significantly elevated mitochondrial Ca^2+^ levels (Supplementary Fig. 4b). In these mutants, wounding failed to elicit a further increase in mitochondrial Ca^2+^ (Supplementary Fig. 4c, Supplementary Movie 7), likely due to saturation caused by elevated baseline Ca^2+^ levels. MICU-3 shows homology to human MICU2 and MICU3. Notably, epidermal overexpression of both human *micu2* and *micu3* cDNA suppressed the elevated mito::GCaMP5 signal in *micu-3* mutant (Supplementary Fig. 4d, e), suggesting the functional conservation between *C. elegans* MICU-3 and mammalian MICU2/3 in regulating MCU-dependent mitochondrial Ca^2+^ homeostasis.

MICU proteins are known gatekeepers for mitochondrial Ca^2+^ uptake. In *C. elegans*, two MICU homologs are present: MICU-1 and MICU-3. To define their genetic relationship, we performed epistasis analysis using combined perturbations of *micu-1* and *micu-3* (Fig. 2i and j, Supplementary Fig. 4f–h). Under basal conditions, the *micu-1* mutant exhibited a mito::GCaMP5 signal comparable to WT before wounding. However, the wound-induced increase in mito::GCaMP5 fluorescence was significantly attenuated in *micu-1* mutants (Fig. 2i and j, Supplementary Fig. 4f–h), indicating that MICU-1 is required for injury-induced mitochondrial Ca^2+^ uptake. In contrast, *micu-1; micu-3* double mutants restored mito::GCaMP5 fluorescence to levels comparable to WT before wounding (Fig. 2i and j, Supplementary Fig. 4f–h), demonstrating that *micu-1* is genetically epistatic to *micu-3*. Consistently, deletion of *mcu-1* in *micu-3* mutants abolishes the elevated mitochondrial Ca^2+^ levels (Fig. 2i and j, Supplementary Fig. 4f–h). Moreover, MICU-3 physically interacts with MCU-1 in vivo (Supplementary Fig. 4i), supporting a model in which MICU-3 negatively regulates MCU-1 channel activity. Together, our genetic and biochemical data support a model in which MICU-3 acts antagonistically upstream of the MICU-1-MCU-1 axis, while MCU-1 acts as the core executioner of canonical mitochondrial Ca²⁺ entry (Fig. 2k).

Finally, to elucidate the subcellular localization of these regulators, we generated a genome insertion of GFP-tagged proteins for both MICU-3 and ATAD-3 (Supplementary Fig. 4j and k). Structured illumination microscopy (SIM) imaging revealed that both MICU-3::GFP and ATAD-3::GFP colocalized with the mitochondrial matrix marker mito::mKate2 signal (Supplementary Fig. 4j and k), supporting their positioning at the mitochondrial membrane. Notably, endogenous ATAD-3::GFP expression was low in seam cells (Supplementary Fig. 4j), potentially explaining the relatively elevated mito::GCaMP5 signals observed in seam cells upon *atad-3* RNAi knockdown or cKO animals (Fig. 2b and e). Together, these data suggest that ATAD-3 and MICU-3 act as key regulators coordinately controlling mitochondrial Ca^2+^ homeostasis and injury-induced Ca^2+^ uptake.

### ATAD-3 regulates mitochondrial Ca^2+^ uptake independently of MCU-1

**Fig. 3:**
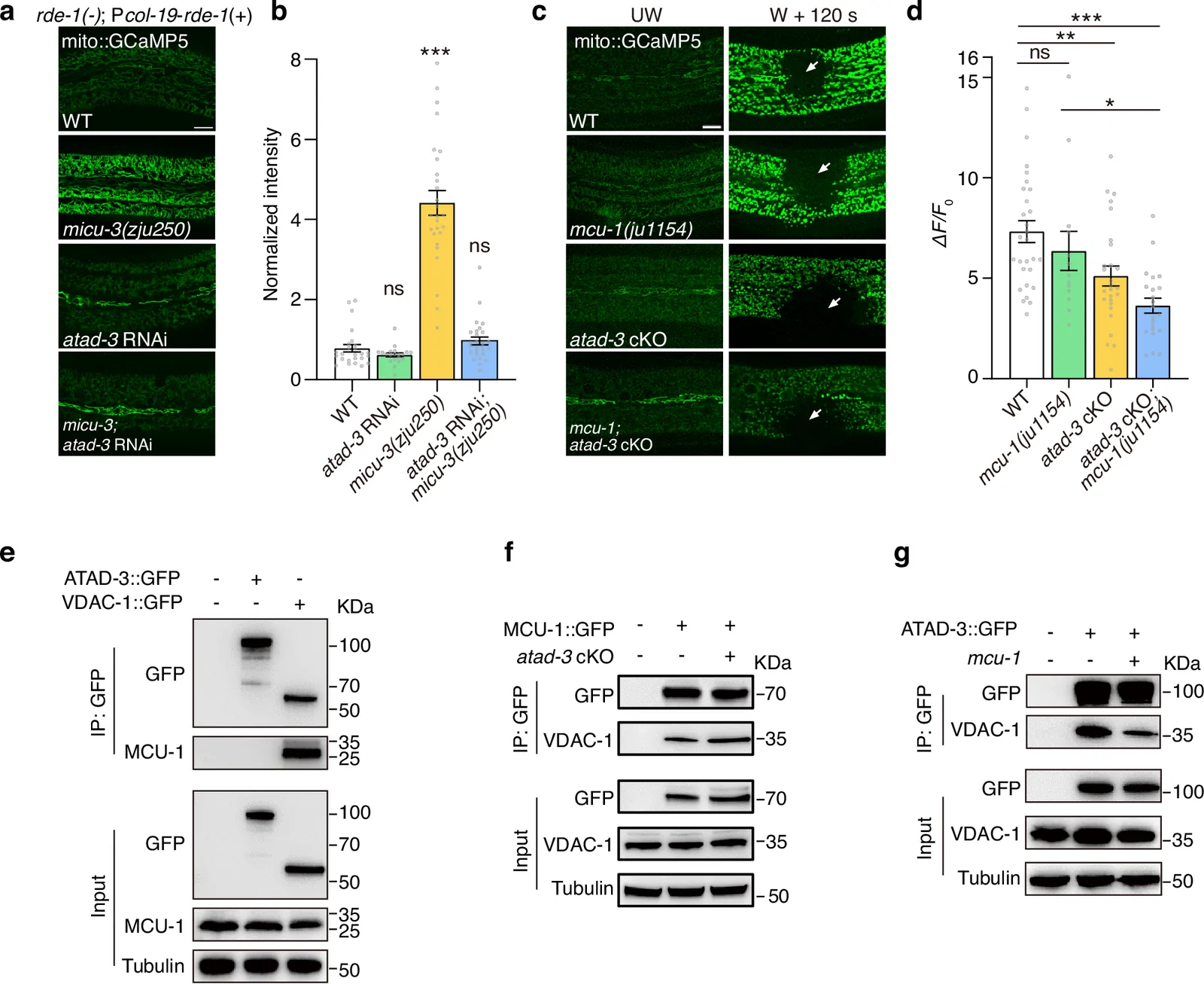
ATAD-3 regulates mitochondrial Ca^2+^ uptake independently of MCU-1. **a** Representative confocal images of mito::GCaMP5 signal in WT, *atad-3* RNAi, *micu-3(zju250)*, and *micu-3(zju250);atad-3* RNAi double mutants. Scale: 10 μm. **b** Quantification of mito::GCaMP5 intensity in WT, *atad-3* RNAi, *micu-3(zju250)*, and *micu-3(zju250);atad-3* RNAi double mutants. *n* = 25, 20, 27, 27 worms. vs WT: *P* = 0.8836, <0.0001, 0.8060. One-way ANOVA with Dunnett’s multiple-comparisons test. **c** Representative confocal images of mito::GCaMP5 signal before and after large-scale laser wounding in WT, *atad-3* cKO, *mcu-1(ju1154)*, and *mcu-1(ju1154);atad-3* double mutant. Scale: 10 μm. **d** Quantification of mito::GCaMP5 intensity 120 seconds after large-scale laser wounding in WT, *atad-3* cKO, *mcu-1(ju1154)*, and *mcu-1(ju1154);atad-3* cKO double mutants. *n* = 29, 13, 27, 22 worms. vs WT: *P* = 0.5867, 0.0076, <0.0001. One-way ANOVA with Dunnett’s correction. **e** Co-immunoprecipitation of MCU-1 with ATAD-3 and VDAC-1. *ATAD::GFP(zju283)* knock-in and *VDAC-1::GFP(zjuSi268)* SCI animals were used in this experiment. Lysates were incubated with GFP nanobody-conjugated beads, and precipitates and lysates were immunoblotted with antibodies against GFP, MCU-1, and Tubulin. *n* = 3 independent experiments with similar results. **f** Co-immunoprecipitation of VDAC-1 with MCU-1 in WT and *atad-3* cKO mutants. Lysates were incubated with GFP nanobody-conjugated beads, and precipitates and lysates were immunoblotted with antibodies against GFP, VDAC-1, and Tubulin. *n* = 3 independent experiments with similar results. ** g** Co-immunoprecipitation of VDAC-1 with ATAD-3 in WT and *mcu-1(ju1154)* mutants. ATAD::GFP *(zju283)* Knock-in animals were used in this experiment. Lysates were incubated with GFP nanobody-conjugated beads, and precipitates and lysates were immunoblotted with antibodies against GFP, VDAC-1, and Tubulin. *n* = 3 independent experiments with similar results. All data are presented as mean ± SEM. ns, *P* > 0.05. * *P* < 0.05, **P < 0.01, ***P < 0.001.

To determine whether ATAD-3 mediates mitochondrial Ca^2+^ uptake through the canonical MCU pathway, we investigated the genetic and biochemical relationships between ATAD-3, MCU-1, and associated regulators. Knockdown of *atad-3* in *micu-3* mutants completely abolished the elevated baseline mitochondrial Ca^2+^ levels in *micu-3* single mutants (Fig. 3a, b), indicating that ATAD-3 is required for MICU-3-dependent control of mitochondrial Ca^2+^ homeostasis. Similarly, *atad-3*; *mcu-1* double mutants eliminated wound-induced mitochondrial Ca^2+^ uptake, with mito::GCaMP5 signal significantly lower than in *mcu-1* single mutants (Fig. 3c, d). These results indicate that ATAD-3 is required for mitochondrial Ca^2+^ uptake following injury in both the MCU-1-dependent and -independent manner.

To further explore the relationship between ATAD-3 and MCU-1, we performed co-immunoprecipitation assays using a GFP-tagged ATAD-3 knock-in strain. Notably, we did not detect physical interaction between ATAD-3 and MCU-1 under basal conditions (Fig. 3e), suggesting that ATAD-3 does not directly interact with the MCU channel complex. In contrast, MCU-1 robustly interacted with VDAC-1 (Fig. 3f), a major outer mitochondrial membrane (OMM) channel involved in ion transport and metabolite. Epidermal-specific deletion of *atad-3* did not disrupt the MCU-1-VDAC-1 interaction (Fig. 3f), nor did it alter MCU-1::GFP expression or mitochondrial localization (Supplementary Fig. 5a and b), indicating that ATAD-3 does not regulate MCU-1 abundance, positioning, or its association with VDAC-1.

Intriguingly, ATAD-3 itself interacted with VDAC-1 (Fig. 3g), consistent with a previous report of ATAD3A in regulating Ca^2+^ flux across the OMM^26^. Moreover, deletion of *mcu-1* did not affect ATAD-3 expression or mitochondrial localization (Supplementary Fig. 5c and d), but reduced ATAD-3-VDAC-1 interaction (Fig. 3g). These observations suggest that although ATAD-3 does not directly bind to MCU-1, MCU-1 activity or Ca^2+^ flux may indirectly influence the assembly or stability of ATAD-3-VDAC-1 complex under normal conditions. Taken together, these genetic and biochemical data support a model in which ATAD-3 regulates mitochondrial Ca^2+^ uptake independently of direct MCU-1 interaction.

Because ATAD-3 has been implicated in maintaining mitochondrial morphology and structural integrity, we next asked whether defects in mitochondrial architecture might underlie the Ca²⁺ uptake phenotype. In epidermal-specific *atad-3* knockout animals, mitochondria appeared fragmented and exhibited reduced mito::GFP signal intensity (Supplementary Fig. 6a). Transmission electron microscopy (TEM) further revealed disrupted cristae organization (Supplementary Fig. 6b), consistent with previous reports^24,28^. Despite these structural abnormalities, epidermal-specific *atad-3* deletion did not alter the mitochondrial membrane potential compared to WT before and after wounding (Supplementary Fig. 6c), suggesting ATAD-3 may not regulate the mitochondrial Ca^2+^ uptake through Δψm. Consistently, *atad-3* deletion did not detectably alter mitochondrial ROS production (Supplementary Fig. 6d) or epidermal wound repair (Supplementary Fig. 6e), indicating that ATAD-3-dependent Ca²⁺ uptake is functionally distinct from the MCU-1-mtROS-wound repair axis^18^. Epidermal *atad-3* RNAi also reduced endogenous ATP-3::GFP and TOMM-20::mKate2 signals (Supplementary Fig. 6f, g), providing further evidence of compromised mitochondrial membrane integrity. Collectively, these observations raised the possibility that impaired mitochondrial Ca²⁺ uptake in *atad-3*-depleted animals might be an indirect consequence of disrupted mitochondrial architecture.

To test this hypothesis, we examined mitochondrial Ca²⁺ dynamics in *fzo-1* (mammalian homolog of MFN1/2) mutants, which exhibit pronounced mitochondrial fragmentation and reduced mitochondrial membrane potential (Supplementary Fig. 6h). Despite these defects, *fzo-1* mutants exhibited no alterations in mito::GCaMP5 signals both before and after wounding (Supplementary Fig. 6i), indicating that mitochondrial fragmentation alone is not sufficient to block mitochondrial Ca²⁺ uptake. Similarly, RNAi-mediated knockdown of *immt-1*, a core component of the mitochondrial inner membrane organizing system, did not affect mitochondrial Ca²⁺ levels under basal or wounded conditions (Supplementary Fig. 6j). Together, these results argue against a purely structural explanation and indicate that ATAD-3 regulates mitochondrial Ca²⁺ uptake through a mechanism distinct from its role in maintaining mitochondrial architecture.

### Wounding promotes ATAD-3 oligomerization and mitochondrial accumulation in vivo

**Fig. 4:**
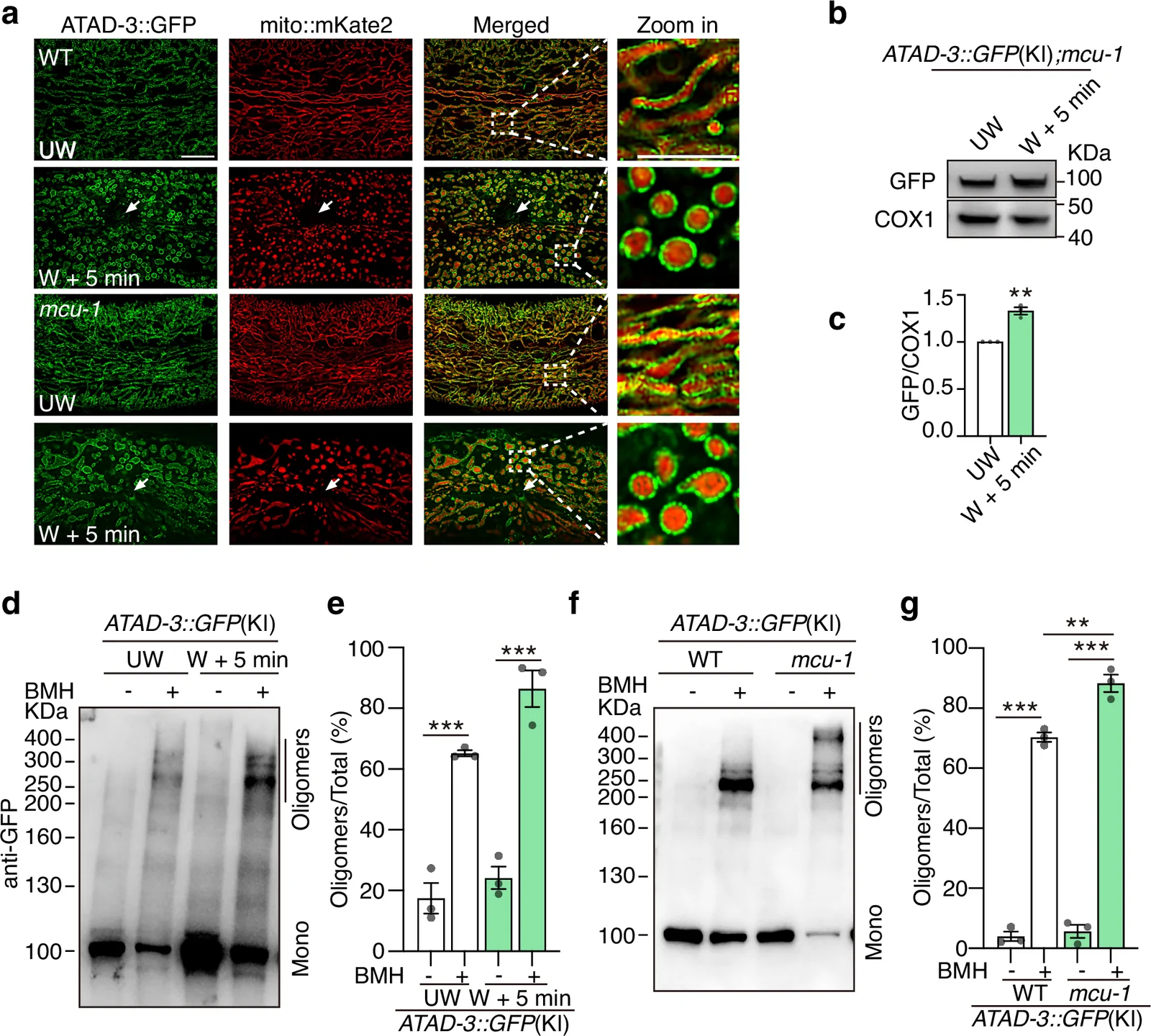
Wounding promotes ATAD-3 oligomerization and mitochondrial accumulation in vivo. **a** Representative Multi-SIM images of ATAD-3::GFP and mito::mKate2 before and after needle wounding in WT and *mcu-1(ju1154)* mutant. White arrows indicate the wound sites. Scale: 10 μm and 5 μm (zoom-in). *n* = 5 independent worms per group with similar results. **b** Western blot analysis assessing ATAD-3::GFP expression in isolated mitochondria from ATAD-3::GFP*(zju283)* knock-in; *mcu-1(ju1154)* mutants before and 5 minutes after ultrasonic wounding. *ATAD::GFP (zju283)* knock-in was used in this experiment. **c** Quantification of ATAD-3::GFP blots before and 5 minutes after ultrasonic wounding. *n* = 3 independent experiments. *P* = 0.0010. Two-sided Student’s t-test. **d** 6% SDS-PAGE analysis of ATAD-3::GFP oligomerization with BMH (100 μM) treatment before and 5 minutes after ultrasonic wounding. ATAD-3::GFP*(zju283)* knock-in strain was used for the experiment. **e** Quantification of ATAD-3::GFP oligomer ratio before and after ultrasonic wounding with BMH treatment. *n* = 3 independent experiments. UW, *P* = 0.0003; W, *P* < 0.0001. One-way ANOVA with Dunnett’s multiple-comparisons test. **f** 6% SDS-PAGE analysis of ATAD-3::GFP oligomerization in WT and *mcu-1(ju1154)* mutants with BMH (100 μM) treatment. ATAD-3::GFP*(zju283)* knock-in animals were used for the experiment. **g** Quantitation of ATAD-3::GFP oligomer ratio before and after wounding in WT and *mcu-1(ju1154)* mutants with BMH treatment. *n* = 3 independent experiments, WT, *P* < 0.0001; *mcu-1*, *P* < 0.0001. One-way ANOVA with Dunnett’s multiple-comparisons test. All data are presented as mean ± SEM. **P < 0.01, ***P < 0.001.

To elucidate how ATAD-3 regulates mitochondrial Ca²⁺ uptake, we examined its subcellular dynamics in response to cellular injury. Using an endogenous ATAD-3::GFP knock-in strain in combination with mitochondrial marker mito::mKate2, we monitored ATAD-3 localization and dynamics following wounding. Under basal conditions, ATAD-3::GFP appeared as discrete puncta with limited mitochondrial enrichment (Fig. 4a). Strikingly, within 5 minutes after wounding, ATAD-3::GFP became markedly enriched on mitochondria accumulated around mitochondria (Fig. 4a, Supplementary Fig. 7a), suggesting a rapid wound-induced redistribution or accumulation. This mitochondrial enrichment was also observed in *mcu-1* mutants (Fig. 4a), indicating a rapid and robust injury-induced mitochondrial redistribution.

Consistent with these imaging observations, mitochondrial fractionation revealed a significantly wounding-dependent enrichment of ATAD-3::GFP specifically in the mitochondrial fraction, with the response further enhanced in *mcu-1* mutants (Fig. 4b and c). Immunoblot analysis of whole-worm lysates also showed an apparent increase in ATAD-3::GFP signals at 5 minutes post-wounding (Supplementary Fig. 7b); however, this increase might not reflect de novo protein synthesis on this timescale, but rather the injury-induced changes in ATAD-3 biochemistry, including oligomerization and tighter inner membrane integration, that improve protein extractability and immunodetection efficiency from whole-animal preparations. Together, these data indicate that wounding triggers a rapid and transient accumulation of ATAD-3 at mitochondria, a response that is enhanced when MCU-1-dependent Ca^2+^ entry is compromised.

Given previous reports that human ATAD3A forms oligomers under stress and disease conditions^29,30^, we next investigated whether ATAD-3 undergoes injury-induced oligomerization in vivo. Using BMH crosslinking assays, we detected robust ATAD-3::GFP oligomer formation under basal conditions, with higher-order species present even in the absence of injury (Fig. 4d and e, Supplementary Fig. 7c). Notably, wounding further enhanced ATAD-3::GFP oligomerization, leading to a marked increase in higher-order oligomeric complex (Fig. 4d and e). This effect was significantly stronger in *mcu-1* mutants than in WT animals (Fig. 4f and g), and further amplified by wounding (Supplementary Fig. 7d). Together, these results indicate that ATAD-3 oligomerization is a key injury-induced response and further potentiated in the absence of MCU-1, linking ATAD-3 assembly to its role in mitochondrial Ca^2+^ handling.

### ATAD-3 oligomerization is required for mitochondrial Ca^2+^ uptake

**Fig. 5:**
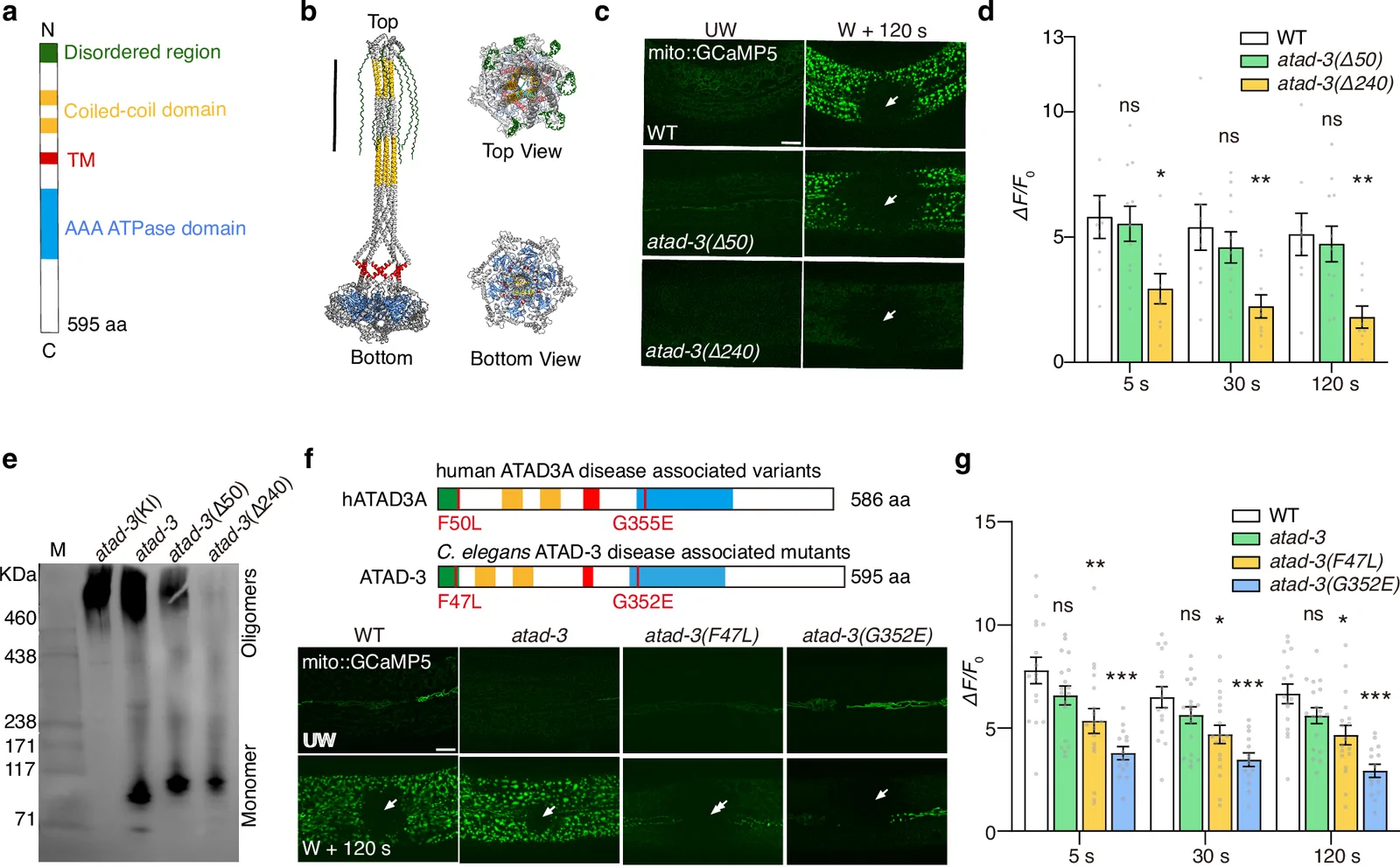
ATAD-3 oligomerization is required for mitochondrial Ca^2+^ uptake. **a** Schematic of ATAD-3 protein domain structure. **b** Structural views of the AlphaFold3-predicted hexameric ring-like conformation of ATAD-3. Left: side view. Right: top view (upper) and bottom view (lower). Scale bar: 10 nm. **c** Representative confocal images of mito::GCaMP5 signal before and after large-scale laser wounding in WT and ATAD-3(Δ50, Δ240) overexpression strains. *Pcol-182-atad-3(50-595) cDNA(zjuEx3154)*, and *Pcol-182-atad-3(240-595) cDNA(zjuEx3153)* transgenic animals were used to overexpress *atad-3* specifically in the epidermal cell. White arrows indicate the wound sites. Scale: 10 μm. **d** Quantification of Δ*F/F*_0_ in mito::GCaMP5 intensity change at 5, 30, and 120 seconds before and after large-scale laser wounding in WT and ATAD-3(Δ50, Δ240) overexpression strains. WT, *n* = 9; *atad-3(∆50)*, *n* = 11; *atad-3(∆240), n* = 9 worms. vs WT: 5 s, *P* = 0.9566, 0.0289; 30 s, *P* = 0.6919, 0.0173; 120 s, *P* = 0.9146, 0.0085. Two-way ANOVA with Dunnett’s correction. **e** Native PAGE analysis of ATAD-3::GFP in ATAD-3::GFP (knock-in), ATAD-3::GFP, ATAD-3(Δ50)::GFP, and ATAD-3(Δ240)::GFP overexpression strains. *n* = 2 independent experiments with similar results. **f** Top: schematic of ATAD-3 protein conservation with human ATAD3A and disease-associated mutations. Bottom: representative confocal images of mito::GCaMP5 signal before and after large-scale laser wounding in WT and *ATAD-3(F47L, G352E)* overexpression strains. *Pcol-182-atad-3 cDNA(zjuEx3140), Pcol-182-atad-3(F47L) cDNA(zjuEx3126)*, and *Pcol-182-atad-3(G352E) cDNA(zjuEx3156)* transgenic animals were used to overexpress *atad-3* specifically in the epidermal cell. White arrows indicate the wound sites. Scale: 10 μm. **g** Quantification of Δ*F/F*_0_ in mito::GCaMP5 intensity change at 5, 30, and 120 seconds post-high-power laser wounding in WT and ATAD-3(F47L, G352E) overexpression strains. *Pcol-182-atad-3(F47L)(zjuEX3126)* and *Pcol-182-atad-3(G352E)(zjuEx3156)* transgenic animals were used to overexpress *atad-3* specifically in the epidermal cell. *n* = 16, 18, 18, 14 worms. *P* = 0.1464, <0.0001, <0.0001, <0.0001. Two-way ANOVA with Dunnett’s multiple-comparisons test. All data are presented as mean ± SEM. ns, *P* > 0.05. * *P* < 0.05, **P < 0.01, ***P < 0.001.

AAA^+^ ATPases typically function as oligomeric assemblies, often assembling into hexameric ring-like structures that mediate substrate transport or remodeling^31,32^. ATAD-3 protein is highly conserved between *C. elegans* and humans, and shares canonical structural features, including an N-terminal intrinsically disordered region (IDR), a coiled-coil domain, a transmembrane segment, and a C-terminal AAA^+^ ATPase domain (Fig. 5a). To gain insight into the structural basis of ATAD-3 function, we performed predictive structure modeling using AlphaFold3^33,34^. The predicted structure revealed that ATAD-3 assembles into a hexameric, ring-like structure reminiscent of other AAA^+^ ATPases^32^ (Fig. 5b). In this configuration, the transmembrane domain anchors ATAD-3 to the IMM, while the N-terminal coiled-coil domain extends into the intermembrane space, forming a long channel-like architecture (Fig. 5b). The oligomerized C-terminal AAA^+^ ATPase domain generates a central pore with a predicted diameter ranging from 6.7 to 19.4 Å (Fig. 5b), a size compatible with Ca^2+^ permeation.

To investigate whether ATAD-3 oligomerization is required for mitochondrial Ca^2+^ uptake, we generated two N-terminal truncation mutations of ATAD-3(Δ50), which removes the IDR, and ATAD-3(Δ240), which deletes the coiled-coil domain critical for oligomerization while preserving mitochondrial localization (Supplementary Fig. 8a and b). Overexpression of ATAD-3(Δ240) nearly abolished wound-induced increase of mitochondrial Ca^2+^ uptake and resulted in abnormal mitochondria (Fig. 5c and d, Supplementary Fig. 8c and d). In contrast, overexpression of ATAD-3(Δ50) had no detectable effect on mitochondrial Ca^2+^ dynamics (Figs. 5c and 5d, Supplementary Fig. 8c and d). Consistent with these functional results, native PAGE gel assays revealed that ATAD-3(Δ240)::GFP failed to form oligomers, whereas ATAD-3(Δ50)::GFP retained its oligomerization capacity (Fig. 5e).

To further examine the injury-induced redistribution of ATAD-3, we performed HiS-SIM imaging in WT and mutant ATAD-3 before and after wounding (Supplementary Fig. 8e). Quantification of mitochondrial ATAD-3::GFP/mito::mKate2 fluorescence ratio revealed a significant increase in mitochondrial ATAD-3 enrichment, whereas no apparent increase was detected in ATAD-3(Δ240)::GFP animals after wounding (Supplementary Fig. 8f), suggesting that deletion of the coiled-coil domain significantly attenuates the injury-induced enrichment and redistribution of ATAD-3 on mitochondria. Structural modeling further confirmed that deletion of the coiled-coil domain disrupts the channel-like architecture of the hexameric assembly, whereas removal of the IDR alone does not (Supplementary Fig. 8a). Together, these findings suggest that the ATAD-3 oligomerization, mediated by its coiled-coil domain, is required for wounding-induced mitochondrial Ca^2+^ uptake.

Mutations in human ATAD3A are associated with neurodegenerative diseases and cardiomyopathy, although their effects on mitochondrial Ca²⁺ handling remain poorly understood^25,35^. To assess the functional consequence of disease-associated variants, we generated *C. elegans* strains expressing pathogenic human ATAD3A mutants (Fig. 5f, Supplementary Fig. 8g, h). Overexpression of two such mutants, F47L and G352E, did not affect baseline mitochondrial Ca²⁺ levels but significantly inhibited wound-induced mitochondrial Ca²⁺ uptake (Fig. 5f and g, Supplementary Fig. 8h, i). Both mutant proteins localized normally to the mitochondria (Supplementary Fig. 8b), suggesting that they act as dominant-negative forms rather than through mislocalization. Notably, F47 resides within the N-terminal IDR, while G352 is located in the conserved AAA^+^ ATPase domain (Fig. 5f, Supplementary Fig. 8j), highlighting the importance of both domains for ATAD-3 assembly and function. Together, these findings indicate that disease-associated ATAD-3 mutations impair stress-induced mitochondrial Ca²⁺ uptake, providing a potential mechanistic link between ATAD3A dysfunction, defective mitochondrial Ca^2+^ signaling, and human disease.

## Discussion

**Fig. 6:**
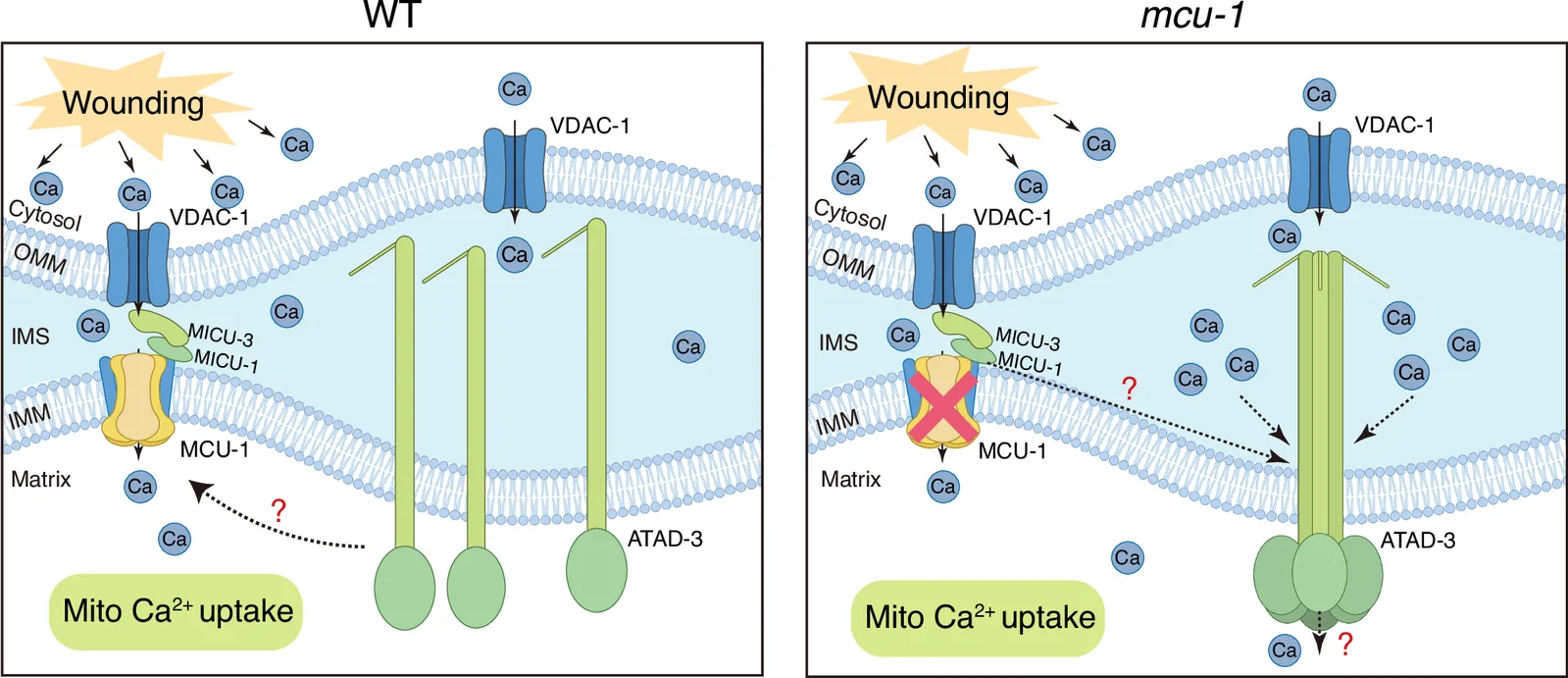
Model for ATAD-3-mediated regulation of mitochondrial Ca^2+^ uptake during plasma membrane injury. In wild-type epidermal cells, plasma membrane injury triggers a rapid increase in cytosolic Ca²⁺, which is subsequently taken up into the mitochondrial matrix through the MCU-1 uniporter complex. Within this complex, MCU-1 forms the Ca^2+^-conducting pore in the inner mitochondrial membrane (IMM), while MICU-1 and MICU-3 modulate channel activity from the intermembrane space (IMS) side. ATAD-3 is additionally required for efficient mitochondrial Ca²⁺ uptake. In *mcu-1* mutants, the canonical MCU-1-dependent pathway is disrupted. Nevertheless, cellular injury still induces a delayed mitochondrial Ca²⁺ response through an MCU-1-independent but ATAD-3-dependent pathway, potentially associated with injury-induced ATAD-3 oligomerization and mitochondrial accumulation. Subcellular compartments, including outer mitochondrial membrane (OMM), intermembrane space (IMS), inner mitochondrial membrane (IMM), and matrix, are indicated. Arrows denote the direction of Ca²⁺ flux and the proposed sequence of regulatory events in the MCU-1-dependent and ATAD-3-dependent uptake pathways.

Based on our genetic, cell biological, and functional analyses, we identify ATAD-3 as a critical regulator of injury-induced mitochondrial Ca²⁺ uptake. Under physiological conditions, ATAD-3 is required for efficient MCU-1-mediated Ca²⁺ entry following plasma membrane damage, acting permissively for MCU function without directly interacting with MCU-1 (Fig. 6). In the absence of MCU-1, ATAD-3 additionally enables an MCU-independent Ca²⁺ uptake pathway that is activated upon injury. We propose that this compensatory activity may involve injury-induced ATAD-3 oligomerization within the IMM and enable Ca²⁺ influx independently of MCU (Fig. 6).

In parallel, we characterize MICU-3 as a negative regulator of mitochondrial Ca^2+^ uptake, functioning upstream of MCU and modulating basal Ca^2+^ dynamics. Genetic epistasis analyses reveal that MICU-3 antagonizes MICU-1 and restricts MCU activity, thereby shaping both the threshold and amplitude of mitochondrial Ca²⁺ entry. The opposing activities of MICU-1 and MICU-3, together with the dual role of ATAD-3, underscore the layered and adaptable nature of mitochondrial Ca²⁺ regulation. This regulatory architecture likely ensures that mitochondria can dynamically tune Ca²⁺ uptake across a wide range of physiological and stress conditions, preventing both Ca²⁺ overload and insufficient buffering during acute injury.

The demonstration that mitochondria retain the capacity for Ca²⁺ uptake in the absence of MCU-1 has profound implications for mitochondrial physiology. While the MCU complex has long been regarded as the primary pathway for Ca²⁺ entry into mitochondria^36,37^, our findings reveal a robust, ATAD-3-dependent pathway that becomes engaged under stress. This pathway enables mitochondria to maintain Ca^2+^ buffering and signaling during cellular wounding, thereby supporting mitochondrial function and cellular resilience. Our work complements previous reports suggesting an alternative Ca^2+^ uptake mechanism^8,23^, but crucially extends these observations by identifying a specific molecular regulator in vivo.

The absence of detectable ROS or wound repair defects in *atad-3*-depleted animals, despite reduced wound-induced Ca^2+^ uptake, is best understood by contrasting the nature of the Ca^2+^ deficit in *atad-3* versus *mcu-1* loss-of-function contexts. In *mcu-1* mutants, acute Ca^2+^ signaling is specifically abolished while mitochondrial structure remains intact, and this alone is sufficient to impair ROS production and wound repair. By contrast, *atad-3* epidermal-specific depletion causes chronic structural mitochondrial dysfunction, while MCU-1 remains intact. Residual Ca^2+^ entry likely sustains the threshold matrix Ca^2+^ concentration required for mitochondrial ROS production. Additionally, ATAD-3-mediated and MCU-1-mediated Ca^2+^ entry may engage distinct downstream effector programs, consistent with their different submitochondrial locations and structural, rather than bioenergetic, phenotypes associated with *atad-3* loss. Together, these observations support a model in which distinct mitochondrial Ca^2+^ uptake pathways are functionally non-interchangeable and couple cellular injury to different mitochondrial outputs depending on the molecular identity and context of the uptake route.

The capacity of ATAD-3 to functionally substitute for MCU-1 highlights its potential therapeutic target for conditions involving mitochondrial Ca²⁺ dysregulation. ATAD-3 has been previously implicated in mitochondrial membrane organization, cristae maintenance, and mtDNA stability^24,25,29,30^, yet its involvement in Ca²⁺ regulation had not been appreciated. We demonstrate that wounding induces rapid ATAD-3 oligomerization and transient mitochondrial accumulation, processes that are essential for its function in Ca²⁺ uptake. Notably, oligomerization is further enhanced in *mcu-1* mutants, suggesting that ATAD-3 acts as a compensatory mechanism in the absence of MCU-1. These findings raise important mechanistic questions regarding how ATAD-3 oligomers facilitate Ca²⁺ entry. ATAD-3 may directly participate in Ca^2+^ transport, regulate the activity or assembly of other mitochondrial Ca^2+^ transport machinery, or remodel the local membrane properties to favor Ca²⁺ flux. Resolving these possibilities will require high-resolution structural studies, reconstitution of ATAD-3 assemblies, and electrophysiological recording in defined biochemical systems.

In conclusion, our work establishes ATAD-3 as a critical regulator of mitochondrial Ca²⁺ uptake under stress, revealing a MCU-independent pathway that preserves mitochondrial function and cellular survival during tissue injury. By defining ATAD-3 as both a structural and functional regulator of stress-induced Ca²⁺ uptake, this study expands the molecular framework of mitochondrial Ca²⁺ regulation and highlights the adaptive capacity of mitochondria in response to membrane damage. Given the association of ATAD3A mutations with neurodegenerative and metabolic diseases, elucidating the broader physiological roles and regulatory mechanisms of ATAD-3-mediated Ca²⁺ uptake may provide insights into mitochondrial dysfunction and identify potential strategies to enhance mitochondrial resilience in disease and tissue injury.

## Methods

### Strains

*C. elegans* strains were cultured on the nematode growth medium (NGM) plates seeded with *Escherichia coli* OP50 following standard protocols at 20-22.5 °C^38^, unless otherwise indicated. Unless indicated otherwise, all experiments in this study were performed using adult hermaphrodites. The N2 Bristol strain was used as the wild-type (WT) strain. Standard microinjection methods were used to generate transgenic animals carrying extrachromosomal arrays (*zjuEx*). Single-copy insertion of arrays (*zjuSi*) and GFP fusion knock-in strains (*zju*) were generated using CRISPR-Cas9-mediated genome editing methods, as described in the following sections. All strains used in this work are listed in Supplementary Table 1.

### Constructs and transgenic animals

Transgenic expression plasmids were generated using a seamless cloning kit (CloneExpress II One Step Cloning Kit, Vazyme, Cat. No. C112) and LR recombination (LR Clonase II Enzyme Mix, Thermo Fisher Scientific, Cat. No. 11791020). For seamless cloning, target fragments were PCR-amplified with primers bearing 15-20 bp homologous sequences matching the ends of the linearized destination vector, allowing direct recombination. LR recombination was used to transfer entry clones into destination vectors carrying tissue-specific promoters or to assemble donor insertion plasmids for single-copy insertion. All constructs were confirmed by Sanger sequencing.

Extrachromosomal array transgenes were generated by microinjecting gene constructs at concentrations of 5 or 10 ng/μl, along with a co-injection marker (P*ttx-3*-RFP at 50 ng/μl or P*myo-3*-RFP at 5 ng/μl). The single-copy insertion of *zjuSi345* (*Pcol-19-*MICU-3::GFP*-myc*) was created using a Cas9-based insertion method as previously reported^39^, with an sgRNA targeting the *ttTi4348* locus on chromosome I and donor insertion plasmids. Correct insertions were identified by PCR screening and confirmed by DNA sequencing. All newly generated constructs and strains are listed in Supplementary Tables 1 and 2 and are available from the corresponding author upon reasonable request.

### CRISPR-Cas9-Mediated Gene Knock-In

The ATAD-3*::*GFP knock-in *(zju283)* animals were generated using the CRISPR-Cas9 system^39^. The donor repair template includes 1.5 kb of the *atad-3* 5′ untranslated region (UTR), the GFP coding sequence (including ATG), and an additional 1.5 kb of *atad-3* genomic sequence downstream of the start codon. Injection mixtures containing the repair plasmids pSX3028 (ATAD-3::GFP knock-in repair template) at 55 ng/μl, pSX3187 and pSX3188 as pU6-sgRNAs (each at 35 ng/μl), and pSX524 (P*eft-3*-cas9-NLS-Pu6-*dpy-10* sgRNA) at 45 ng/μl as a co-injection red marker were injected into N2 worms. Injected animals were cultured at 25 °C for 2–3 days, and F1 animals displaying dumpy/roller or red fluorescent phenotype were isolated and transferred to a new fresh plate. F2 progeny exhibiting GFP fluorescence without co-injection markers were selected, confirmed by PCR and DNA sequencing, and outcrossed with N2 to remove *dpy-10* mutations. All knock-in strains displayed normal morphology and development. All sgRNA sequences are listed in Supplementary Table 3.

### CRISPR-Cas9-Mediated Mutagenesis

*micu-3* (C56A3.6*)* mutant alleles were generated using CRISPR-Cas9 genome editing system^40^. A mixture of plasmids encoding sgRNAs targeting *micu-3* (pSX2572 and pSX2573 at 30 ng/µl) was co-injected with pSX524 (P*eft-3*-cas9-NLS- pU 6-*dpy-10* sgRNA, 60 ng/µl). Following incubation at 25 °C for 3 days, F1 animals exhibiting dumpy/roller phenotypes were isolated and transferred to fresh plates. Homozygous mutants were identified by PCR and DNA sequencing. All sgRNA sequences are listed in Supplementary Table 3.

### Adult epidermal-specific RNAi

Adult epidermal-specific RNAi was performed using the *rde-1(ne219); Pcol-19-rde-1(juIs346)* strain as previously described^41,42^. The approach restricts RNAi activity to the epidermis by expressing the Argonaute protein RDE-1 under the control of the epidermal-specific promoter *col-19* in an otherwise RNAi-deficient genetic background (*rde-1(ne219)*). Briefly, synchronized L1 stage worms carrying the *rde-1(ne219); Pcol-19-rde-1(juIs346)* genotype were placed on NGM plates seeded with *E. coli* HT115 expressing dsRNA, supplemented with 1 mM Isopropyl β-D-1-thiogalactopyranoside (IPTG) and carbenicillin. Animals were grown at 20 °C until they reached the young adult stage for subsequent experiments. All RNAi candidates are listed in Supplementary Table 4.

### Wounding procedures

Wounding of the animals was performed using Micropoint UV laser, microinjection needle puncture, or ultrasonic sonication as described previously^43^. Young adult worms (synchronized 24 h post-L4 at 20 °C) were utilized for wounding experiments. Briefly, laser wounding was performed with a pulsed UV laser on agarose-mounted worms immobilized with levamisole (12 μM), targeting comparable epidermal regions. For mechanical injury, young adult worms were punctured 1-2 times in anterior/posterior regions using a glass microinjection needle under a dissection microscope, and transferred to agar pads for imaging. For ultrasonic wounding, worms suspended in M9 buffer were subjected to four cycles of sonication (0.1 s each at 10% power) using a JY92-IIDN ultrasonic processor (ScienPrior).

Small-scale laser wounding was performed at ~ 60% laser power(355 nm pulsed UV, Micropoint, Andor Technology), producing a wound diameter of ~1 μm. This approach generates highly localized plasma membrane disruption with minimal collateral tissue damage, and is used for experiments requiring precise spatial and temporal control of the injury stimulus, including live imaging of local mitochondrial Ca²⁺ dynamics and mitochondrial responses immediately following injury.

Large-scale laser wounding was performed at ~80% laser power, producing a wound diameter of ~10 μm. This approach generates a broader cellular injury more comparable in scale to needle wounding in *C. elegans* epidermis, and is used for experiments that require a more robust injury stimulus, including mitochondrial Ca^2+^ uptake wave propagation and other wound responses. We note that because laser power output varies across instruments, focal plane, and degrades over time with UV laser aging, wound diameter rather than laser power percentage should serve as the primary reproducibility parameter when adapting these protocols to other systems.

Needle wounding was selected for the large-scale RNAi screen because it is operationally straightforward, highly reproducible across large cohorts, and requires no specialized equipment except a dissection microscope, properties essential for high-throughput applications in which dozens of gene knockdowns must be evaluated in parallel. The larger wound area also generates a more robust injury response, increasing sensitivity for detecting partial loss-of-function phenotypes under incomplete RNAi knockdown conditions. Laser wounding was reserved for the mechanistic and quantitative imaging experiments in Figs. 2–5, where controlled wound geometry and precise temporal resolution of the injury stimulus are essential.

Ultrasonic wounding produces widespread yet consistent mechanical disruption across the sample. This approach generates mechanical injury without direct plasma membrane ablation, and is used for experiments requiring high-throughput processing and validation of phenomena independent of laser-specific effects, including population-level Western blot analysis and verifying the generality of mitochondrial Ca²⁺ uptake following injury.

### Fluorescence imaging

Animals were immobilized on 4% agarose pads in 12 mM levamisole and imaged using a spinning-disk confocal microscope (Andor, 100 x, NA 1.46 objective; with IQ CORE imaging software, Nikon) before and after wounding. GFP was excited with a 488 nm laser, and RFP with a 561 nm laser. Super-resolution imaging was performed using either Multimodality Structured Illumination Microscopy (Multi-SIM, NanoInsights-Tech, Guangzhou, China) or High Intelligent and Sensitive SIM (HiS-SIM, Guangzhou Chaoshiji Biotechnology, Guangzhou, China). Images were captured using a 100 × /1.5 NA oil immersion objective (Olympus) under the control of the manufacturer’s software. Raw SIM datasets were reconstructed using a Wiener filter-based algorithm for defocus elimination, artifact removal, and image denoising.

### Image analysis and quantification

Image processing and quantitative analyses were performed using ImageJ (NIH). For visualization only, a Rainbow RGB lookup table (LUT) was applied to the images shown in Fig. 1b and f, Supplementary Fig. 3b and 3 f. Because the Rainbow LUT is a non-linear mapping of pixel intensity to display color, it was used solely for qualitative presentation, and no quantitative intensity measurements were obtained from the pseudo-colored image. For the Ca^2+^ signal, baseline Ca^2+^ (*F*uw) and wound-induced Ca^2+^ levels (*F*w) were quantified by measuring GCaMP5 fluorescence intensity within regions of interest (ROIs) encompassing epidermal cell areas while excluding the wound cavity and background. Background subtraction was performed using adjacent tissue-free regions. Ca^2+^ responses were expressed as normalized Δ*F/F*_0_ values calculated using the formula: Δ*F/F*_0_ = (*F*w-*F*uw)/*F*uw as previously described^41^.

For spatial propagation analyses, vertical intensity profiles were generated by averaging fluorescence intensities along lines orthogonal to the animal's longitudinal axis. The propagation threshold was defined as the midpoint between the maximum and minimum fluorescence values, and propagation distance was quantified as the furthest position exceeding this threshold. For linear propagation analysis, optimal time windows were selected based on initial velocity measurements (1-4 seconds post-wounding for WT vs. 30-60 seconds for *mcu-1* mutants). Temporal resolution of signal propagation was analyzed by generating kymographs using the *Kymograph* plugin (maximum intensity projection mode). Three-dimensional surface plots were generated using the ImageJ *3D Surface Plot* plugin.

For quantification of mitochondrial fragmentation, mito::GFP signals were analyzed using the ImageJ plugin *Mitochondrial Analyzer*. After automatic thresholding, mitochondrial morphology parameters, including mean branch length, were quantified.

For quantification of injury-induced mitochondrial enrichment of ATAD-3, individual mitochondrial regions of interest (ROIs) were generated from the mito::mKate2 channel using ImageJ. A threshold was applied to segment mitochondria, followed by morphological dilation to encompass the entire mitochondrial structure. The mean fluorescence intensities of ATAD-3::GFP and mito::mKate2 were measured, and mitochondrial ATAD-3 enrichment was calculated as the ratio of GFP to mKate2 fluorescence (ATAD-3::GFP/mito::mKate2). To quantify injury-induced redistribution of ATAD-3, the enrichment ratio was measured before and 5 min after wounding. Comparisons were performed within each genotype to minimize variation arising from differences in transgene expression levels.

### Protein crosslinking

For in vivo ATAD-3 crosslinking, approximately 1000 animals were collected, washed three times with 1 ml M9 buffer, and lysed with 100 µl RIPA buffer (#9806S, Cell Signaling Technology, USA) at 4 °C for 30 minutes. Lysates were centrifuged at 14,000 x *g*, and supernatants were collected and incubated with 100 μM bismaleimidohexane (BMH, CAS: 4856-87-5, Macklin, Shanghai) at room temperature (RT) for 30 minutes. Reactions were quenched and analyzed by SDS-PAGE followed by immunoblotting.

### Mitochondrial isolation

Mitochondria were isolated from synchronized young adult *C. elegans* using the High-Purity Mitochondria Extraction Kit (EpiZyme, Cat. No. PC205) according to the manufacturer’s protocol, with all steps performed on ice at 4 °C. Briefly, worms with a packed volume of approximately 1 mL (~10,000 animals) were resuspended in extraction buffer A and mechanically homogenized. The homogenate was subjected to an initial low-speed centrifugation at 700 × *g* to remove cellular debris and unlysed worms, and the resulting supernatant was centrifuged at 10,000 × g to pellet crude mitochondria. Crude mitochondria were further purified by density gradient centrifugation over a C/D buffer mixture (17:3, v/v) at 21,000 × *g*. Mitochondrial purity was verified by Janus Green B staining of the final pellet. Purified mitochondria were then lysed in lysis buffer, and the resulting extract was used directly for Western blot analysis.

### Immunoprecipitation and Immunoblotting

For in vivo co-immunoprecipitation (Co-IP), approximately 10,000 animals were collected and washed three times with 10 ml M9 buffer and lysed with RIPA buffer. The supernatants were incubated with the GFP nanobody-conjugated beads (30 μl, KT Life technology, KTSM1301, China) for 1 hour at 4 °C. Immunoprecipited samples were then blotted with primary antibodies, including anti-GFP (1:2500, MBL598, Japan), anti-MCU (1:1000, Proteinteck, 26312-1-AP, USA), anti-COX4 (1:2000, Proteinteck, 66110-1-Ig, USA), anti-VDAC-1 (1:2000, Proteinteck, 10866-1-AP, USA) or anti-Tubulin (1:2000, Huabio, EM0103, China), overnight at 4 °C and then incubated with the secondary antibodies 1 hour at RT. The samples were detected using the ChemiDoc Touch Imaging System (Bio-Rad, 1708370, USA).

### Blue-Native (BN)-PAGE and 2D SDS-PAGE

Worms were lysed in 1× NativePAGE sample buffer containing 1% DDM (Thermo Fisher, BN2008, USA). Lysates were separated on 3-12% Bis-Tris NativePAGE gels (Thermofisher, BN1001BOX, USA) and transferred to a nitrocellulose membrane using 1 × NuPAGE transfer buffer (Thermofisher, NP0006, USA). Membranes were blocked with 5% BSA and probed with anti-GFP antibodies.

### Statistical analysis

Statistical analyses were performed using GraphPad Prism 9 (GraphPad Software Inc., San Diego, CA, USA). Data are presented as ± Standard Error of the Mean (SEM). Comparisons between the two groups were conducted using two-tailed Student’s t-tests, while multiple comparisons were analyzed by one-way ANOVA. Statistical significance was defined as *P* < 0.05, indicated by a single asterisk; double asterisks denote *P* < 0.01, and triple asterisks denote *P* < 0.001.

### TMRM/TMRE staining and imaging

TMRM (Thermo Fisher, T668) and TMRE (Thermo Fisher, T669) staining were performed as follows. A bacterial lawn was supplemented with TMRE or TMRE from a 10 mM stock solution to a final concentration of 5 μM and allowed to dry at room temperature for 2 h. Young adult worms were then transferred onto the stained lawn for 1 h. Following staining, animals were mounted and imaged using a 561-nm excitation laser under identical acquisition settings for all genotypes.

### Drug treatment

FCCP (Sigma C2920) was prepared as a 20 mM stock solution in ethanol. The drug was added to freshly seeded NGM plates from the stock solution to a final concentration of 10 μM and allowed to dry at room temperature for 1 h. Young adult worms were then transferred to the drug-containing plates and incubated for 1–2 h at room temperature before imaging. Fluorescence imaging was performed using a spinning-disk confocal microscope. GFP and RFP signals were excited with 488 nm and 561 nm lasers, respectively.

### Transmission electron microscopy (TEM)

*C. elegans* specimens were cryo-fixed using a high-pressure freezer (EM ICE, Leica, Germany). Freeze substitution was carried out in anhydrous acetone containing 2% osmium tetroxide. Samples were kept sequentially at −90 °C for 72 h, −60 °C for 10 h, and −30 °C for 10 h, and were finally brought to 0 °C for 5 h in a freeze-substitution unit EM AFS2 (Leica, Germany). Following substitution, samples were washed three times (10 minutes each) in cold anhydrous acetone and progressively infiltrated with Embed-812 resin. Resin polymerization was performed at 60 °C for 48 h. The embedded samples were cut into 70 nm sections using a microtome (EM UC7, Leica, Germany) and stained with uranyl acetate and lead citrate. Sections were observed using a transmission electron microscope (HT7800, Hitachi, Japan) operating at 80 kV.

### Reporting summary

Further information on research design is available in the Nature Portfolio Reporting Summary linked to this article.

## Supplementary information

Supplementary Information

Description of Additional Supplementary Files

Supplementary Movie 1

Supplementary Movie 2

Supplementary Movie 3

Supplementary Movie 4

Supplementary Movie 5

Supplementary Movie 6

Supplementary Movie 7

Reporting Summary

Transparent Peer Review file

## Source data

Source Data

## Data Availability

The authors declare that the data supporting the findings of this study are available within the paper. All data generated in this study are provided in the Source Data file. Source data are provided with this paper.
