# Supplementary material for: ATAD-3 couples plasma membrane injury to mitochondrial Ca2+ signaling
Source: Nat Commun. 2026 Sep 10;17:10690. doi: 10.1038/s41467-026-77690-y (PMC13655164; doi:10.1038/s41467-026-77690-y)
Supplement: Supplementary file 2 [file 41467_2026_77690_MOESM11_ESM.pdf]

nature portfolio

Peer Review File

ATAD-3 couples plasma membrane injury to mitochondrial Ca2+ signaling

Corresponding Author: Dr Suhong Xu

This file contains all reviewer reports in order by version, followed by all author rebuttals in order by version.

Version 0:

Reviewer comments:

Reviewer #1

(Remarks to the Author)
In this manuscript, Xu et al. describe a wound induced delayed mitochondrial Ca2+ elevation in C. elegans that occurs independent of the classical mitochondrial Ca2+ uptake pathway, as has been shown in other model organisms. Using a genetic screening approach, the authors show that knocking down the expression of Atad-3 strongly blunts mitochondrial uptake. The authors demonstrate that this effect occurs independent of the MCU, as MCU mutants also show an Atad-3 dependent knockdown. The authors show that Atad-3 forms oligomers shortly after wound induction. The authors propose that this oligomerization is required for Ca2+ uptake, as mutations which disrupt oligomerization were associated with reduced mitochondrial Ca2+ uptake.

Overall Comments:

As stated by the authors, the identification of an MCU-independent pathway for mitochondrial Ca2+ uptake would have substantial significance for understanding mitochondrial metabolism and dysfunction in disease. The authors provide robust demonstration of an MCU independent Ca2+ elevation in C. elegans after wound induction. They also effectively show that Atad-3 disruption, either by knockdown or mutation, leads to the disruption of the MCU-independent Ca2+ elevation. These aspects of the manuscript are well-founded, although some additional validations would be helpful (see individual comments below). However, an important aspect of this manuscript, with respect to impact, is the proof that Atad-3 is a bonafide mediator of mitochondrial Ca2+ uptake. The authors repeatedly suggest that Atad-3 “mediates” Ca2+ uptake into the mitochondria. While Atad-3 may indeed act as a direct mediator, sufficient proof has not been provided in the manuscript to warrant such a description. A direct role in mediating Ca2+ uptake insinuates that Atad-3 serves as a Ca2+-permeable ion channel or immediate regulator of one. Aside from Atad-3 acting as an ion channel or immediate regulator, there are several indirect mechanisms by which Atad-3 disruption could alter mitochondrial Ca2+ uptake. For example, disruptions in mitochondrial membrane potential, protein import, mitochondrial morphology and network stability. Indeed, the authors mention that Atad-3 is known to be involved in mitochondrial membrane organization, cristae maintenance, and mtDNA stability. The authors show predicted structural features of the Atad-3 hexameric structure with a pore-like structure, suggesting that it may serve as an ion channel for passing Ca2+. However, there is no mention that other AAA ATPases have a similar pore structure, but which functions to transport/unfold proteins. If the mechanism by which Atad-3 disrupts mitochondrial Ca2+ elevations is through such indirect mechanisms, the results showing a disruption of mitochondrial Ca2+ uptake are still valid and meaningful. However, the interpretation and significance of the results would be diminished, as the protein would not be considered a legitimate conduit for mitochondrial Ca2+ uptake- the identification of which was part of the research objectives. These important issues can be addressed by examining whether Atad-3 impacts the aforementioned indirect mechanisms of regulating mitochondrial Ca2+ uptake. Also, the authors could provide more functional evidence of Atad-3 operating as a channel or direct regulator of a channel.

Major comments:

1) Atad-3 knockdown resulted in a significant reduction in the mitochondrial Ca2+ elevation at both late and early time points (Panel 2F). This is interesting, as the MCU independent Ca2+ elevation occurs at later time points in Figure 1 and Figure S1D. Figure S1D also shows that MCU knockdown strongly reduces the early phase of mitochondrial Ca2+ uptake. This suggests a potential interaction between the mechanisms underlying Atad-3 and MCU dependent Ca2+ uptake mechanisms. For example, direct interaction with the MCU, or indirect, by altering the mitochondrial membrane potential

(driving force for Ca2+ uptake). Please comment on this.

2) Genetically encoded sensors can sometimes mislocalize to different cellular compartments. For example, a fraction of the mitochondrial GCAMP may be present in non-mitochondrial compartments. If this occurs, then it could account for an MCU-independent Ca2+ response. The authors should perform experiments demonstrating that the mitochondrial GCAMP responses occur in a manner consistent with a highly specific mitochondrial localization. For example, demonstrating a dependence of the Ca2+ response on the mitochondrial membrane potential.

3) The authors note that there was no impact of Atad-3 knockdown on baseline mitochondrial Ca2+ (Figure 2C). However, it seems that in most of the images of the Atad-3 knockdown or mutants (Fig 2E,G & Fig 3A,C), there was a noticeable region of elevated mitochondrial Ca2+ (around the midline of the animal) that is not apparent in the wildtype animals. Can the authors comment on this please?

4) Western blot data shows that Atad-3-GFP increased in Mcu-1 mutants, 5 minutes after wounding (Figure 4B and 4C). Is this total cellular Atad-3-GFP content? A better option would be to show mitochondrial Atad-3-GFP content, as this would be a better proof for Atad-3-GFP accumulation. If it is total cell content, what is the explanation for this increase in cellular Atad-3-GFP?

5) If Atad-3 is disrupting mitochondrial Ca2+ uptake, we would expect altered metabolic functon(e.g. mito ROS generation) and wound repair, as seen in prior publications (Xu et al 2014). The authors do not make these measurements. This would increase support for an important functional impact of the MCU-independent pathway.

6) Confirmation of the disruption of oligomerization is very important to Figure 5. The western blots showing disruption of oligomerization should be moved to the main Figure out of the supplementary material. Also, the authors will need to rule out other potential explanations for the disrupted mitochondrial Ca2+ uptake aside from altered oligomerization. For example, is the protein still appropriately targeted to the mitochondria? This could be addressed with imaging of Atad-3 cellular localization as done with other experiments.

7) As described in the overall comments section, please examine potential mechanisms by which Atad-3 could be disrupting mitochondrial Ca2+ elevation- e.g. mitochondrial membrane potential, integrity, network.

Minor comments:

8) I am not familiar with the method used for RNAi screening (e.g. the use of the rde-1 mutant animals). The authors referred to prior publications on this method. Please provide more detail on this in the methods section as this is an important aspect of the paper and may not be familiar to some readers less familiar with the C. elegans model.

9) The authors use a MCU mutant strain with excised exons to produce knockdown. A critical aspect of their research is the assumption that the MCU protein is non-functional. This is critical and should be expanded on either in discussion or with experimental validation. As far as I have seen, there is no validation or references to this validation elsewhere.

10) Please review the references to Figure 5 in the text of the article. They do not match with the content of the figure panels.

11) It appears that there are no statistical summaries for the data in Figure 5. Please address this.

12) Often the figure legends lacked sufficient information describing the statistical methods. Please address this.

13) Figure 1: UW vs W (it is not clear what UW and W refer to in the figure or figure legend) please change the description to baseline and wound, or describe the UW and W acronym in the figure legend.

14) Fig 1 Panel B: please provide a colour code scale bar, it is obvious warm colours are greater Ca2+, but this should still be shown.

15) Scale bar unit missing Figure 5B.

16) Figure 5A,B: The transmembrane domain is labeled in dark blue in panel A, but in panel B this colour is not present. There is a red label on the predicted 3d protein structure in panel B- I presume that this is supposed to be the transmembrane domain labelled in blue (not red)? Please fix colour coding.

Reviewer #2

(Remarks to the Author)
I co-reviewed this manuscript with one of the reviewers who provided the listed reports. This is part of the Nature Communications initiative to facilitate training in peer review and to provide appropriate recognition for Early Career Researchers who co-review manuscripts.

Reviewer #3

(Remarks to the Author)
Xu et al. observed that tissue wounding in C. elegans induces mitochondrial calcium signaling independent of the mitochondrial calcium uniporter (MCU). Through an RNAi screen, they identified ATAD3 and MICU3 as key regulators of this pathway. The authors further demonstrated that wounding promotes ATAD3 oligomerization, which is essential for facilitating MCU-independent mitochondrial calcium uptake. The results are interesting but lacking in rigor to really prove that ATAD3 is a calcium uptake channel.

Major comments:
1. In the lab's previously published article (https://doi.org/10.1016/j.devcel.2014.08.002), local mtROS production was observed following laser wounding. In the current manuscript, the authors employed a higher-energy laser for wounding, which could potentially induce greater mtROS production. This raises concern that excessive mtROS may lead to mitochondrial swelling, thereby affecting the accuracy of mitoGCaMP5 signals. To address this, it would be helpful for the authors to assess mtROS level and mitochondrial membrane potential following high-energy laser wounding to confirm that mitochondrial function remains intact and that mitoGCaMP5 readings are reliable.
2. In Figure 1, the authors utilized three different wounding methods: high-energy laser, needle, and ultrasound-induced wounding. In Figure 2, it appears that both needle and laser wounding were used. However, in the remaining figures, the wounding method is not clearly identified, though it seems laser wounding was predominantly used. First, please clearly indicate the wounding method used in each figure and panel to avoid confusion. Second, for the RNAi screen, the authors used needle wounding, while in most other experiments laser wounding appears to be employed. This inconsistency needs to be addressed. Please either standardize the wounding method across experiments or provide a detailed rationale for using different wounding techniques in different parts of the study.
3. It is very interesting that the MICU3 mutant increases basal mitochondrial calcium levels, and that this effect can be suppressed by overexpression of MICU2 or MICU3. MICU2 and MICU3 are known to form heterodimers with MICU1 to enhance MCU-dependent mitochondrial calcium uptake. Have the authors examined MICU1 expression levels in the MICU3 mutant group? Additionally, the authors state that MICU3 interacts with MCU-1; however, according to the literature, this interaction is typically mediated through MICU1. It would be important to assess MICU1–MICU3 binding directly and to determine how this interaction influences MCU-1 under both baseline and wounding conditions.
4. While the authors clearly demonstrate a role for ATAD3 in MCU-independent mitochondrial calcium uptake, previous studies have already shown that ATAD3 interacts with SIRT3 to regulate MAM dynamics, which in turn influence mitochondrial calcium homeostasis independently of MCU (DOI: 10.7150/ijbs.89253). These studies also indicate that ATAD3 oligomerization is disrupted by acetylation, and that ATAD3 monomer interacts with the IP3R1–GRP75–VDAC1 complex. Given these findings, the authors should examine SIRT3 levels and acetylation status in their wounding model to determine whether increased acetylation disrupts ATAD3 oligomerization, thereby preventing mitochondrial calcium uptake.
5. What is the phenotype of atad-3 knockdown (loxP) in the epidermal cells on mitochondrial viability and tissue viability over a time course? The concern is the interpretation that atad-3 RNAi or the loxP-KO is otherwise compromising mitochondria structure-function and causing cellular damage, given its known role in regulating inner and outer mitochondrial membrane apposition and greater effect this would have on all mitochondrial processes, hence leading to a misinterpretation that atad3 is a calcium channel. Does epidermal deletion of atad3 reduce mitochondrial respiration, or other functions? Somehow the authors need to rule out the very simple interpretation that atad-3 loss compromises mitochondria structure-function more globally so that they cannot take up calcium given other major structural defects (as they authors state, germline deletion of atad3 is lethal). As an aside, what about deleting components of the MICOS complex in epidermal cells, especially MIC60, which also directly regulates inner and outer mito membrane coordination and structural alignment? Would loss of MICOS complex also lead to loss of wounding induced calcium entry? Deletion of genes that result in the loss of the MICOS complex is also known to be lethal. Mitochondrial permeability transition that also lets in calcium, or lets it out, requires correct apposition of the inner and outer mito membranes.
6. There is a complete absence of data showing the architectural or structural effects of the atad3 knockdown or epidermal KO at the level of the mitochondria (electron microscopy). This is even more important to show for all the mutants in atad3 that were used, some of which prevented oligomerization and the calcium defect, some of which did not. Does deletion of this gene or some of the mutants correlate with a more general loss of mitochondrial cristae organization and the proper stacking of the inner and outer mito membranes? Does it correlate with mitochondrial respiration and function? How about blue native gels to show the respiratory complexes are in tact?

Minor comments:
1. Please define MCU-1when it was first described in the main context.
2. In Figure 1 and S1, the authors mentioned low-energy and high-energy laser wounding. However, the precise description to distinguish between two procedures was missing in the methods. Was the duration, intensity, or other indexes being different?
3. Please correct the typo of Figure legend 1G: should be 15 min not 5min.
4. For the surface plots shown in Fig. 1F and Fig. S1E, the authors presented three time points: 5 s, 30 s, and 120 s post-wounding. It would be beneficial to combine these panels into a single composite figure or time-course plot to better visualize the dynamic changes and trends in mitochondrial calcium patterns across different groups following wounding.
5. Please correct the figure legend for Fig. 2C-D, 4C: the panel doesn't match the description in the figure legend.

Version 1:

Reviewer comments:

Reviewer #1

(Remarks to the Author)
Overall, the authors addressed a number of the issues noted at the first review. However, there are still some aspects of the study which require more clarification. Please the comments below.

Comments:
1) ATAD-3 disruption reduces wound induced mitochondrial Ca2+ uptake, but the recent results show that there is no impact on mitochondrial membrane potential, mitochondrial ROS, and wound repair dynamics. This is surprising given the known link between mitochondrial Ca2+ uptake and mito membrane depolarization. It is also surprising considering that the new data in the revision suggests that ATAD3 affects the early MCU-dependent Ca2+ uptake, which was shown to impact mito ROS and wound healing in a prior publication. Can you comment on this? The lack of impact of ATAD3 on ROS generation and wound healing are relevant to the impact of the paper, as they are physiological readouts expected to be impacted by mitochondrial Ca2+. This data suggests that Atad3 may impact mitochondrial Ca2+ uptake during wound responses, but that this process may have other roles aside from regulating metabolism. It would be helpful for this data to be presented and discussed in this paper in order to understand the physiological relevance of Atad3.
2) The new results showing Atad3 accumulation in isolated mitochondria is helpful data. In some ways, it is more relevant and easier to understand than the whole-worm Atad3 measurements post-wounding. It is still unclear to me how the whole-worm Atad3 protein abundance increases following 5 minutes of wound induction. It is also unclear to me how the authors relate an increase in whole-worm Atad3 to the greater mitochondrial Atad3 accumulation/oligomerization. Can you provide more clarity on this and revise accordingly?
3) The authors provide new data showing that the subcellular localization of ATAD3 in oligomerization deficient ATAD-3 mutants is not significantly altered in unwounded conditions. However, it would also be useful to see whether the localization/clustering of ATAD3 on mitochondria during wound induction is affected in these oligomerization deficient mutants.

Reviewer #2

(Remarks to the Author)
I co-reviewed this manuscript with one of the reviewers who provided the listed reports. This is part of the Nature Communications initiative to facilitate training in peer review and to provide appropriate recognition for Early Career Researchers who co-review manuscripts.

Reviewer #3

(Remarks to the Author)
Most of the previous concerns have been addressed. However, comment #2 still needs to be resolved. While the authors explained the rationale for the different wounding methods in their response, the context within the manuscript remains unclear. Regarding laser wounding, the authors now use the terms "small-scale" versus "large-scale." Please include definitions for these two scales (e.g., differences in time, intensity, etc.) in the Methods section under “Wounding Procedures.”

Additionally, please include the justification for choosing needle wounding for the RNAi screening in the Methods section. Furthermore, in line 580, Fig. 2b appears to be needle wounding, yet the figure legend refers to "large-scale laser wounding." Please clarify and correct it.

Also, in Fig. 6, please indicate the mitochondria and cytosol. Based on the figure legend, please also indicate MICU3.

Finally, the TEM images in Supp. Fig. 2b are not high-resolution, and the cristae structures are barely visible. Please replace these with high-resolution images.

Version 2:

Reviewer comments:

Reviewer #1

(Remarks to the Author)
Thank you for the responses. Please address these issues:

1) Line 3 and 4 of the abstract: “ Here, we identify an alternative mitochondrial Ca2+ uptake pathway mediated by ….ATAD-3.” As mentioned in previous reviews, this is a strong statement that is not proven in this paper, as it is yet uncertain if ATAD-3 is a bona fide conduit for Ca2+ entry. Please adjust here and throughout the paper accordingly.

2) In the rebuttal letter, response to comment #3. The authors indicate that “In WT animals, wounding triggers a rapid and

pronounced increase in ATAD-3::GFP clustering on mitochondria, manifesting as discrete, high-intensity foci on the mitochondrial surface that were distinct from the even mitochondrial distribution observed in unwounded animals. In contrast, ATAD-3(Δ240)::GFP failed to undergo injury-induced mitochondrial clustering, despite normal baseline co-localization with mito::mKate2 (Response Fig. 1). The intramitochondrial intensity distribution of these mutants remains uniform and indistinguishable from their pre-wound pattern following wounding. As these data were acquired by HiS-SIM images, which preclude robust quantitative analysis, we present representative images to illustrate the qualitative difference."

It is not clear to me how the authors made this interpretation from the images presebted. In WT animals, the ring-like ATAD3 clustering around the mitochondria is apparent and seems to be more intense than in unwounded conditions. In the mutant animal, it also appears that ATAD3 forms clusters in ring-like structures around the mitochondria following wounding, although seemingly, with lower intensity. As there was no quantification performed, it is difficult to assess if this intensity difference is real or not. The clustering distribution does not look appreciably different by eye as well.

Reviewer #2

(Remarks to the Author)
I co-reviewed this manuscript with one of the reviewers who provided the listed reports. This is part of the Nature Communications initiative to facilitate training in peer review and to provide appropriate recognition for Early Career Researchers who co-review manuscripts.

Reviewer #3

(Remarks to the Author)
Revisions are adequate

Version 3:

Reviewer comments:

Reviewer #1

(Remarks to the Author)
The authors have adequately addressed my concerns. Thank you.

Reviewer #2

(Remarks to the Author)
I co-reviewed this manuscript with one of the reviewers who provided the listed reports. This is part of the Nature Communications initiative to facilitate training in peer review and to provide appropriate recognition for Early Career Researchers who co-review manuscripts.

Open Access This Peer Review File is licensed under a Creative Commons Attribution 4.0 International License, which permits use, sharing, adaptation, distribution and reproduction in any medium or format, as long as you give appropriate credit to the original author(s) and the source, provide a link to the Creative Commons license, and indicate if changes were made.
In cases where reviewers are anonymous, credit should be given to 'Anonymous Referee' and the source.
The images or other third party material in this Peer Review File are included in the article's Creative Commons license, unless indicated otherwise in a credit line to the material. If material is not included in the article's Creative Commons license and your intended use is not permitted by statutory regulation or exceeds the permitted use, you will need to obtain permission directly from the copyright holder.
To view a copy of this license, visit https://creativecommons.org/licenses/by/4.0/

Reviewer comments (our point-by-point responses are included in blue text):

Reviewer #1 (Remarks to the Author):

In this manuscript, Xu et al. describe a wound induced delayed mitochondrial Ca2+ elevation in C. elegans that occurs independent of the classical mitochondrial Ca2+ uptake pathway, as has been shown in other model organisms. Using a genetic screening approach, the authors show that knocking down the expression of Atad-3 strongly blunts mitochondrial uptake. The authors demonstrate that this effect occurs independent of the MCU, as MCU mutants also show an Atad-3 dependent knockdown. The authors show that Atad-3 forms oligomers shortly after wound induction. The authors propose that this oligomerization is required for Ca2+ uptake, as mutations which disrupt oligomerization were associated with reduced mitochondrial Ca2+ uptake.

Overall Comments:

As stated by the authors, the identification of an MCU-independent pathway for mitochondrial Ca2+ uptake would have substantial significance for understanding mitochondrial metabolism and dysfunction in disease. The authors provide robust demonstration of an MCU independent Ca2+ elevation in C. elegans after wound induction. They also effectively show that Atad-3 disruption, either by knockdown or mutation, leads to the disruption of the MCU-independent Ca2+ elevation. These aspects of the manuscript are well-founded, although some additional validations would be helpful (see individual comments below). However, an important aspect of this manuscript, with respect to impact, is the proof that Atad-3 is a bonafide mediator of mitochondrial Ca2+ uptake. The authors repeatedly suggest that Atad-3 “mediates” Ca2+ uptake into the mitochondria. While Atad-3 may indeed act as a direct mediator, sufficient proof has not been provided in the manuscript to warrant such a description.

A direct role in mediating Ca2+ uptake insinuates that Atad-3 serves as a Ca2+-permeable ion channel or immediate regulator of one. Aside from Atad-3 acting as an ion channel or immediate

regulator, there are several indirect mechanisms by which Atad-3 disruption could alter mitochondrial Ca2+ uptake. For example, disruptions in mitochondrial membrane potential, protein import, mitochondrial morphology and network stability. Indeed, the authors mention that Atad-3 is known to be involved in mitochondrial membrane organization, cristae maintenance, and mtDNA stability. The authors show predicted structural features of the Atad-3 hexameric structure with a pore-like structure, suggesting that it may serve as an ion channel for passing Ca2+. However, there is no mention that other AAA ATPases have a similar pore structure, but which functions to transport/unfold proteins. If the mechanism by which Atad-3 disrupts mitochondrial Ca2+ elevations is through such indirect mechanisms, the results showing a disruption of mitochondrial Ca2+ uptake are still valid and meaningful. However, the interpretation and significance of the results would be diminished, as the protein would not be considered a legitimate conduit for mitochondrial Ca2+ uptake- the identification of which was part of the research objectives. These important issues can be addressed by examining whether Atad-3 impacts the aforementioned indirect mechanisms of regulating mitochondrial Ca2+ uptake. Also, the authors could provide more functional evidence of Atad-3 operating as a channel or direct regulator of a channel.

Response:

We thank the reviewer for this thoughtful overall assessment of our study and for recognizing both the robustness of our identification of an MCU-independent mitochondrial Ca^{2+} uptake pathway and the significance of defining ATAD-3's role in this process. We agree that while our data establish ATAD-3 as an essential regulator of injury-induced mitochondrial Ca^{2+} entry, they do not yet provide definitive evidence that ATAD-3 itself functions as a Ca^{2+}-permeable ion channel. We therefore carefully revised both our interpretation and wording to avoid overstatement.

In the revised manuscript, we have replaced language implying that ATAD-3 "mediates" or "forms" a Ca^{2+} channel with more accurate phrasing, including "ATAD-3 is required for," "regulates," or "enables" mitochondrial Ca^{2+} uptake. We now explicitly state in the Discussion that, while structural modeling predicts a pore-like oligomeric assembly, we do not yet claim that

ATAD-3 constitutes a Ca^{2+} channel, but rather that it acts as a critical regulator of mitochondrial Ca^{2+} uptake during injury.

We agree with the Reviewer's comments that disruption of mitochondrial Ca^{2+} uptake could theoretically arise from indirect effects on mitochondrial physiology, including membrane potential, protein import, membrane organization, or network integrity. To address these possibilities, we performed additional experiments and analyses, now included in the revised manuscript:

1. We confirmed specific mitochondrial localization of mito::GCaMP5 by co-localization with TOMM-20::mKate2 and TMRM under basal and wounded conditions, ensuring that recorded Ca^{2+} signals reflect genuine mitochondrial Ca^{2+} dynamics.
2. TMRE imaging revealed that Δψm is comparable between wild-type and *atad-3*–deficient epidermal cells under basal conditions and decreases similarly after wounding. Thus, impaired Ca^{2+} uptake is not due to loss of the electrochemical driving force.
3. Confocal imaging and transmission electron microscopy demonstrate that *atad-3* deficiency causes moderate mitochondrial fragmentation and partial cristae disorganization, but not catastrophic membrane rupture or collapse. Importantly, comparable or stronger architectural disruption (e.g., MICOS or *fzo*-1 perturbation) does not abolish wound-induced mitochondrial Ca^{2+} uptake, indicating that general structural defects alone cannot explain the *atad-3* phenotype.
4. Both wild-type and oligomerization-deficient ATAD-3 variants localize correctly to mitochondria, demonstrating that loss of Ca^{2+} uptake in these mutants is not due to mislocalization.

Based on these new data, we now present a refined model: ATAD-3 is a context-dependent regulator of mitochondrial Ca^{2+} uptake. Under basal conditions, it supports efficient MCU-dependent Ca^{2+} entry. Following membrane injury, ATAD-3 undergoes oligomerization and enables an MCU-independent Ca^{2+} uptake pathway. While structural modeling suggests that oligomerization could form a pore-like assembly, we explicitly state that future biochemical and electrophysiological studies will be required to determine whether ATAD-3 directly conducts Ca^{2+}or regulates another Ca^{2}-permeable entity.

We believe these revisions directly address the reviewer's concerns and substantially strengthen the rigor and balance of the manuscript.

Major comments:

1) Atad-3 knockdown resulted in a significant reduction in the mitochondrial Ca2+ elevation at both late and early time points (Panel 2F). This is interesting, as the MCU independent Ca2+ elevation occurs at later time points in Figure 1 and Figure S1D. Figure S1D also shows that MCU knockdown strongly reduces the early phase of mitochondrial Ca2+ uptake. This suggests a potential interaction between the mechanisms underlying Atad-3 and MCU dependent Ca2+ uptake mechanisms. For example, direct interaction with the MCU, or indirect, by altering the mitochondrial membrane potential (driving force for Ca2+ uptake). Please comment on this.

Response:

We thank the reviewer for this insightful and constructive comment. We agree that the reduction of mitochondrial Ca^{2+} elevation at both early and late time points upon *atad-3* knockdown suggests a potential functional relationship between ATAD-3 and MCU-dependent Ca^{2+} uptake. Guided by the reviewer's suggestions, we have performed additional genetic, biochemical, and imaging experiments to clarify this relationship.

First, our genetic analyses indicate that ATAD-3 is required for efficient MCU-1-dependent Ca^{2+} uptake under physiological conditions. In epidermal-specific *atad-3* conditional knockout (cKO) animals, wounding failed to induce robust mitochondrial Ca^{2+} uptake (Fig. 2b-2f), indicating that MCU–dependent Ca^{2+} entry is also largely compromised in the absence of ATAD-3. Consistent with this, knockdown of *atad-3* in *micu-3* mutants completely abolished the elevated basal mitochondrial Ca^{2+} levels characteristic of *micu-3* single mutants (Fig. 3a and 3b), indicating that ATAD-3 is necessary for MCU-driven Ca^{2+} uptake under normal conditions.

Second, we tested whether ATAD-3 regulates mitochondrial Ca^{2+} through direct physical interaction with MCU-1. The co-IP experiments using endogenously tagged proteins did not detect an association between ATAD-3 and MCU-1(Fig. 3e). Moreover, knockdown of *atad-3* did not alter MCU-1::GFP expression levels or its mitochondrial localization (Supplementary

Fig. 5a and 5b). These data argue against a direct structural role for ATAD-3 within the MCU-1. Instead, we find that ATAD-3 interacts with the outer mitochondrial membrane protein VDAC-1 (Fig. 3g), which itself associates with MCU-1 *in vivo* (Fig. 3e and 3f). This suggests that ATAD-3 may influence MCU-1-dependent Ca^{2+} uptake indirectly through modulation of mitochondrial membrane interface components rather than by binding the uniporter complex itself.

Third, we examined whether ATAD-3 affects mitochondrial Ca^{2+} uptake by altering mitochondrial membrane potential ($\Delta\psi m$), which may provide the driving force for Ca^{2+} entry, as inspired by the reviewer's comments. TMRE measurements reveal comparable $\Delta\psi m$ in WT and *atad-3* cKO animals under basal conditions, and both genotypes exhibited similar reductions in $\Delta\psi m$ following wounding (Supplementary Fig. 6c). Thus, impaired Ca^{2+} uptake in *atad-3*-deficient animals is unlikely to result from altered membrane potential. We have included these new data in the revised supplementary Figures and refer to them in the results section.

Finally, we find that *atad-3* knockdown in *mcu-1* mutants further reduced wound-induced mitochondrial Ca^{2+} signals to levels significantly lower than those observed in *mcu-1* single mutants (Fig. 3c and 3d). This observation indicates that ATAD-3 also contributes to mitochondrial Ca^{2+} uptake through an MCU-independent mechanism that becomes evident when MCU-1 is absent.

Taken together, these new data support a model in which ATAD-3 plays a dual role in mitochondrial Ca^{2+} regulation: under normal conditions, it enables efficient MCU-1-dependent Ca^{2+} uptake. While under injury or MCU-deficient conditions, ATAD-3 mediates an alternative Ca^{2+} entry pathway. We have revised the manuscript accordingly and now propose ATAD-3 as a context-dependent regulator of mitochondrial Ca^{2+} uptake.

In response to the reviewer's helpful suggestion, we have also refined the manuscript title to better reflect this concept: "ATAD-3 couples plasma membrane injury to mitochondrial Ca^{2+} signaling".

2) Genetically encoded sensors can sometimes mislocalize to different cellular compartments. For example, a fraction of the mitochondrial GCAMP may be present in non-mitochondrial

compartments. If this occurs, then it could account for an MCU-independent Ca2+ response. The authors should perform experiments demonstrating that the mitochondrial GCAMP responses occur in a manner consistent with a highly specific mitochondrial localization. For example, demonstrating a dependence of the Ca2+ response on the mitochondrial membrane potential.

Response:
We thank the reviewer for this important concern regarding the specificity of genetically encoded Ca^{2+} sensors. To rigorously verify that the mito::GCaMP5 signals reflect bona fide mitochondrial Ca^{2+} dynamics rather than Ca^{2+} changes in non-mitochondrial compartments, we performed additional functional validation experiments.

First, we verified subcellular localization of mito::GCaMP5 using independent mitochondrial markers. In mito::GCaMP5 transgenic animals, fluorescence exhibited strong colocalization with TMRM, a mitochondrial membrane potential-dependent dye, as well as Tomm-20::mKate2, a mitochondrial outer membrane marker, both before and after wounding (Supplementary Fig. 1e and Fig 1f). No detectable cytosolic or non-mitochondrial GCaMP signal was observed. These results confirm that mito::GCaMP5 is specifically and stably localized to mitochondria under both basal and injury conditions.

Second, we tested whether the observed Ca^{2+} signal behaves in a manner consistent with genuine mitochondrial Ca^{2+} uptake. Dual imaging of mito::GCaMP5 and membrane potential-sensitive dye TMRM revealed fully colocalized signals before wounding. Upon wounding, TMRM decreased sharply while mito::GCaMP5 signals increased robustly (Supplementary Fig. 1e). Importantly, the spatial pattern of Ca^{2+} elevation remained restricted to mitochondrial structures throughout the response. This behavior is inconsistent with cytosolic Ca^{2+} contamination and supports a mitochondrial origin of the signal.

Finally, we independently confirmed the mitochondrial Ca^{2+} uptake phenomenon using a second genetically encoded mitochondrial Ca^{2+} reporter (mito::NEMOer), which produced identical response kinetics in *mcu-1* mutants (Supplementary Fig. 1g). This provides further assurance that our observations are not attributable to sensor mislocalization.

Together, these localization and functional controls demonstrate that the Ca^{2+} transients reported by mito::GCaMP5 represent bona fide mitochondrial Ca^{2+} uptake, and not artifactual signals arising from mislocalized sensor pools. We have included these data and clarification in the revised supplementary Figures and refer to them in the results section.

3) The authors note that there was no impact of Atad-3 knockdown on baseline mitochondrial Ca2+ (Figure 2C). However, it seems that in most of the images of the Atad-3 knockdown or mutants (Fig 2E,G & Fig 3A,C), there was a noticeable region of elevated mitochondrial Ca2+ (around the midline of the animal) that is not apparent in the wildtype animals. Can the authors comment on this please?

Response:

We thank the reviewer for this careful observation. The region displaying relatively elevated mitochondrial Ca^{2+} signals in *atad-3* knockdown or mutant animals corresponds anatomically to the seam cell, which lies along the lateral middle of the animal and is distinct from the large multinucleated epidermal syncytium (hyp7), the primary tissue analyzed in our wounding assays.

Using an endogenous ATAD-3::GFP knock-in strain, we found that ATAD-3 is highly expressed in hyp7 cells but is barely detectable in seam cells (Supplementary Fig. 4j). This expression pattern suggests that ATAD-3 functions predominantly in hyp7 cells, where we perform all quantitatively analyses of injury-induced mitochondrial Ca^{2+} uptake.

Consistent with this, epidermal-specific RNAi and hyp7-restricted conditional knockout of *atad-3* robustly reduced mito::GCaMP5 signals in hyp7 cells, while mitochondrial Ca^{2+} signals in seam cells were largely unaffected. Thus, the relatively higher Ca^{2+} observed along the lateral midline in *atad-3* perturbed animals reflects the fact that seam cell mitochondria are not efficiently targeted by our epidermal manipulations.

4) Western blot data shows that Atad-3-GFP increased in Mcu-1 mutants, 5 minutes after wounding (Figure 4B and 4C). Is this total cellular Atad-3-GFP content? A better option would be to show mitochondrial Atad-3-GFP content, as this would be a better proof for Atad-3-GFP accumulation. If it is total cell content, what is the explanation for this increase in cellular Atad-

3-GFP?

Response:

We thank the reviewer for this important suggestion. In the original presented data, the immunoblots in Fig. 4b and 4c were performed using whole-worm lysates and therefore reflect total cellular ATAD-3::GFP levels.

Following the reviewer's recommendation, we have now performed additional biochemical fractionation to directly examine mitochondrial ATAD-3::GFP. Mitochondrial fractions were isolated from endogenous ATAD-3::GFP knock-in animals before and after wounding in the *mcu-1* mutant background. These new data show that ATAD-3::GFP levels are specifically increased in purified mitochondrial fractions after wounding in the *mcu-1* mutants. These results demonstrate that the observed increase in ATAD-3::GFP reflects enhanced mitochondrial accumulation rather than a global rise in cellular protein abundance. We have included these data in the revised manuscript (Supplementary Fig. 7b).

Regarding the mechanism underlying this increase, we note that the ATAD-3::GFP transgene is expressed from the endogenous locus, and the time scale of the response (within 5 minutes after wounding) is too rapid to be explained by new protein synthesis. We therefore interpret this increase as reflecting acute accumulation of mitochondrial ATAD-3, likely due to enhanced mitochondrial association or reduced turnover following injury.

Together, these additional experiments strengthen the conclusion that cellular injury triggers acute accumulation of ATAD-3 on mitochondria when MCU-1-mediated Ca^{2+} uptake is compromised, thereby supporting its proposed role as an injury-responsive regulator of mitochondrial Ca^{2+} uptake.

5) If Atad-3 is disrupting mitochondrial Ca2+ uptake, we would expect altered metabolic functon(e.g. mito ROS generation) and wound repair, as seen in prior publications (Xu et al 2014). The authors do not make these measurements. This would increase support for an

important functional impact of the MCU-independent pathway.

Response:

We thank the reviewer for this helpful suggestion regarding the potential functional consequences of ATAD-3-mediated mitochondrial Ca^{2+} regulation. To address this point, we performed additional experiments to assess whether disruption of ATAD-3-dependent mitochondrial Ca^{2+} uptake leads to detectable alterations in mitochondrial metabolism and wound repair.

First, we examined mitochondrial ROS production using MitoSOX staining in epidermal cells after wounding. Surprisingly, we found no significant difference in mitochondrial ROS levels between control and epidermal-specific *atad-3* conditional knockout animals after injury (Response Fig. 1a). This data indicates that loss of ATAD-3–dependent Ca^{2+} uptake does not overtly perturb mitochondrial redox homeostasis in this tissue.

Second, we assessed epidermal wound repair dynamics in *atad-3*-depleted animals by quantifying wound closure dynamics and actin ring formation at defined time points after injury. Consistent with mitochondrial ROS levels, no significant delay or morphological abnormalities were detected compared to WT (Response Fig. 1b). Thus, despite the clear requirement for ATAD-3 in injury-induced mitochondrial Ca^{2+} uptake, epidermal wound repair proceeds normally under the conditions tested.

Together, these findings suggest that the ATAD-3-dependent Ca^{2+} entry pathway uncovered in this study is not obligatorily coupled to mitochondrial ROS production or actin-mediated wound closure in the *C. elegans* epidermis. Notably, this differs from prior observations that disruption of MCU-1-dependent mitochondrial Ca^{2+} uptake impairs ROS generation and wound repair. This distinction suggests that distinct mitochondrial Ca^{2+} entry routes, and the magnitude, timing, or spatial distribution of the resulting Ca^{2+} signals, may engage downstream metabolic and signaling pathways in a context-dependent manner.

One possible explanation is that ATAD-3-dependent Ca^{2+} influx represents a transient or spatially restricted signal that is sufficient to indicate mitochondrial Ca^{2+} entry but insufficient to drive

bulk metabolic activation or ROS production. In addition, because ATAD-3 also contributes to mitochondrial morphology and dynamics, ATAD-3-dependent Ca^{2+} entry may serve functions distinct from acute ROS generation, such as regulating mitochondrial ion homeostasis, matrix signaling, or longer-term mitochondrial adaptation to injury.

Because these additional assays did not reveal functional defects that alter the central mechanistic conclusions of the manuscript, we have not included them in the revised main text, but we are happy to provide the data upon request.

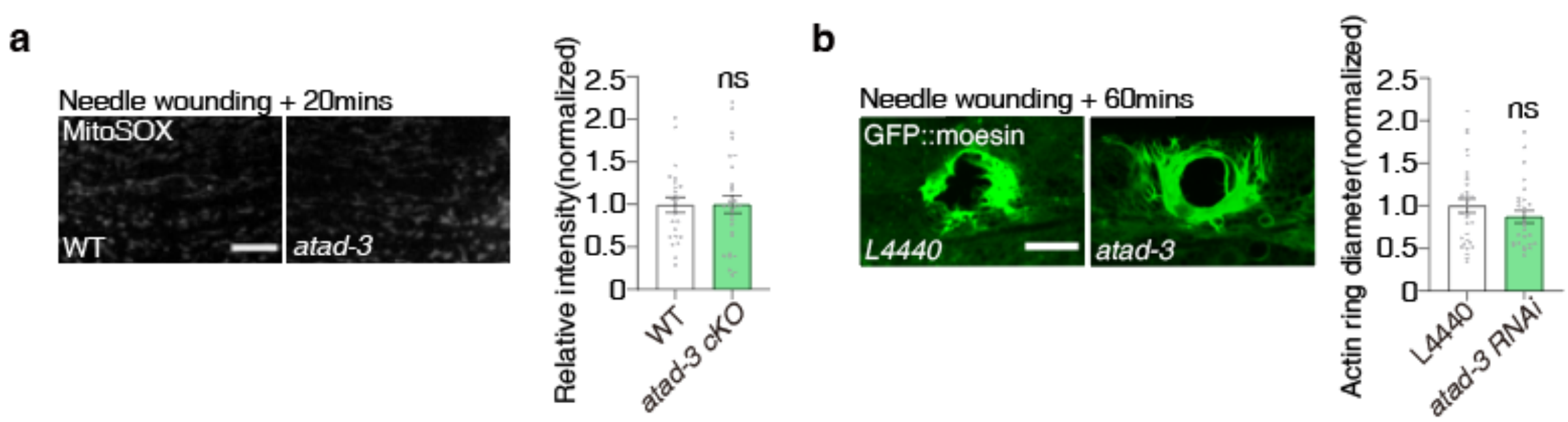

Response Fig. 1

Response Fig. 1 ATAD-3 is not required for wounding-induced mitochondrial ROS elevation or actin polymerization. (a) Left: Confocal images of mitoSOX staining in wild-type and epidermal-specific *atad*-3 knockout animals after needle wounding. Scale: 10 μm. Right: Quantification of mitoSOX intensity. n ⩾ 22 samples. ns, $P > 0.05$, Student's t-test. (b) Left: Confocal images of GFP::moesin assembly after needle wounding in *atad*-3 RNAi and control animals. Scale: 10 μm. Right: Quantification of actin ring diameter. n ⩾ 23 samples. ns, $P > 0.05$, Student's t-test.

6) Confirmation of the disruption of oligomerization is very important to Figure 5. The western blots showing disruption of oligomerization should be moved to the main Figure out of the supplementary material. Also, the authors will need to rule out other potential explanations for the disrupted mitochondrial Ca2+ uptake aside from altered oligomerization. For example, is the protein still appropriately targeted to the mitochondria? This could be addressed with imaging of Atad-3 cellular localization as done with other experiments.

Response:

We thank the reviewer for emphasizing the importance of rigorously validating ATAD-3 oligomerization and excluding alternative explanations for the Ca^{2+} uptake defects. We fully agree that these controls are essential for supporting our mechanistic conclusions.

First, as suggested, we have moved the ATAD-3 oligomerization Western blot and Native PAGE data from the Supplementary Materials to the main figures (Fig. 5e) in the revised manuscript. This change highlights the central role of oligomerization in ATAD-3 function and makes the supporting evidence more readily accessible to readers.

Second, to rule out mislocalization as an alternative explanation for the impaired mitochondrial Ca^{2+} uptake, we examined the subcellular localization of the oligomerization-deficient and disease-associated ATAD-3 mutants. We performed confocal imaging of GFP-tagged ATAD-3(Δ240), ATAD-3(F47L), and ATAD-3(G352E) in animals co-expressing TOMM-20::mKate2, a marker of the mitochondrial outer membrane. All mutant proteins exhibited robust co-localization with TOMM-20::mKate2, comparable to that of WT ATAD-3 (Supplementary Fig. 8b). These results demonstrate that the mutant proteins are correctly targeted to mitochondria and that their loss of function cannot be attributed to defective mitochondrial localization.

Together, these additional experiments confirm that disruption of ATAD-3 oligomerization, rather than altered protein expression or mislocalization, is responsible for the impaired mitochondrial Ca^{2+} uptake.

7) As described in the overall comments section, please examine potential mechanisms by which Atad-3 could be disrupting mitochondrial Ca2+ elevation- e.g. mitochondrial membrane potential, integrity, network.

Response:

We thank the reviewer for this constructive suggestion to explore an alternative mechanism by which ATAD-3 disruption might impair mitochondrial Ca^{2+} elevation. In response, we performed additional experiments to evaluate mitochondrial membrane potential, mitochondrial

network organization, and mitochondrial ultrastructure. These new data have been incorporated into the revised manuscript.

First, we assessed mitochondrial membrane potential (Δψm) using TMRE staining. As described in our response to Comment 1, depletion of ATAD-3 in the epidermis did not significantly alter Δψm under either basal conditions or following wounding (Supplementary Fig. 6c), indicating that impaired mitochondrial Ca^{2+} uptake in *atad-3* mutants is not due to reduced membrane potential or loss of the electrochemical driving force.

Second, we examined mitochondrial network organization using mito::GFP imaging. We observed increased mitochondrial fragmentation in *atad*-3 conditional knockout animals compared with WT controls (Supplementary Fig. 6a). Transmission electron microscopy further demonstrated disrupted mitochondrial network architecture (Supplementary Fig. 6b), consistent with the previously reported role of ATAD-3 in maintaining mitochondrial structure. In addition, epidermal *atad-3* RNAi reduced endogenous ATP-3::GFP and TOMM-20::mKate2 signals (Supplementary Fig. 6d and 6e), suggesting compromised mitochondrial membrane integrity. These observations raised the possibility that altered mitochondrial architecture might indirectly impair Ca^{2+} uptake.

Finally, to determine whether mitochondrial fragmentation alone could account for the observed Ca^{2+} uptake defects, we examined *fzo-1(*mammalian homology of MFN1/2*)* mutants, which exhibit mitochondrial fragmentation and reduced membrane potential (Supplementary Fig. 6f). Despite these defects, *fzo-1* mutants displayed normal wound-induced mitochondrial Ca^{2+} elevation comparable to WT animals (Supplementary Fig. 6g). This finding indicates that mitochondrial fragmentation per se is not sufficient to disrupt mitochondrial Ca^{2+} uptake. Similarly, RNAi-mediated knockdown of *immt-1*, a core component of the mitochondrial inner membrane organizing system, did not affect mitochondrial Ca^{2+} levels under basal or wounded conditions (Supplementary Fig. 6h). These results demonstrate that mitochondrial fragmentation or altered cristae organization alone are not sufficient to block mitochondrial Ca^{2+} uptake.

Taken together, these experiments indicate that ATAD-3-dependent mitochondrial Ca^{2+} uptake is not an indirect consequence of altered mitochondrial membrane potential, network morphology,

or gross mitochondrial integrity. Instead, they support a model in which ATAD-3 plays a more direct regulatory role in mitochondrial Ca^{2+} entry following injury.

Minor comments:

8) I am not familiar with the method used for RNAi screening (e.g. the use of the rde-1 mutant animals). The authors referred to prior publications on this method. Please provide more detail on this in the methods section as this is an important aspect of the paper and may not be familiar to some readers less familiar with the C. elegans model.

Thanks for the suggestion, we have added the references and detailed in the methods section.

9) The authors use a MCU mutant strain with excised exons to produce knockdown. A critical aspect of their research is the assumption that the MCU protein is non-functional. This is critical and should be expanded on either in discussion or with experimental validation. As far as I have seen, there is no validation or references to this validation elsewhere.

Response:

We thank the reviewer for highlighting this important point regarding the functional status of the *mcu-1* mutant used in this study.

The *mcu-1* mutant strain used here was generated by CRISPR-Cas9-mediated genome editing, in which three entire exons (821 bp total) were excised from the *mcu-1* locus, which contains six exons in total. This deletion introduces a frameshift in the coding sequence. On this basis, the allele is predicted to be a null.

Importantly, the functional consequences of this *mcu-1* mutation on mitochondrial Ca^{2+} uptake have been independently validated in multiple prior studies. These works demonstrate that this allele abolishes mitochondrial Ca^{2+} uptake in response to physiological stimuli, supporting its classification as a nonfunctional *mcu-1* mutant (doi: 10.1016/j.devcel.2014.08.002, 10.3390/ijms21228622, and 10.1016/j.redox.2023.102759). We have now explicitly cited these

studies in the revised manuscript and expanded the relevant Discussion section to clarify the validation and functional status of the *mcu-1* mutant.

10) Please review the references to Figure 5 in the text of the article. They do not match with the content of the figure panels.

Corrected.

11) It appears that there are no statistical summaries for the data in Figure 5. Please address this.

Added.

12) Often the figure legends lacked sufficient information describing the statistical methods. Please address this.

We have now added more information in the figure legend.

13) Figure 1: UW vs W (it is not clear what UW and W refer to in the figure or figure legend) please change the description to baseline and wound, or describe the UW and W acronym in the figure legend.

Corrected.

14) Fig 1 Panel B: please provide a colour code scale bar, it is obvious warm colours are greater Ca2+, but this should still be shown.

Thanks for the point. We have now added colour coded scale bar.

15) Scale bar unit missing Figure 5B.

Added.

16) Figure 5A,B: The transmembrane domain is labeled in dark blue in panel A, but in panel B this colour is not present. There is a red label on the predicted 3d protein structure in panel B- I presume that this is supposed to be the transmembrane domain labelled in blue (not red)? Please fix colour coding.

Fixed.

Reviewer #2 (Remarks to the Author):

I co-reviewed this manuscript with one of the reviewers who provided the listed reports. This is part of the Nature Communications initiative to facilitate training in peer review and to provide appropriate recognition for Early Career Researchers who co-review manuscripts.

We thank the reviewer for the helpful suggestions on our manuscript.

Reviewer #3 (Remarks to the Author):

Xu et al. observed that tissue wounding in C. elegans induces mitochondrial calcium signaling independent of the mitochondrial calcium uniporter (MCU). Through an RNAi screen, they identified ATAD3 and MICU3 as key regulators of this pathway. The authors further demonstrated that wounding promotes ATAD3 oligomerization, which is essential for facilitating MCU-independent mitochondrial calcium uptake. The results are interesting but lacking in rigor to really prove that ATAD3 is a calcium uptake channel.

Major comments:

1. In the lab's previously published article (https://doi.org/10.1016/j.devcel.2014.08.002), local mtROS production was observed following laser wounding. In the current manuscript, the authors employed a higher-energy laser for wounding, which could potentially induce greater

mtROS production. This raises concern that excessive mtROS may lead to mitochondrial swelling, thereby affecting the accuracy of mitoGCaMP5 signals.

To address this, it would be helpful for the authors to assess mtROS level and mitochondrial membrane potential following high-energy laser wounding to confirm that mitochondrial function remains intact and that mitoGCaMP5 readings are reliable.

Response:
We thank the reviewer for these thoughtful concerns regarding potential effects of high-energy laser wounding on mitochondrial integrity and the reliability of mito::GCaMP5 signals.

In our previous study (https://doi.org/10.1016/j.devcel.2014.08.002), epidermal wounding was performed using a femtosecond laser, which produces highly confined and deep lesions. In the present study, we used a Micropoint UV laser system (Oxford Instruments, Andor), which allows precise adjustment of laser output to generate reproducible injuries of defined size. Because absolute laser energy values vary across systems and experimental conditions, we now describe injuries operationally as "*small-scale*" (~1 µm in diameter) and "*large-scale*" (~10 µm in diameter) wounds in the revised manuscript. This provides a more consistent and biologically meaningful classification of injury severity.

Second, to determine whether our wounding conditions induce excessive mtROS that could compromise mitochondrial integrity, we measured mitochondrial ROS using MitoSOX staining in epidermal cells following injury. We detected an elevation of mtROS under large-scale wounding conditions (Response Fig. 2a), which is comparable to the previous report. In addition, we did not observe mitochondrial swelling under this wounding context (Fig. 1a and Supplementary Fig. 1c, e-g). These results indicate that our laser settings do not cause pathological mtROS accumulation that might induce mitochondrial swelling or artifactually alter mito::GCaMP5 signals.

Third, we verified subcellular localization of mito::GCaMP5 using independent mitochondrial markers. In mito::GCaMP5 transgenic animals, fluorescence exhibited strong colocalization with TMRM, a mitochondrial membrane potential-dependent dye, as well as Tomm-20::mKate2, a

mitochondrial outer membrane marker, both before and after wounding (Supplementary Fig. 1e and Fig. 1f). No detectable cytosolic or non-mitochondrial GCaMP signal was observed. These results confirm that mito::GCaMP5 is specifically and stably localized to mitochondria under both basal and injury conditions.

Finally, we also validated mitochondrial Ca^{2+} responses using an independent genetically encoded sensor, mito::NEMOer, which exhibited identical response patterns to mito::GCaMP5 in both wild-type and mcu-1 mutant animals (Supplementary Fig. 1g). This cross-validation further confirms the robustness of our mitochondrial Ca^{2+}

Together, these experiments demonstrate that our laser wounding conditions do not induce excessive mtROS or irreversible mitochondrial damage. The localization and functional controls demonstrate that the Ca^{2+} transients reported by mito::GCaMP5 represent bona fide mitochondrial Ca^{2+} uptake. We have included these data (except mitoSOX staining data, which is not highly related to the discovery) and clarification in the revised supplementary Figures and refer to them in the result section.

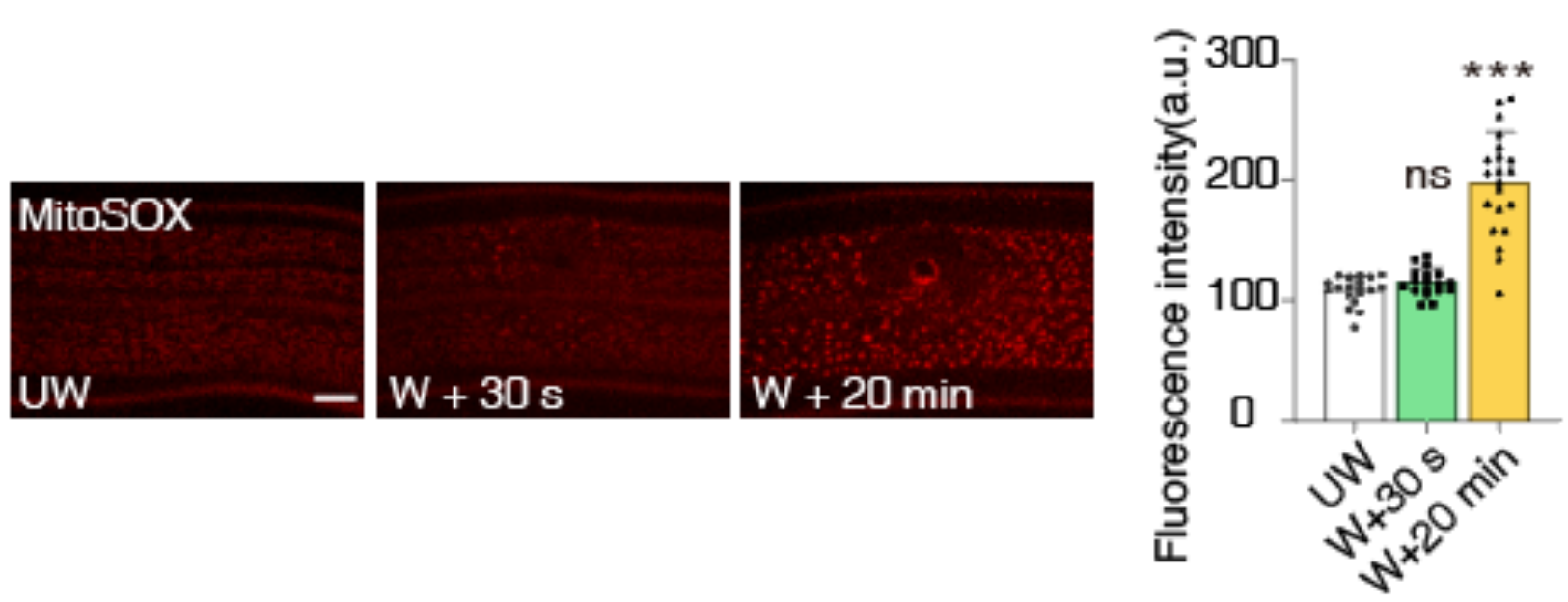

Response Fig. 2

Response Fig. 2 Wounding induces mitochondrial ROS production in epidermal cells. Left: Confocal images of mitoSOX stain in wild-type animals before and indicated time points after wounding. Scale: 10 µm. Right: Quantification of mitoSOX intensity before and after wounding. n ⩾ 13 samples. ns, $P > 0.05$, ***, $P < 0.001$, One-way ANOVA.

2. In Figure 1, the authors utilized three different wounding methods: high-energy laser, needle, and ultrasound-induced wounding. In Figure 2, it appears that both needle and laser wounding were used. However, in the remaining figures, the wounding method is not clearly identified, though it seems laser wounding was predominantly used. First, please clearly indicate the wounding method used in each figure and panel to avoid confusion. Second, for the RNAi screen, the authors used needle wounding, while in most other experiments laser wounding appears to be employed. This inconsistency needs to be addressed. Please either standardize the wounding method across experiments or provide a detailed rationale for using different wounding techniques in different parts of the study.

Response:

We thank the reviewer for this constructive. We agree that explicit identification of the wounding method in each experiment is essential for clarity.

In the revised manuscript, we now clearly indicate the wounding method used in every figure and relevant panel in both figure labels and legends. We have also standardized terminology throughout the text. Laser-based injuries are now described as *small-scale* or *large-scale laser wounding*, while *needle-induced wounding* and *ultrasound-induced wounding* are explicitly labeled where used. These changes remove ambiguity and allow readers to immediately identify the injury paradigm used in each experiment.

We would like to emphasize that all three injury modalities, laser, needle, and ultrasound, robustly trigger mitochondrial Ca^{2+} elevation in WT animals (Fig 1). Thus, mitochondrial Ca^{2+} uptake is a general response to epidermal injury and is not specific to any single wounding technique. The choice of wounding method in each experimental context reflects technical considerations rather than inconsistency:

RNAi screening: Needle wounding was used because it is rapid, simple to perform, and highly reproducible, making it well-suited for higher-throughput screening of large numbers of genetic perturbations.

Live imaging and mechanistic experiments: Laser wounding provides superior spatial precision, temporal control, and reproducibility of wound size and position. This is essential for quantitative time-resolved imaging of mitochondrial Ca^{2+} dynamics and for mechanistic dissection of the uptake pathway. Therefore, laser wounding was used for most subsequent experiments.

Ultrasonic wounding: This method was included to demonstrate that mitochondrial Ca^{2+} uptake is also triggered by mechanical injury independent of direct plasma membrane ablation, further supporting the generality of the phenomenon. In addition, ultrasonic wounding is particularly advantageous for high-throughput assays such as Western blotting, as it enables rapid and relatively uniform wounding of large worm populations.

Thus, the use of different wounding paradigms was a deliberate and complementary strategy balancing throughput, precision, and generalizability. We have now clarified this rationale in the revised manuscript.

3. It is very interesting that the MICU3 mutant increases basal mitochondrial calcium levels, and that this effect can be suppressed by overexpression of MICU2 or MICU3. MICU2 and MICU3 are known to form heterodimers with MICU1 to enhance MCU-dependent mitochondrial calcium uptake. Have the authors examined MICU1 expression levels in the MICU3 mutant group? Additionally, the authors state that MICU3 interacts with MCU-1; however, according to the literature, this interaction is typically mediated through MICU1. It would be important to assess MICU1–MICU3 binding directly and to determine how this interaction influences MCU-1 under both baseline and wounding conditions.

Response:

We thank the reviewer for this insightful comment regarding the relationship between MICU-1, MICU-3, and MCU-1. In response, we performed additional genetic and imaging analyses to clarify their interactions and functional hierarchy.

First, to determine whether MICU-1 and MICU-3 influence each other's expression levels, we quantified MICU-1::GFP fluorescence in *micu-3* mutants (Figure S4l) and MICU-3::GFP

fluorescence in *micu-1* knockdown animals (Figure S4m). In both cases, no significant difference was observed compared to respective controls, indicating that neither protein measurably regulates the abundance of the other at the expression level.

Second, we attempted to test a physical interaction between MICU-1 and MICU-3. Co-immunoprecipitation using the endogenous MICU-1::GFP knock-in strain was technically limited by the low abundance of the fusion protein, precluding robust biochemical detection. We therefore adopted an alternative tagging strategy by co-expressing MICU-3::GFP with overexpressed MICU-1::mKate2. However, MICU-1 overexpression resulted in partial mislocalization (lysosome, Response Fig. 3a), preventing reliable co-immunoprecipitation analysis. Although direct biochemical evidence of binding remains technically challenging in our *in vivo* system, live imaging revealed co-localization of MICU-1::GFP and MICU-3::mKate2 signals within mitochondria (Response Fig. 3b, Figure S4j and S4k), supporting their presence within the same subcellular compartment.

Importantly, multiple studies in mammalian systems have demonstrated that MICU3 forms heterodimers with MICU1, rather than interacting directly with MCU independently of MICU1 (Mol Cell 2014; EMBO Rep 2013; eLife 2020; and Cell Death Differ 2018 etc.,). In addition, conserved cysteine residues implicated in MICU1 dimerization (e.g., MICU1 Cys465, MICU2 Cys410, and MICU3 Cys515) and conserved hydrophobic interfaces suggest that the structural basis for MICU1–MICU3 interaction is evolutionarily preserved. Together, these data support the likelihood that MICU-1 and MICU-3 interact in *C. elegans* as well, consistent with established models in other organisms.

Third, to define their functional relationship, we performed genetic interaction analyses. Under basal conditions, *micu-1* mutants show a normal mito::GCaMP5 signal compared to WT and similar to *mcu-1* mutants. In contrast, *micu-3* mutants showed a dramatic elevation in basal mito::GCaMP5 signal. Importantly, both *micu-1; micu-3 and mcu-1;micu-3* double mutants restored the mito::GCaMP5 to baseline levels (Figure 2i and 2j). These results indicate that elevated mitochondrial Ca^{2+} observed in *micu-3* mutants requires both MICU-1 and MCU-1, placing *micu-3* genetically upstream of the MICU-1-MCU-1 axis and suggesting that MICU-3 negatively regulates MCU complex activity (Figure 2k). After wounding, *micu-1* mutants failed

to exhibit robust and dramatic mitochondrial Ca^{2+} uptake (Supplementary Figure 4f-4h), further supporting the conclusion that MICU-1 is required for MCU-1-dependent Ca^{2+} influx after wounding.

Together, our genetic and imaging analyses support a model in which MICU-1 and MICU-3 function as a regulatory module controlling MCU channel activity. MICU-1 is required for channel activation, whereas MICU-3 acts as a restraining factor that limits MCU-dependent Ca^{2+} entry, thereby defining the basal threshold and dynamic range of mitochondrial Ca^{2+} uptake under both resting and injury conditions.

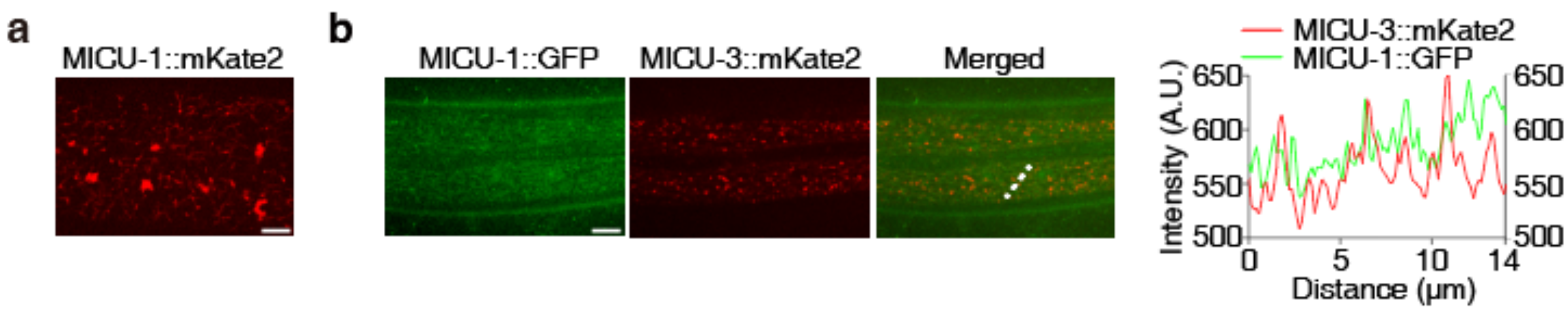

Response Fig. 3 MICU-1 and MICU-3 expression in epidermal cells. (a) Representative image of epidermal cells overexpressing MICU-1::mKate2 alone. Scale: 10 μm. (b) Co-expression of MICU-1::GFP (green) and MICU-3::mKate2 (red); merged image shows extensive colocalization. Scale: 10 μm.

4. While the authors clearly demonstrate a role for ATAD3 in MCU-independent mitochondrial calcium uptake, previous studies have already shown that ATAD3 interacts with SIRT3 to regulate MAM dynamics, which in turn influence mitochondrial calcium homeostasis independently of MCU (DOI: 10.7150/ijbs.89253). These studies also indicate that ATAD3 oligomerization is disrupted by acetylation, and that ATAD3 monomer interacts with the IP3R1–GRP75–VDAC1 complex. Given these findings, the authors should examine SIRT3 levels and acetylation status in their wounding model to determine whether increased acetylation disrupts ATAD3 oligomerization, thereby preventing mitochondrial calcium uptake.

Response:

We thank the reviewer for highlighting prior work linking ATAD3 acetylation and SIRT3-dependent regulation of mitochondria-associated membrane (MAM) dynamics to mitochondrial Ca^{2+} homeostasis (DOI: 10.7150/ijbs.89253). We carefully considered whether a similar regulatory mechanism operates in our *C. elegans* injury model and performed additional analyses to address this possibility.

A direct mitochondrial SIRT3 ortholog is not present in *C. elegans*. The closest sirtuin homologs, *sir-2.2* and *sir-2.3*, are more closely related to mammalian SIRT4 than to SIRT3. To test whether these sirtuins participate in ATAD-3-dependent mitochondrial Ca^{2+} uptake, we depleted *sir-2.2* and *sir-2.3* by RNAi and examined wound-induced mitochondrial Ca^{2+} dynamics. In both cases, mito::GCaMP5 responses before and after wounding were indistinguishable from controls (Response Fig. 4a-c), indicating that sirtuin activity is not required for ATAD-3-mediated Ca^{2+} uptake after injury in our experimental system.

In mammalian cells, ATAD3A acetylation at lysine 135 has been reported to disrupt oligomerization (DOI: 10.7150/ijbs.89253). Surprisingly, sequence alignment revealed that this lysine is not conserved in *C. elegans* ATAD-3 (Response Fig. 4d). We identified the nearest lysine residue in the corresponding region and generated a potential non-acetylatable mutant (K136A). This mutant displayed normal oligomerization (Response Fig. 4h) and did not affect injury-induced mitochondrial Ca^{2+} uptake (Response Fig. 4e-g), indicating that acetylation at this position does not regulate ATAD-3 function in *C. elegans.* However, we are not able to rule out other acetylation possibility that may affect ATAD-3 oligomerization that further impact on mitochondrial Ca^{2+} uptake, which will be an important direction for future research.

Together, follow the guidance of reviewer's comments, we tested the mechanism of acetylation on ATAD-3 oligomerization. However, our analyses suggest that the SIRT3–ATAD3 acetylation-dependent mechanism described in mammalian systems is not evolutionarily conserved in *C. elegans*, and does not underlie ATAD-3-dependent mitochondrial Ca^{2+} uptake in our injury model.

Because all experimental tests of this hypothesis yielded negative results and do not alter the main conclusions of the study, we have not incorporated these data into the revised manuscript.

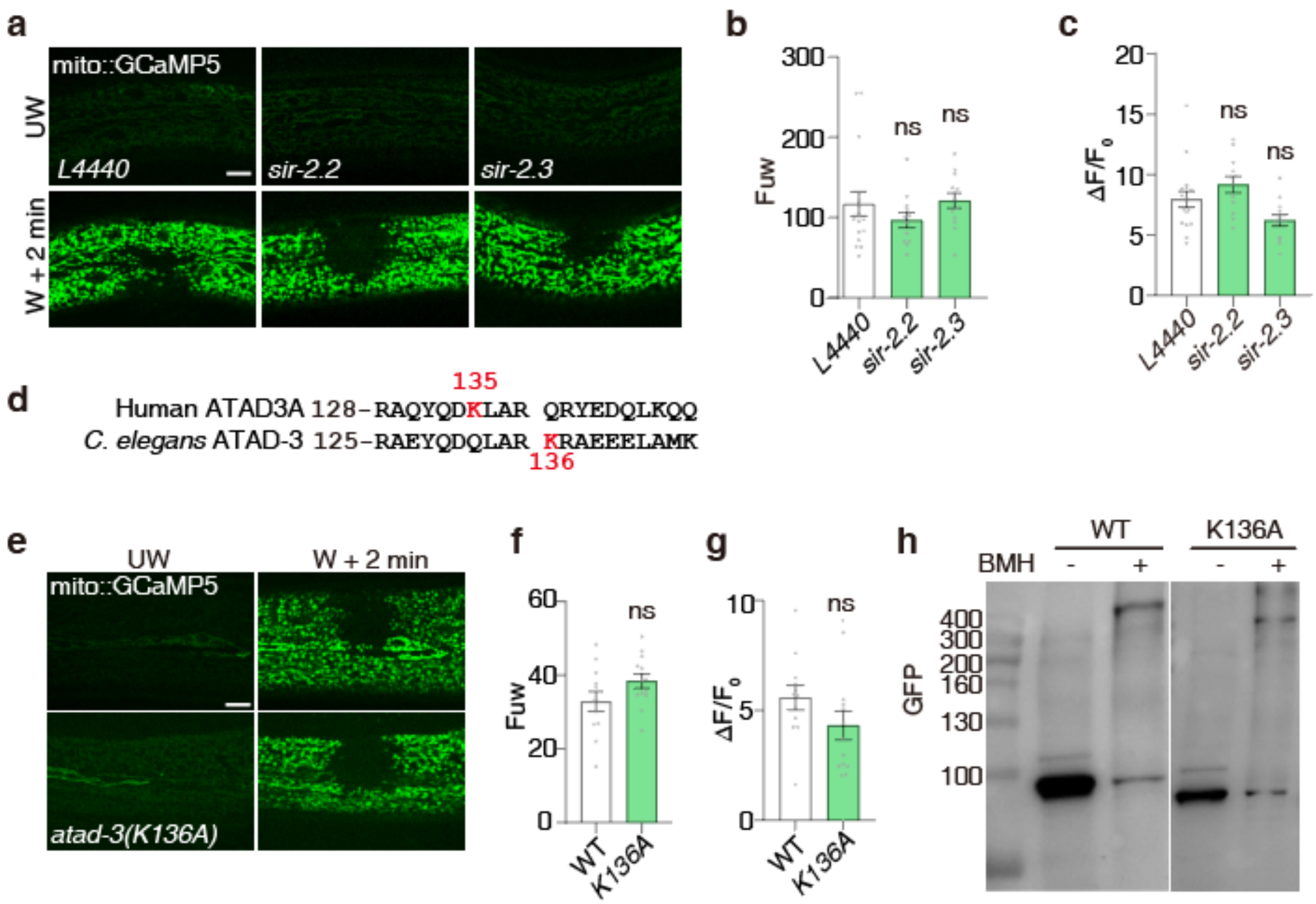

Response Fig. 4

Response Fig. 4 Acetylation is not required for ATAD-3 oligomerization or wounding-induced mitochondrial Ca^{2+} uptake. (a-c) RNAi knockdown of *sir-2.2* and *sir-2.3* does not affect mitochondrial Ca^{2+} levels before and after wounding, as measured by mito::GCaMP5 intensity in epidermal cells. Scale: 10 μm. n ⩾ 12 samples. ns, $P > 0.05$, One-way ANOVA. (d) Sequence alignment of human ATAD3A and *C. elegans* ATAD-3, highlighting the region surrounding lysine 136, corresponding to the reported acetylation site in mammals. (e-g) Epidermal overexpression of *atad-3(K136A)* does not alter basal or wounding-induced mitochondrial Ca^{2+} uptake compared with WT. Scale: 10 μm. n ⩾ 12 samples. ns, $P > 0.05$, Student's t-test. (h) ATAD-3(K136A) shows oligomerization comparable to WT, as assessed by SDS-PAGE analysis.

5. What is the phenotype of atad-3 knockdown (loxP) in the epidermal cells on mitochondrial viability and tissue viability over a time course? The concern is the interpretation that atad-3 RNAi or the loxP-KO is otherwise compromising mitochondria structure-function and causing cellular damage, given its known role in regulating inner and outer mitochondrial membrane apposition and greater effect this would have on all mitochondrial processes, hence leading to a misinterpretation that atad3 is a calcium channel.

Does epidermal deletion of atad3 reduce mitochondrial respiration, or other functions? Somehow the authors need to rule out the very simple interpretation that atad-3 loss compromises mitochondria structure-function more globally so that they cannot take up calcium given other major structural defects (as they authors state, germline deletion of atad3 is lethal).

As an aside, what about deleting components of the MICOS complex in epidermal cells, especially MIC60, which also directly regulates inner and outer mito membrane coordination and structural alignment? Would loss of MICOS complex also lead to loss of wounding induced calcium entry? Deletion of genes that result in the loss of the MICOS complex is also known to be lethal. Mitochondrial permeability transition that also lets in calcium, or lets it out, requires correct apposition of the inner and outer mito membranes.

Response:

We thank the reviewer for this thoughtful and important comments. We fully agree that ATAD-3 has established roles in maintaining inner-outer mitochondrial membrane apposition and cristae architecture. Therefore, it is critical to exclude the possibility that impaired mitochondrial Ca^{2+} uptake observed in *atad-3*-deficient epidermal cells arises from global mitochondrial structural collapse or generalized dysfunction rather than from a specific regulatory role in Ca^{2+} entry. To address these, we performed additional analyses to examine mitochondrial viability, tissue integrity, and the contribution of general mitochondrial structural defects.

First, because germline deletion of *atad-3* is lethal, we employed an epidermal-specific conditional knockout (cKO) to assess post-developmental phenotypes. Epidermal *atad-3* cKO remained viable, displayed normal morphology and locomotion, and showed no progressive degeneration of the epidermis from young adult to Day 5 of adulthood (Response Fig. 5a).

Quantification of mitochondrial marker mito::GFP intensity revealed no significant reduction in mitochondrial abundance (Response Fig. 5b and 5c). Importantly, epidermal wound closure kinetics was comparable to WT, indicating preserved tissue repair capacity (Response Fig. 1b). Thus, within the time frame of our Ca^{2+} imaging experiments, epidermal deletion of *atad-3* does not cause overt tissue collapse or loss of cellular viability.

Second, although mitochondria in *atad-3* cKO epidermal cells exhibit fragmentation and partial cristae disorganization, as revealed by mito::GFP imaging and transmission electron microscopy (Supplementary Fig. 6a-b), they retain clearly defined inner and outer membranes and do not display swelling, rupture, or organelle loss. Importantly, TMRE staining shows that mitochondrial membrane potential ($\Delta\psi m$) is comparable between control and *atad-3*-deficient epidermal cells under basal conditions and decreases similarly following wounding (Supplementary Fig. 6c). These results indicate that mitochondria remain polarized and metabolically competent rather than globally depolarized.

Consistent with preserved respiratory activity, wound-induced mitochondrial ROS production in maintained in *atad-3* cKO animals (Response Fig. 1a), arguing against major respiratory chain failure or oxidative collapse.

Third, to test whether loss of inner-outer membrane coordination per se is sufficient to block injury-induced Ca^{2+} uptake, we disrupted the MICOS complex by epidermal knockdown of *immt-1* (MIC60), a core MICOS component. As expected, *immt-1* depletion caused marked mitochondrial fragmentation (Supplementary Fig. 6h). Nevertheless, mito::GCaMP5 imaging showed normal wound-induced mitochondrial Ca^{2+} uptake in these animals (Supplementary Fig. 6h). Thus, disruption of inner-outer membrane alignment and cristae architecture alone is not sufficient to eliminate mitochondrial Ca^{2+} entry.

We further examined mitochondrial Ca^{2+} dynamics in *fzo-1* (MFN1/2) mutants, which exhibit pronounced mitochondrial fragmentation and reduced mitochondrial membrane potential (Supplementary Fig. 6f). Despite these defects, *fzo-1* mutants displayed elevated mito::GCaMP5 signals both before and after wounding (Supplementary Fig. 6g), indicating that mitochondrial fragmentation alone is not sufficient to block mitochondrial Ca^{2+} uptake.

Collectively, these data demonstrate that: Epidermal *atad-3* deletion does not cause loss of tissue viability. Mitochondria remain present, polarized, and structurally intact despite moderate architectural defects. Comparable or stronger disruptions of mitochondrial architecture (MICOS depletion) do not block wound-induced Ca^{2+} uptake. Therefore, the loss of mitochondrial Ca^{2+} entry in *atad-3*-deficient epidermal cells cannot be explained by global mitochondrial collapse or nonspecific structural dysfunction. Instead, these findings support a specific regulatory role for ATAD-3 in injury-induced mitochondrial Ca^{2+} uptake rather than a secondary consequence of generalized mitochondrial failure.

We have incorporated these clarifications into the revised manuscript.

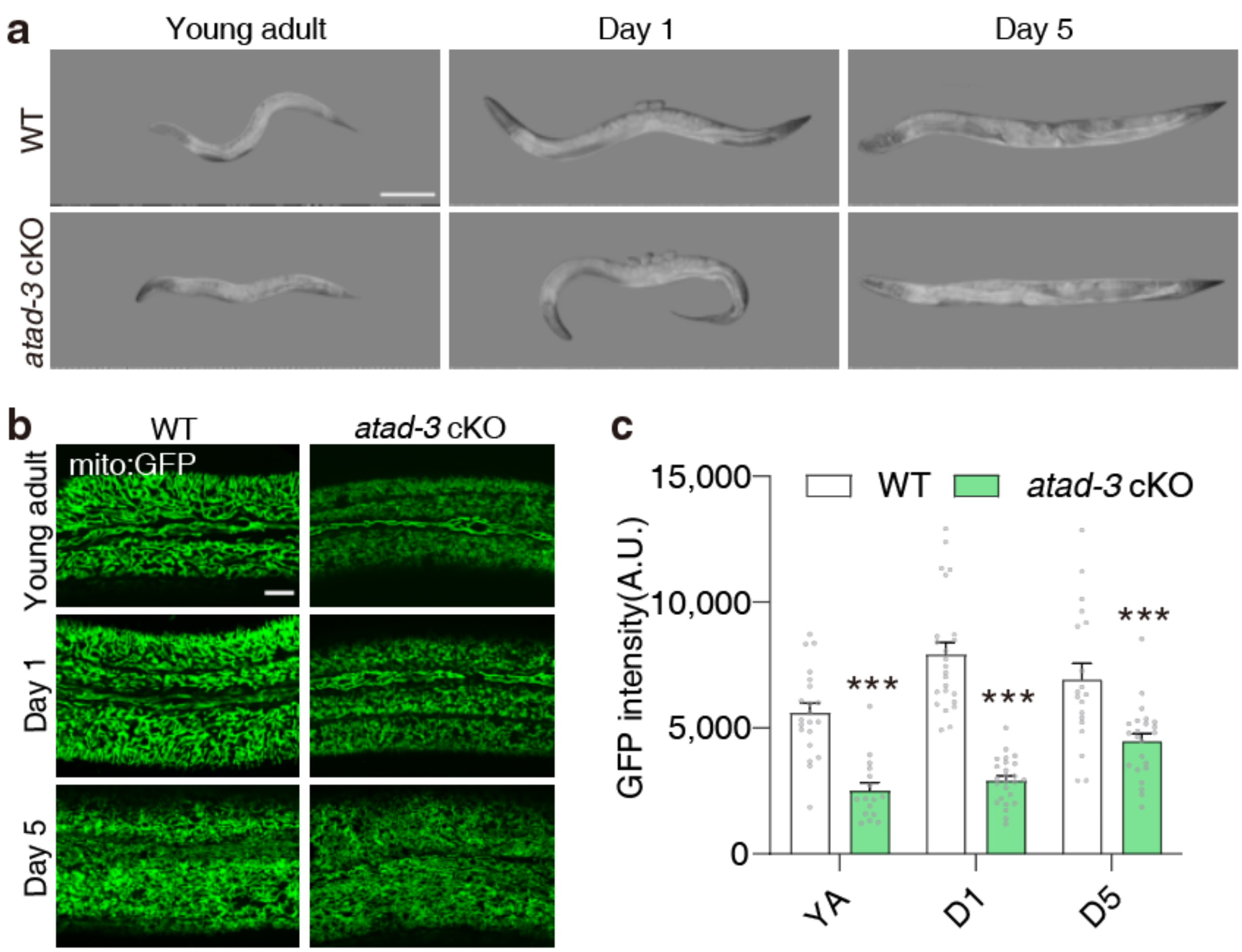

Response Fig. 5 Epidermal *atad-3* cKO does not compromise overall tissue viability. (a) Representative DIC images of WT and *atad-3* cKO animals at the young adult, Day 1, and Day 5 stages. Scale: 200 μm. (b) Representative images of epidermal mitochondrial morphology

visualized by mito::GFP in WT and *atad-3* cKO animals. Scale: 10 μm. c, Quantitation of mito::GFP intensity shown in Panel (b) n ⩾ 15 samples. ns, $P > 0.05$, Two-way ANOVA.

6. There is a complete absence of data showing the architectural or structural effects of the atad3 knockdown or epidermal KO at the level of the mitochondria (electron microscopy). This is even more important to show for all the mutants in atad3 that were used, some of which prevented oligomerization and the calcium defect, some of which did not.

Does deletion of this gene or some of the mutants correlate with a more general loss of mitochondrial cristae organization and the proper stacking of the inner and outer mito membranes?
Does it correlate with mitochondrial respiration and function?
How about blue native gels to show the respiratory complexes are in tact?

Response:
We thank the reviewer for this important and constructive comment. We agree that, given the known structural roles of ATAD-3 in mitochondrial membrane organization, it is essential to determine whether the Ca^{2+} uptake defect observed in *atad-3*-deficient animals might result indirectly from broader mitochondrial architectural or respiratory dysfunction. We have therefore performed additional structural and functional analyses, as detailed below. Please see also in our response to comments 5.

First, we performed transmission electron microscopy (TEM) on epidermal cells from *atad-3* epidermal-specific knockout animals. Loss of *atad-3* resulted in increased mitochondrial fragmentation and partial reduction in cristae density, consistent with previously reported architectural roles of ATAD-3 (Supplementary Fig. 6b). However, mitochondria retained clearly defined inner and outer membranes and intact boundary membranes. We did not observe widespread mitochondrial swelling, rupture, or collapse of membrane integrity (Supplementary Fig. 6b). Thus, ATAD-3 contributes to cristae organization, but its loss does not cause catastrophic mitochondrial destruction that would trivially abolish Ca^{2+} uptake.

Second, we assessed mitochondrial membrane potential (Δψm) using TMRE staining and found no significant difference between WT and *atad-3*-deficient epidermal cells under basal conditions (Supplementary Fig. 6c). Both genotypes exhibited comparable partial depolarization after wounding (Supplementary Fig. 6c). These data indicate that the electrochemical driving force remains intact in *atad-3*-deficient mitochondria. In addition, mitochondrial ROS measurements after wounding revealed similar elevation in *atad-3*–deficient epidermis relative to WT (Response Fig. 1a), arguing against major respiratory chain failure or oxidative collapse.

Third, we agree that blue-native PAGE provides valuable information on respiratory chain integrity. We therefore performed blue-native PAGE on whole-animal mitochondrial extracts from epidermal *atad-3* knockout animals. The abundance and assembly patterns of respiratory complexes were comparable to WT (Response Fig. 6), indicating that global respiratory chain organization is largely preserved. While whole-animal preparations may mask subtle epidermis-specific differences, these data nonetheless demonstrate that ATAD-3 loss does not cause wholesale disruption of respiratory complex assembly. Moreover, we examined endogenous ATP-3::GFP and TOMM-20::mKate2 levels in epidermal cells and observed modest reductions in signal intensity, consistent with partial alterations in mitochondrial protein homeostasis, but not complete loss of mitochondrial mass or identity (Supplementary Fig. 6d and 6e).

Finally, to test whether generalized loss of inner–outer membrane coordination is sufficient to block wound-induced Ca^{2+} uptake, we disrupted the MICOS complex by epidermal knockdown of *immt-1* (MIC60), a core cristae-organizing factor. As expected, *immt-1* depletion caused pronounced cristae disorganization and mitochondrial fragmentation. However, these animals displayed normal wound-induced mitochondrial Ca^{2+} uptake (Supplementary Fig. 6h). Similarly, *fzo-1* mutants with severe mitochondrial fragmentation retained robust Ca^{2+} entry (Supplementary Fig. 6g). Therefore, architectural disruption alone is not sufficient to eliminate mitochondrial Ca^{2+} uptake.

Taken together, our new structural and functional analyses show that:

1. *atad-3* loss causes moderate cristae and network defects, but not catastrophic mitochondrial destruction.

2. Similar or stronger architectural disruptions (e.g., MICOS or *fzo*-1 perturbation) do not abolish Ca^{2+} uptake.
3. Membrane potential and respiratory competence remain largely intact.

Therefore, the impaired mitochondrial Ca^{2+} uptake in *atad-3*-deficient animals cannot be explained by a nonspecific collapse of mitochondrial structure or respiration, supporting our conclusion that ATAD-3 plays a regulatory role in injury-induced mitochondrial Ca^{2+} entry. We have incorporated these clarifications into the revised manuscript and response letter.

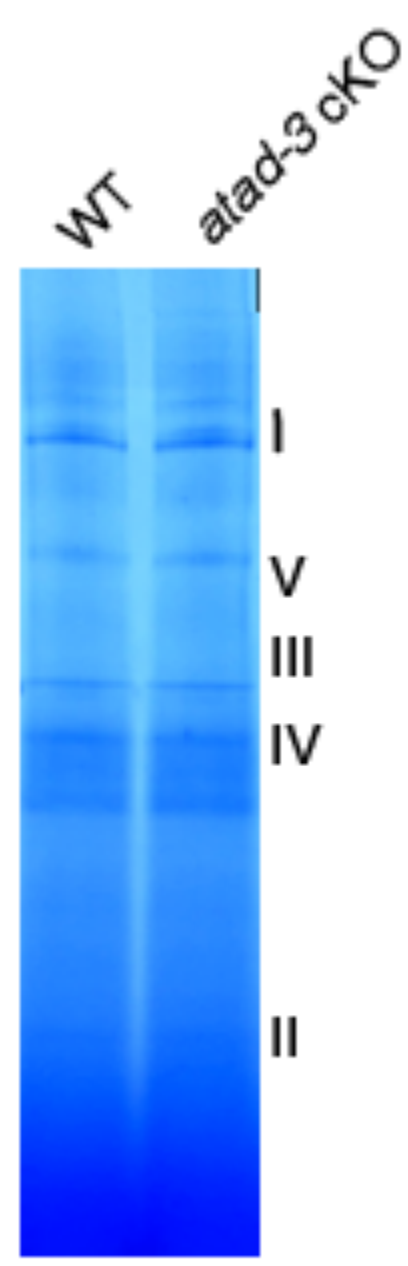

Response Fig. 6

Response Fig. 6 Respiratory chain complex integrity is preserved in *atad-3* epidermal cKO animals. Blue native PAGE analysis of mitochondrial respiratory chain complexes isolated from wild-type and *atad-3* epidermal cKO animals. Major oxidative phosphorylation complexes (Complexes I–V) are detected at comparable levels and assembly states in both genotypes, indicating that loss of ATAD-3 in the epidermis does not cause gross disruption of respiratory chain complex integrity.

Minor comments:

1. Please define MCU-1 when it was first described in the main context.

Added.

2. In Figure 1 and S1, the authors mentioned low-energy and high-energy laser wounding. However, the precise description to distinguish between two procedures was missing in the methods. Was the duration, intensity, or other indexes being different?

Thanks for the suggestions. We have now added the detailed information in the methods section and also provided differences in the main text.

3. Please correct the typo of Figure legend 1G: should be 15 min not 5min.

Corrected

4. For the surface plots shown in Fig. 1F and Fig. S1E, the authors presented three time points: 5 s, 30 s, and 120 s post-wounding. It would be beneficial to combine these panels into a single composite figure or time-course plot to better visualize the dynamic changes and trends in mitochondrial calcium patterns across different groups following wounding.

Thanks for the suggestion, we have now corrected the following suggestions.

5. Please correct the figure legend for Fig. 2C-D, 4C: the panel doesn't match the description in the figure legend.

Corrected.

We sincerely thank all the reviewers for their careful evaluation and for bringing these important issues to our attention. We are particularly grateful for the insightful feedback, which has

significantly improved the quality of this study. We believe that the revisions made in response to their comments have substantially strengthened the rigor and clarity of the manuscript.

REVIEWER COMMENTS (our point-by-point responses are included in blue text)

Reviewer #1 (Remarks to the Author):

Overall, the authors addressed a number of the issues noted at the first review. However, there are still some aspects of the study which require more clarification. Please the comments below.

Response: We thank the reviewer for the continued, thoughtful assessment of our work. We appreciate the recognition that most concerns from the first round were satisfactorily addressed, and we have now made every effort to resolve the remaining issues identified in the second review.

Comments:

1) ATAD-3 disruption reduces wound induced mitochondrial Ca2+ uptake, but the recent results show that there is no impact on mitochondrial membrane potential, mitochondrial ROS, and wound repair dynamics. This is surprising given the known link between mitochondrial Ca2+ uptake and mito membrane depolarization. It is also surprising considering that the new data in the revision suggests that ATAD3 affects the early MCU-dependent Ca2+ uptake, which was shown to impact mito ROS and wound healing in a prior publication. Can you comment on this? The lack of impact of ATAD3 on ROS generation and wound healing are relevant to the impact of the paper, as they are physiological readouts expected to be impacted by mitochondrial Ca2+. This data suggests that Atad3 may impact mitochondrial Ca2+ uptake during wound responses, but that this process may have other roles aside from regulating metabolism. It would be helpful for this data to be presented and discussed in this paper in order to understand the physiological relevance of Atad3.

Response:

We thank the reviewer for raising this important question. The observation that ATAD-3 disruption reduces wound-induced mitochondrial Ca^{2+} uptake yet leaves mitochondrial ROS production and wound repair intact is an interesting and mechanistically informative finding. We have now incorporated these data into the Results and substantially revised the Discussion to

present a mechanistic framework that we believe deepens the biological interpretation of the entire study.

To understand why *atad-3* and *mcu-1* loss-of-function conditions yield phenotypically divergent outcomes despite both reducing wound-induced mitochondrial Ca^{2+} uptake, it is essential to consider the cellular context in which the Ca^{2+} deficit arises in each case. Loss of MCU-1 leaves mitochondrial mass, morphology, membrane potential, and cristae architecture entirely intact under unwounded conditions; the only demonstrable defect is the failure to mount an acute, transient Ca^{2+} uptake response in the first ~ 1 minutes following injury. This precisely defined, temporally bounded signaling failure is sufficient to reduce mitochondrial ROS production and impair downstream wound repair, establishing that the acute Ca^{2+} pulse itself is the direct trigger for mitochondrial ROS generation. By contrast, *atad-3* knockdown or conditional knockout produces an obviously different cellular state: reduced mitochondrial mass (as assessed by mito::GFP signal), fragmented tubular morphology, and altered cristae ultrastructure (Supp. Fig. 6). These are hallmarks of chronic, developmental mitochondrial dysfunction that precede wounding by hours to days. Crucially, MCU-1 remains fully intact and normally localized in *atad-3*-depleted animals. The Ca^{2+} uptake deficiency in these animals, therefore, arises from pre-existing impairment of the mitochondrial compartment as a whole, rather than from the loss of a specific acute signal-transduction event.

A quantitative consideration further clarifies this distinction. ATAD-3 disruption attenuates, rather than abolishes, wound-induced mitochondrial Ca^{2+} uptake, reducing it by approximately 40%-60% relative to wild type (Fig. 2), whereas *mcu-1* mutants exhibit near-complete suppression of the initial uptake peak (Fig. 2, and doi: 10.1016/j.devcel.2014.08.002). We propose that the Ca^{2+}-to-ROS coupling operates above a threshold: residual Ca^{2+} entry in *atad-3*-depleted animals likely sustains the matrix Ca^{2+} concentration required to activate Ca2+-sensitive dehydrogenases or other signals and drive ROS production, whereas the near-complete loss of Ca^{2+} uptake in *mcu-1* mutant falls below this threshold. This interpretation is consistent with the well-established nonlinear relationship between matrix Ca^{2+} concentration and mitochondrial ROS output, in which large reductions below a critical concentration cause disproportionate losses in ROS production (doi: 10.1152/ajpcell.00139.2004; 10.1016/j.bbabio.2009.01.005; 10.1016/j.redox.2015.08.010).

Beyond the quantitative threshold argument, our data also raise the mechanistically interesting possibility that ATAD-3-mediated and MCU-1-mediated Ca^{2+} entry are functionally non-equivalent even at equivalent bulk matrix Ca^{2+} concentrations. ATAD-3 and MCU-1 occupy physically distinct domains of the inner mitochondrial membrane: MCU-1 is distributed throughout the inner membrane, whereas ATAD-3 is enriched at inner membrane-outer membrane contact sites (as suggested by its known interaction with VDAC and IP_3R at mitochondria-ER contact sites in mammals). Localized Ca^{2+} microdomains generated at these contact sites may preferentially engage structural remodeling, lipid metabolism, or mito-nuclear signaling programs rather than the bulk ROS production. The functional non-equivalence of these two uptake routes is itself an important finding and is discussed in the revised manuscript.

Together, our results reveal that mitochondrial Ca^{2+} uptake pathways are functionally heterogeneous and non-interchangeable, a conceptually important insight that challenges the prevailing view of mitochondrial Ca^{2+} uptake as a monolithic process. The identification of an MCU-independent uptake pathway that operates in a distinct cellular context, is activated by a specific molecular trigger (oligomerization rather than constitutive membrane potential), and connects to disease-relevant protein variants (ATAD3A mutations) constitutes a significant advance. The fact that this pathway does not duplicate MCU-1's downstream outputs is precisely what makes it a novel and distinct component of the mitochondrial Ca^{2+} signaling repertoire. We have restructured the Discussion to make this argument explicit and forcefully, and we hope the revised framing will be convincing.

The following text has been added to the Discussion:

"The absence of detectable ROS or wound repair defects in *atad-3*-depleted animals, despite reduced wound-induced Ca^{2+} uptake, is best understood by contrasting the nature of the Ca2+ deficit in *atad-3* versus *mcu-1* loss-of-function contexts. In mcu-1 mutants, acute Ca^{2+} signaling is specifically abolished while mitochondrial structure remains intact, and this alone is sufficient to impair ROS production and wound repair. By contrast, *atad-3* epidermal-specific depletion causes chronic structural mitochondrial dysfunction, while MCU-1 remains intact. Residual Ca^{2+} entry likely sustains the threshold matrix Ca^{2+} concentration required for mitochondrial ROS production. Furthermore, ATAD-3-mediated and MCU-1-mediated $Ca2^{+}$ entry may engage distinct downstream effector programs, consistent with their different submitochondrial locations

and structural, rather than bioenergetic, phenotypes associated with *atad-3* loss. Together, these observations support a model in which distinct mitochondrial Ca^{2+} uptake pathways are functionally non-interchangeable and couple cellular injury to different mitochondrial outputs depending on the molecular identity and context of the uptake route."

2) The new results showing Atad3 accumulation in isolated mitochondria is helpful data. In some ways, it is more relevant and easier to understand than the whole-worm Atad3 measurements post-wounding. It is still unclear to me how the whole-worm Atad3 protein abundance increases following 5 minutes of wound induction. It is also unclear to me how the authors relate an increase in whole-worm Atad3 to the greater mitochondrial Atad3 accumulation/oligomerization. Can you provide more clarity on this and revise accordingly?

Response:

We thank the reviewer for this perceptive and constructive critique. We agree that the whole-worm ATAD-3 abundance data require more careful interpretation, and we have revised the manuscript accordingly. The mitochondrial fractionation data are now foregrounded as the primary and most mechanistically interpretable evidence for injury-induced ATAD-3 accumulation and oligomerization. In contrast, the whole-worm western blot data are repositioned as supporting context rather than as an independent mechanistic conclusion.

Regarding the apparent increase in whole-worm ATAD-3 protein levels within 5 minutes of wounding: a genuine increase in total protein abundance on this timescale is extremely interesting and might not be attributed to de novo synthesis. We interpret this observation as most likely reflecting injury-induced changes in ATAD-3 biochemistry and subcellular distribution rather than an increase in total protein mass. We propose the following, non-mutually exclusive mechanisms:

1) Improved recovery due to oligomerization-driven membrane integration. Before wounding, a fraction of ATAD-3 likely exists in a loosely membrane-associated or peripherally mitochondrial pool that is vulnerable to loss during standard whole-worm lysis, either through co-sedimentation with insoluble membrane debris or proteolytic degradation by proteases released upon homogenization. Wounding triggers rapid ATAD-3 oligomerization and stable inner mitochondrial membrane association, as directly demonstrated by BMH crosslinking and

mitochondrial fractionation data. This biochemical transition renders ATAD-3 more resistant to lysis-associated degradation and improves recovery from whole-worm lysates under SDS denaturing conditions. Thus, the apparent increase in whole-worm immunoblot signal following wounding therefore most likely reflects improved protein recovery from the reorganized, membrane-integrated pool rather than an increase in total ATAD-3 abundance.

2) Post-translational stabilization. Injury may trigger rapid post-translational modifications, including phosphorylation, ubiquitination, or SUMOylation, could transiently protect ATAD-3 from constitutive proteasomal or mitochondrial protease-mediated turnover, producing a short-term increase in steady-state protein levels within a narrow post-wounding window. This mechanism has precedent for other stress-responsive mitochondrial proteins (doi: 10.1155/2012/931215, 10.18632/aging.202629, and 10.3390/ijms21165657).

We wish to emphasize that these explanations are fully consistent with and mechanistically complementary to the fractionation data, which provide direct, compartment-resolved evidence that ATAD-3 accumulates in the mitochondrial fraction and forms higher-order oligomeric complexes following injury. The whole-worm blot reflects the same biochemical transition observed at lower resolution. We have now updated the Results and Discussion sections to clearly distinguish between the two levels of evidence and prevent overinterpretation of the whole-worm measurements.

3) The authors provide new data showing that the subcellular localization of ATAD3 in oligomerization deficient ATAD-3 mutants is not significantly altered in unwounded conditions. However, it would also be useful to see whether the localization/clustering of ATAD3 on mitochondria during wound induction is affected in these oligomerization deficient mutants.

Response:
We thank the reviewer for this important suggestion. We have now directly addressed this question by examining ATAD-3 mitochondrial clustering dynamics during wound induction in oligomerization-deficient and disease-associated mutants.
To this end, we performed HiS-SIM live imaging of WT ATAD-3::GFP, the oligomerization-deficient mutant ATAD-3(Δ240)::GFP co-expressed with mitochondrial matrix marker mito::mKate2. Animals were imaged before and 5 minutes after needle wounding.

In WT animals, wounding triggers a rapid and pronounced increase in ATAD-3::GFP clustering on mitochondria, manifesting as discrete, high-intensity foci on the mitochondrial surface that were distinct from the even mitochondrial distribution observed in unwounded animals. In contrast, ATAD-3(Δ240)::GFP failed to undergo injury-induced mitochondrial clustering, despite normal baseline co-localization with mito::mKate2 (Response Fig. 1). The intra-mitochondrial intensity distribution of these mutants remains uniform and indistinguishable from their pre-wound pattern following wounding. As these data were acquired by HiS-SIM images, which preclude robust quantitative analysis, we present representative images to illustrate the qualitative difference.

These results establish a clear functional dissociation: the coiled-coil domain of ATAD-3 is dispensable for constitutive mitochondrial targeting but required for injury-induced mitochondrial reorganization. This suggests that ATAD-3 oligomerization might be the key mechanistic step that gates Ca^{2+} entry. These findings are now incorporated into the Results section in Supp. Fig. 8.

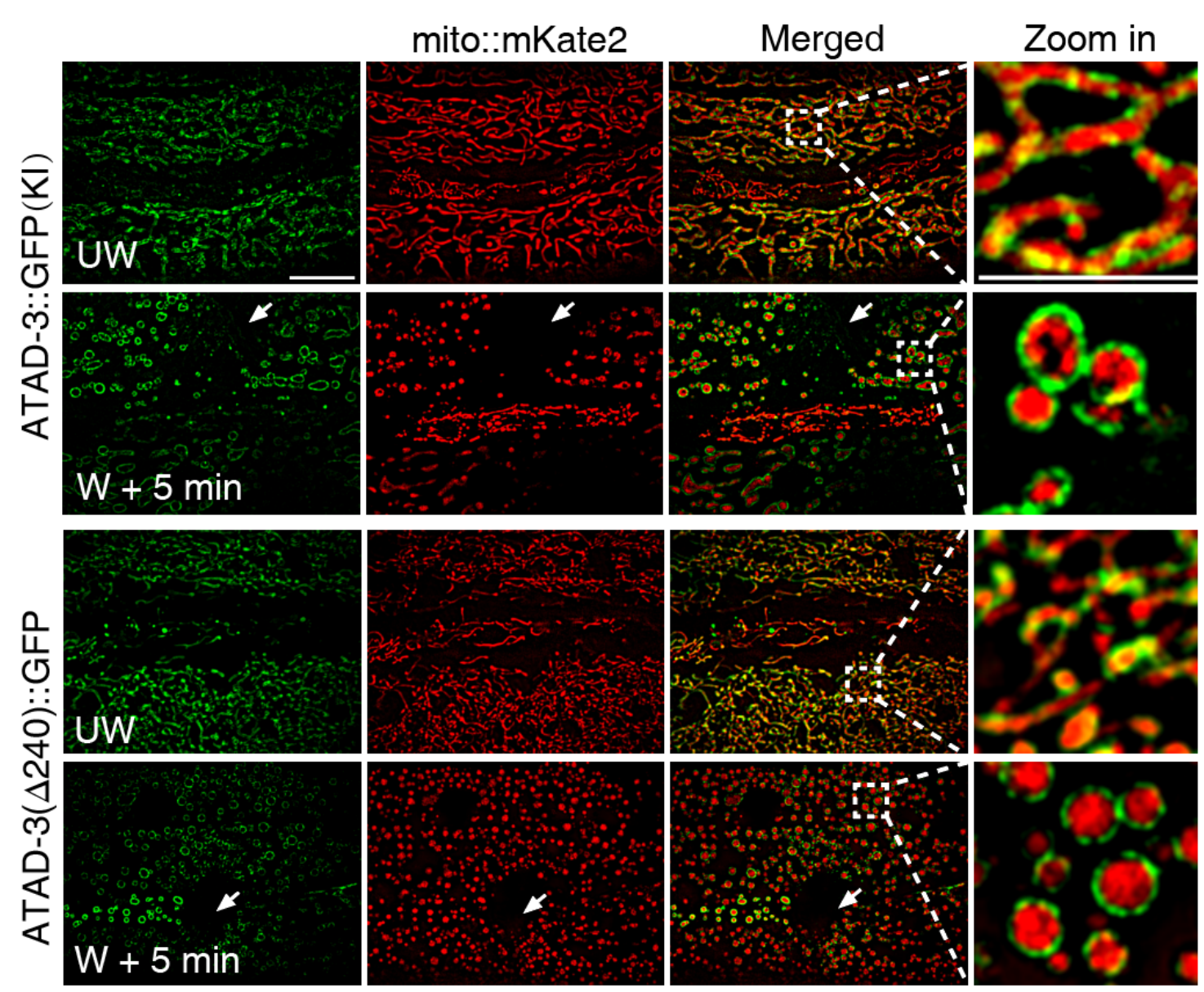

Response Fig.1

Response Fig. 1. Oligomerization-deficient mutants do not show clustering on mitochondria after injury. Representative HiS-SIM images of WT ATAD-3::GFP and ATAD-3(Δ240)::GFP co-labeled with mito::mKate2 before and 5 minutes after needle wounding. White arrows indicate the wound sites. Scale: 10 µm and 5 µm (zoom-in).

Reviewer #2 (Remarks to the Author):

I co-reviewed this manuscript with one of the reviewers who provided the listed reports. This is part of the Nature Communications initiative to facilitate training in peer review and to provide appropriate recognition for Early Career Researchers who co-review manuscripts.

Response:

We appreciate the transparency of *Nature Communications* in disclosing the co-review arrangement and welcome the journal's initiative to support the training and recognition of Early Career Researchers in the peer review process.

Reviewer #3 (Remarks to the Author):

Most of the previous concerns have been addressed. However, comment #2 still needs to be resolved. While the authors explained the rationale for the different wounding methods in their response, the context within the manuscript remains unclear. Regarding laser wounding, the authors now use the terms "small-scale" versus "large-scale." Please include definitions for these two scales (e.g., differences in time, intensity, etc.) in the Methods section under “Wounding Procedures.”

Response:

We thank the reviewer for this helpful comment. We apologize that the wound parameters remained insufficiently defined in the previous revision and have now added explicit definitions to the Methods section under "Wounding Procedures", as detailed below.

Small-scale laser wounding was performed at ~ 60% laser power, producing a wound diameter of ~1 μm. This approach generates highly localized plasma membrane disruption with minimal collateral tissue damage, and is used for experiments requiring precise spatial and temporal control of the injury stimulus, including live imaging of local mitochondrial Ca^{2+} dynamics and mitochondrial responses immediately following injury.

Large-scale laser wounding was performed at ~80% laser power, producing a wound diameter of ~10 µm. This approach generates a broader cellular injury more comparable in scale to needle wounding in C. elegans epidermis, and is used for experiments that require a more robust injury stimulus, including mitochondrial Ca^{2+} uptake wave propagation and other wound responses. We note that because laser power output varies across instruments, focal plane, and degrades over time with UV laser aging, wound diameter rather than laser power percentage should serve as the primary reproducibility parameter when adapting these protocols to other systems.

We have also carefully verified that the wounding method, scale, and relevant parameters are explicitly and consistently stated in the legend of every figure panel in which wounding is performed, and that all wounding terminology is consistent throughout the main text, figures, and Methods.

Additionally, please include the justification for choosing needle wounding for the RNAi screening in the Methods section. Furthermore, in line 580, Fig. 2b appears to be needle wounding, yet the figure legend refers to "large-scale laser wounding." Please clarify and correct it.

Response:
We thank the reviewer for both of these helpful observations. Regarding the choice of needle wounding for RNAi screening, we have added a clear justification to the Methods section under "Wounding Procedures."

“Needle wounding was selected for the large-scale RNAi screen because it is operationally straightforward, highly reproducible across large cohorts, and requires no specialized equipment except a dissection microscope, properties essential for high-throughput applications in which dozens of gene knockdowns must be evaluated in parallel. The larger wound area also generates a more robust injury response, increasing sensitivity for detecting partial loss-of-function phenotypes under incomplete RNAi knockdown conditions. Laser wounding was reserved for the mechanistic and quantitative imaging experiments in Figs. 2-5, where controlled wound geometry and precise temporal resolution of the injury stimulus are essential.”

We thank the reviewer for identifying the error in Fig. 2b annotation. Upon re-examination, we confirm that the left panel of Fig. 2b shows data acquired using needle wounding, while the right panel shows data acquired using large-scale laser wounding. The figure legend at line 580 has been revised to explicitly indicate the wounding method for each panel.

Also, in Fig. 6, please indicate the mitochondria and cytosol. Based on the figure legend, please also indicate MICU3.

Response: Corrected.

Finally, the TEM images in Supp. Fig. 2b are not high-resolution, and the cristae structures are barely visible. Please replace these with high-resolution images.

Response: Corrected.

REVIEWER COMMENTS (our point-by-point responses are included in blue text)

Reviewer #1 (Remarks to the Author):

Thank you for the responses. Please address these issues:

1) Line 3 and 4 of the abstract: " Here, we identify an alternative mitochondrial Ca2+ uptake pathway mediated by ….ATAD-3." As mentioned in previous reviews, this is a strong statement that is not proven in this paper, as it is yet uncertain if ATAD-3 is a bona fide conduit for Ca2+ entry. Please adjust here and throughout the paper accordingly.

Response: We thank the reviewer for this important point and agree that the original statement was stronger than supported by the current data. While our results show that ATAD-3 is required for rapid mitochondrial Ca^{2+} uptake following plasma membrane injury, they do not establish that ATAD-3 itself is a bona fide Ca^{2+} conduit.

To address this concern, we have revised the manuscript throughout to avoid implying that ATAD-3 functions as a direct channel. For example, we replaced "an alternative mitochondrial Ca^{2+} uptake pathway mediated by ATAD-3" with "an ATAD-3-dependent mechanism that promotes mitochondrial Ca^{2+} uptake following plasma membrane injury in *C. elegans*." We also revised or removed text that could be interpreted as presuming that ATAD-3 directly forms a Ca^{2+} conduit.

These changes have been incorporated into the Abstract, Results, and Discussion. In the revised Discussion, we now explicitly note that the mechanism by which ATAD-3 promotes mitochondrial Ca^{2+} uptake remains to be determined. Possible models include a direct role in Ca^{2+} transport, regulation of another mitochondrial Ca^{2+} transport system, or reorganization of the mitochondrial membrane environment to facilitate Ca^{2+} entry. Resolving these possibilities will require future biochemical, electrophysiological, and structural studies.

We sincerely appreciate the reviewer's comment, which helped us refine the wording of our conclusions and more accurately reflect the current level of evidence.

2) In the rebuttal letter, response to comment #3. The authors indicate that "In WT animals, wounding triggers a rapid and pronounced increase in ATAD-3::GFP clustering on mitochondria, manifesting as discrete, high-intensity foci on the mitochondrial surface that were distinct from the even mitochondrial distribution observed in unwounded animals. In contrast, ATAD-3(Δ240)::GFP failed to undergo injury-induced mitochondrial clustering, despite normal baseline co-localization with mito::mKate2 (Response Fig. 1). The intramitochondrial intensity distribution of these mutants remains uniform and indistinguishable from their pre-wound pattern following wounding. As these data were acquired by HiS-SIM images, which preclude robust quantitative analysis, we present representative images to illustrate the qualitative difference."

It is not clear to me how the authors made this interpretation from the images presebted. In WT animals, the ring-like ATAD3 clustering around the mitochondria is apparent and seems to be more intense than in unwounded conditions. In the mutant animal, it also appears that ATAD3 forms clusters in ring-like structures around the mitochondria following wounding, although seemingly, with lower intensity. As there was no quantification performed, it is difficult to assess if this intensity difference is real or not. The clustering distribution does not look appreciably different by eye as well.

Response: We thank the reviewer for this careful and constructive comment. We agree that the representative HiS-SIM images alone were not sufficient to support our previous statement that ATAD-3(Δ240)::GFP failed to undergo injury-induced mitochondrial clustering. As the reviewer pointed out, although ATAD-3::GFP animals exhibit a more pronounced ring-like pattern following wounding, the ATAD-3(Δ240)::GFP images also display some ring-like structures, making it difficult to conclude by visual inspection alone that clustering is abolished. We also agree that without quantitative analysis, any apparent difference in signal intensity cannot be rigorously interpreted.

To address this concern, we performed a new quantitative analysis of wound-induced ATAD-3 on SIM images. WT and ATAD-3(Δ240)::GFP animals co-expressing mito::mKate2 were imaged before and 5 min after needle injury. Using mito::mKate2 as a mitochondrial reference marker, we quantified the ATAD-3::GFP/mito::mKate2 fluorescence intensity ratio along

individual mitochondria before and after wounding. As shown in the new Supplementary Fig. 8f, wounding induced a significant increase in mitochondrial ATAD-3::GFP enrichment in WT animals, whereas no apparent increase was detected in ATAD-3(Δ240)::GFP animals after injury.

We also observed that ATAD-3(Δ240)::GFP exhibited a higher baseline signal than the WT protein under unwounded conditions. This difference likely reflects variation in transgene expression and/or basal mitochondrial association. Therefore, rather than comparing absolute fluorescence intensities between genotypes, we focused our analysis on the injury-induced change within each genotype, which provides a more appropriate measure of wound-dependent ATAD-3 redistribution.

Based on these new quantitative data, we have revised both the figure and the manuscript to avoid overstatement. We no longer state that ATAD-3(Δ240)::GFP completely lacks injury-induced clustering. Instead, we now conclude more cautiously that deletion of the coiled-coil domain impairs the wound-induced redistribution/enrichment of ATAD-3 on mitochondria. Thus, our conclusion is not that ring-like structures are absent in the mutant, but rather that the normal injury-induced increase in ATAD-3 mitochondrial accumulation observed in WT is attenuated in ATAD-3(Δ240)::GFP.

We sincerely appreciate the reviewer for raising this important point, which has helped us to strengthen the analysis and present these imaging data more rigorously and accurately.

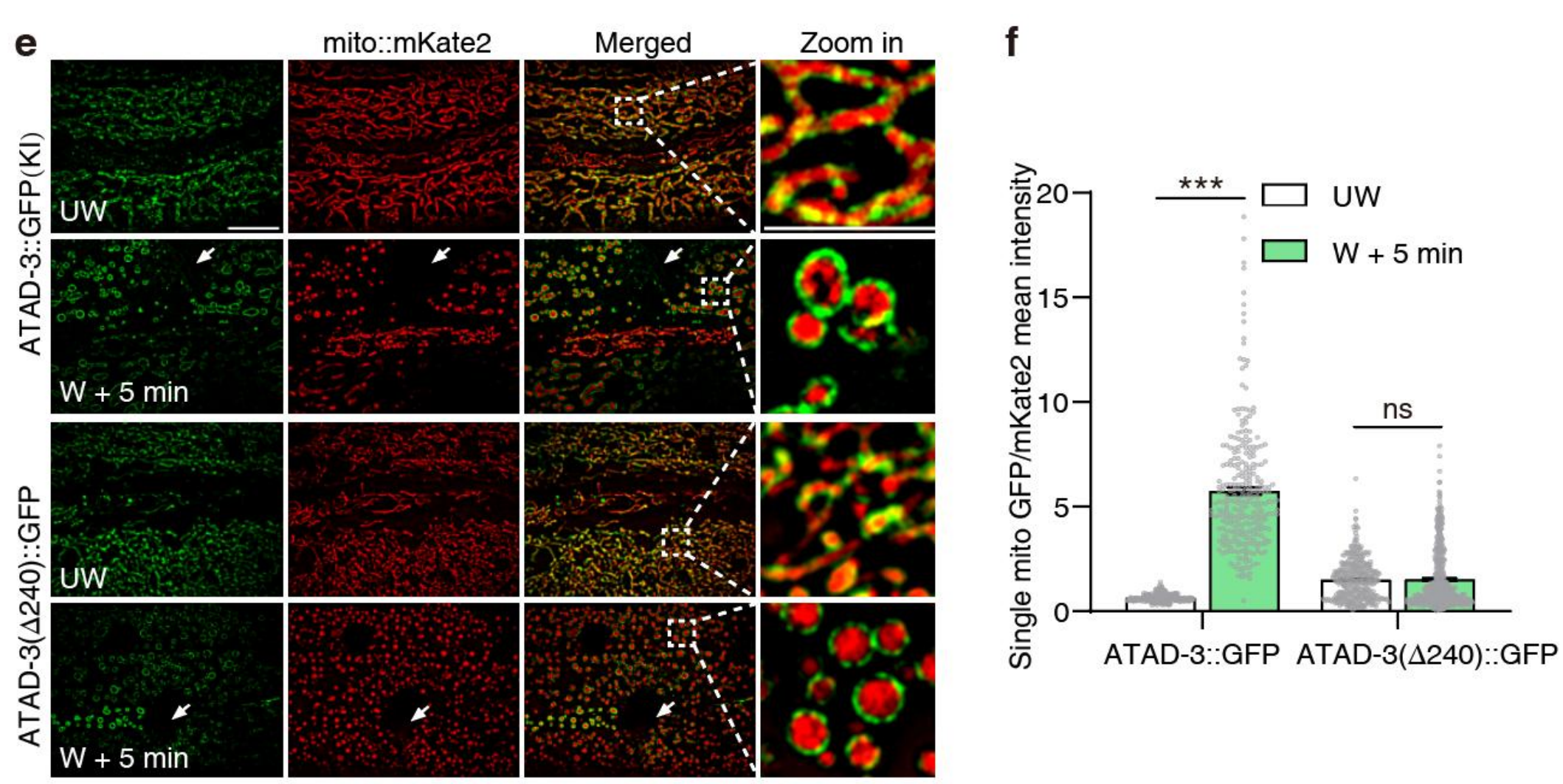

Figure S8e. Representative HiS-SIM images of WT ATAD-3::GFP and ATAD-3(Δ240)::GFP co-labeled with mito::mKate2 before and 5 minutes after needle wounding. White arrows indicate the wound sites. Scale: 10 μm and 5 μm (zoom-in).

Figure S8f. Quantification of the mean GFP/mKate2 fluorescence intensity ratio of individual mitochondria in ATAD-3::GFP and ATAD-3(Δ240)::GFP animals before and 5 minutes after needle wounding. Each dot represents one mitochondrion. $n \geq 277$ mitochondria from three independent animals per condition. Data are presented as mean ± SD. ***, $P < 0.001$, ns, $P > 0.05$, Student's t-test.

Reviewer #2 (Remarks to the Author):

I co-reviewed this manuscript with one of the reviewers who provided the listed reports. This is part of the Nature Communications initiative to facilitate training in peer review and to provide appropriate recognition for Early Career Researchers who co-review manuscripts.

Response: We thank the reviewers again for the constructive comments and suggestions.

Reviewer #3 (Remarks to the Author):

Revisions are adequate

Response: We sincerely thank the reviewers for the constructive comments and valuable suggestions, which have substantially improved our manuscript.
