# Supplementary material for: ATAD-3 couples plasma membrane injury to mitochondrial Ca2+ signaling
Source: Nat Commun. 2026 Sep 10;17:10690. doi: 10.1038/s41467-026-77690-y (PMC13655164; doi:10.1038/s41467-026-77690-y)
Supplement: Supplementary file 3 [file 41467_2026_77690_MOESM1_ESM.pdf]

ATAD-3 couples plasma membrane injury to mitochondrial Ca^{2+} signaling

Shiqi Xu, Yi Peng, Hengda Zhou, Xuecong Ren, Xin Wang, Zhenguang Ying, Meijiao Li, Yujie Sun, Bing Yang, Mao Zhang, Chonglin Yang, Weirui Ma, and Suhong Xu

Supplemental information includes:

Supplementary Figures 1 to 8 and Figure Legends

Supplementary Tables 1 to 4

Supplementary Figures and Figure Legends

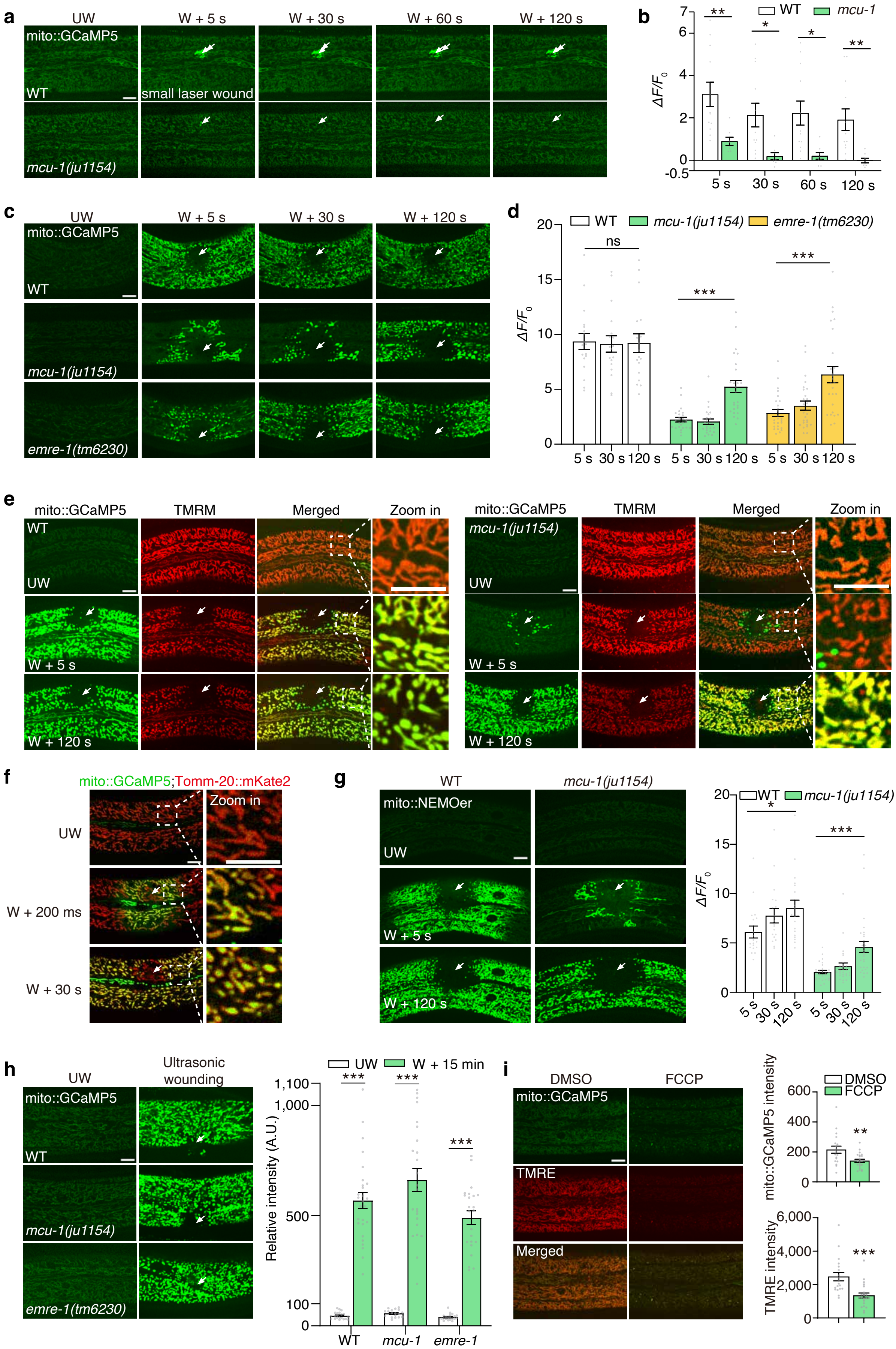

Supplementary Figure 1

Supplementary Figure 1. Wounding induces MCU-independent mitochondrial Ca^{2+} uptake

a. Representative confocal images of mito::GCaMP5 before and after small-scale laser wounding, creating a small wound in the multinucleated epidermal cell of wild-type (WT) and *mcu-1(ju1154)* mutant backgrounds. Imaging was performed using the P*col-19*-mito::GCaMP5*(juSi103)* single-copy insertion (SCI) transgenic strains. The white arrows indicate the wound sites. Scale: 10 μm.

b. Quantitative analysis of fluorescence intensity change ($\Delta F/F_0$) of mito::GCaMP5 at 5, 30, 60, and 120 seconds post- small-scale laser wounding. WT, n = 12; *mcu-1*, n = 8 worms. 5 s, P = 0.0117; 30 s, P = 0.0225; 60 s, P = 0.0188; 120 s, P = 0.0119. Two-way ANOVA with Šídák's multiple comparisons test.

c. Representative confocal images of mito::GCaMP5 before and after large-scale laser wounding, creating a large wound in the multinucleated epidermal cell of WT, *mcu-1(ju1154)*, and *emre-1(tm6230)* mutants. The white arrows indicate the wound sites. Scale: 10 μm.

d. Quantification of $\Delta F/F_0$ in mito::GCaMP5 intensity at 5, 30, and 120 seconds post-high-power laser wounding. WT, n = 18; *mcu-1*, n = 25; *emre-1*, n = 26 worms. 5 s vs 120 s: WT, P = 0.9121; *mcu-1*, P < 0.0001; *emre-1*, P < 0.0001. Two-way ANOVA with Dunnett's multiple comparisons test.

e. Confocal images of mito::GCaMP5 and TMRM staining before and after large-scale laser wounding in WT and *mcu-1(ju1154)* mutants. The white arrows indicate the wound sites. Scale: 10 μm (overview) and 5 μm (zoom in).

f. Confocal images of mito::GCaMP5 and Tomm20::mKate2 before and after large-scale laser wounding in WT animals. The white arrows indicate the wound sites. Scale: 10 μm (overview) and 5 μm (zoom in).

g. Left: Confocal images of mito::NEMOer in WT and *mcu-1(ju1154)* mutant before and after large-scale laser wounding. The white arrows indicate the wound sites. Scale: 10 μm. Right: Quantification of $\Delta F/F_0$ in mito::NEMOer intensity at 5, 30, and 120 seconds post-large-scale laser wounding. WT, n = 18; *mcu-1*, n = 28 worms. 5 s vs 120 s: WT, P = 0.0082; *mcu-1*, P = 0.0004. Two-way ANOVA with Dunnett's multiple comparisons test.

h. Left: Confocal images of mito::GCaMP5 before and 15 minutes after ultrasonic wounding in WT, *mcu-1(ju1154),* and *emre-1(tm6230)* mutants. The white arrows indicate the wound sites. Scale: 10 μm. Right: Quantification of mito::GCaMP5 intensity before and 15 minutes post-ultrasonic wounding. UW: n = 21, 18, 20 worms; W + 15 min: n = 27, 24, 23 worms. WT, P < 0.0001; *mcu-1*, P < 0.0001; *emre-1*, P < 0.0001. Mixed-effect analysis with Šídák's multiple comparisons test.

i. Left: Representative confocal images of mito::GCaMP5 fluorescence intensity and TMRE staining in DMSO (control) and FCCP treatment group. Scale: 10 μm. Right upper: Quantification of mito::GCaMP5 intensity in the control and FCCP treatment groups. Right lower: Quantification of TMRE intensity in the control and FCCP treatment group. DMSO, n = 19; FCCP, n = 22 worms. mito::GCaMP5, P = 0.0046; TMRE, P = 0.0003. Two-sided Student's t-test.

All data are presented as the mean ± SEM. ns, $P > 0.05$. * $P < 0.05$, **$P < 0.01$, ***$P < 0.001$.

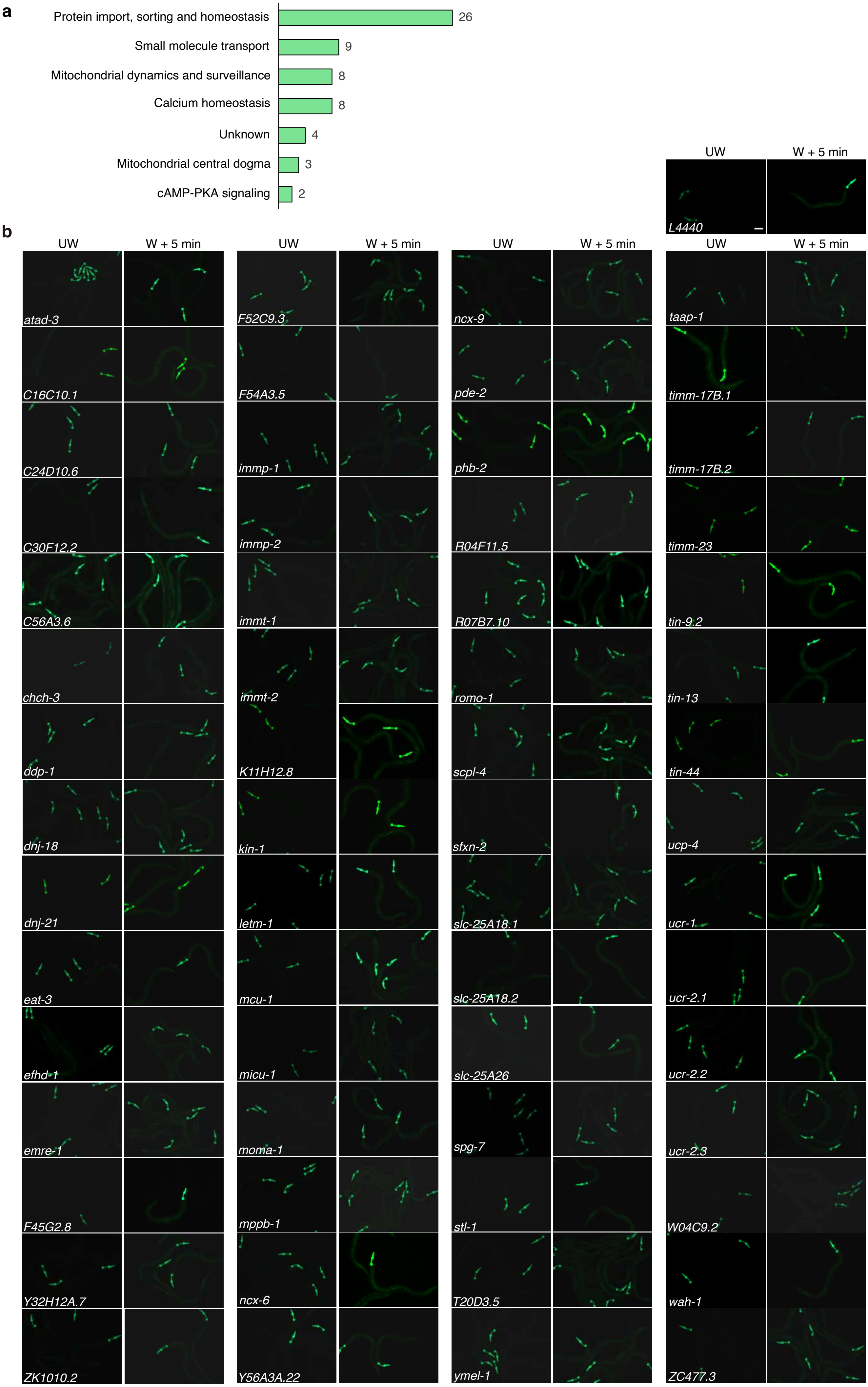

Supplementary Figure 2

Supplementary Figure 2. RNAi screen for regulators of mitochondrial Ca^{2+} uptake

a. Classification of candidate genes encoding inner mitochondrial membrane proteins targeted in RNAi screens.

b. Representative images of mito::GCaMP5 before and after needle wounding under various RNAi treatments captured using a dissecting microscope. Scale: 100 μm.

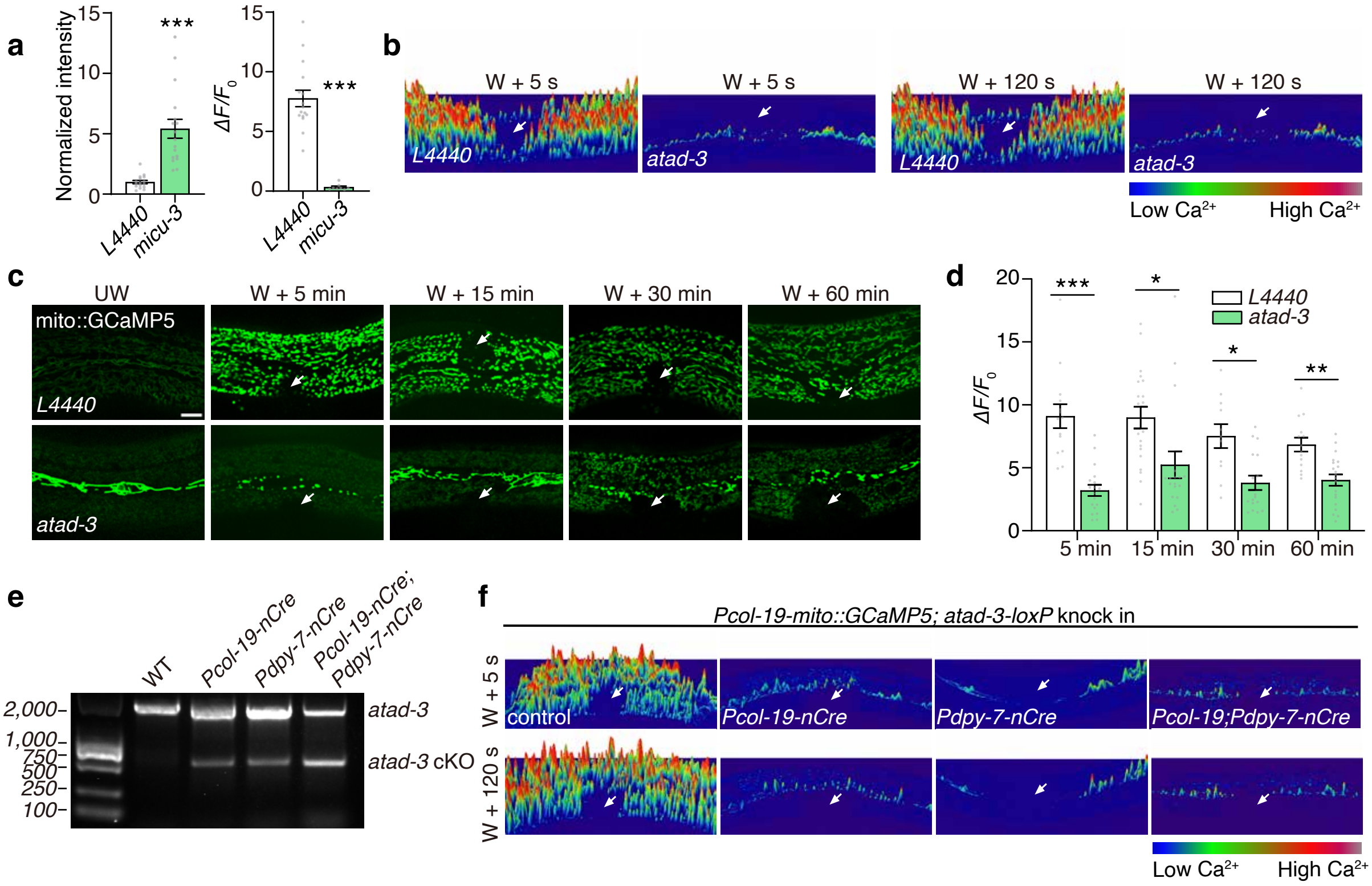

Supplementary Figure 3

Supplementary Figure 3. ATAD-3 is required for mitochondrial Ca^{2+} uptake after wounding

a. Left: Quantification analysis of basal mito::GCaMP5 fluorescent intensity in *L4440* (empty vector as control) and *micu-3* RNAi animals. n = 16, 17 worms. P <0.0001. Two-sided Student's t-test. Right: Quantification of $\Delta F/F_0$ in mito::GCaMP 5 minutes post-large-scale laser wounding. n = 16, 7 worms. P < 0.0001. Two-sided Student's t-test.

b. Surface plot analysis of mito::GCaMP5 intensity 5 and 120 seconds post-large scale laser wounding in *L4440* and *atad-3* RNAi animals. False-color rendering was applied using the Rainbow RGB LUT in ImageJ; the color scale bar indicates pixel intensity. The white arrows indicate the wound sites.

c. Representative confocal images of mito::GCaMP5 before and 5, 15, 30, and 60 minutes after needle wounding in *L4440* and *atad-3* RNAi animals. The white arrows indicate the wound sites. Scale, 10 μm.

d. Quantification of $\Delta F/F_0$ in mito::GCaMP5 at 5, 15, 30, and 60 minutes post-needle wounding in *L4440* and *atad-3* RNAi animals. *L4440* 5 min, n = 14; *L4440* 15 min, n = 20; *L4440* 30 min, n = 11; *L4440* 60 min, n = 14; *atad-3* 5 min, n = 18; *atad-3* 15 min, n = 18; *atad-3* 30 min, n = 17; *atad-3* 60 min, n = 20 worms. 5 min, P < 0.0001; 15 min, P = 0.0400; 30 min, P = 0.0145; 60 min, P = 0.0018. Mixed-effect analysis with Šídák's multiple comparisons test.

e. Agarose gel electrophoresis analysis of genomic DNA extracted from WT and *atad-3* conditional knockout strains. P*col-19-nCre (juSi262)*, P*dpy-7-nCre(zjuEx3269)*, and P*col-19*-nCre+P*dpy-7-nCre(zjuEx3284)* transgenic animals were used to knock out *atad-3* specifically in the epidermal cell.

f. Surface plot analysis of mito::GCaMP5 intensity before and after large-scale laser wounding in WT and *atad-3* conditional epidermal-specific knockout (cKO) mutants.

False-color rendering was applied using the Rainbow RGB LUT in ImageJ; the color scale bar indicates pixel intensity. The white arrows indicate the wound sites.

All data are presented as the mean ± SEM. * $P < 0.05$, ** $P < 0.01$, *** $P < 0.001$.

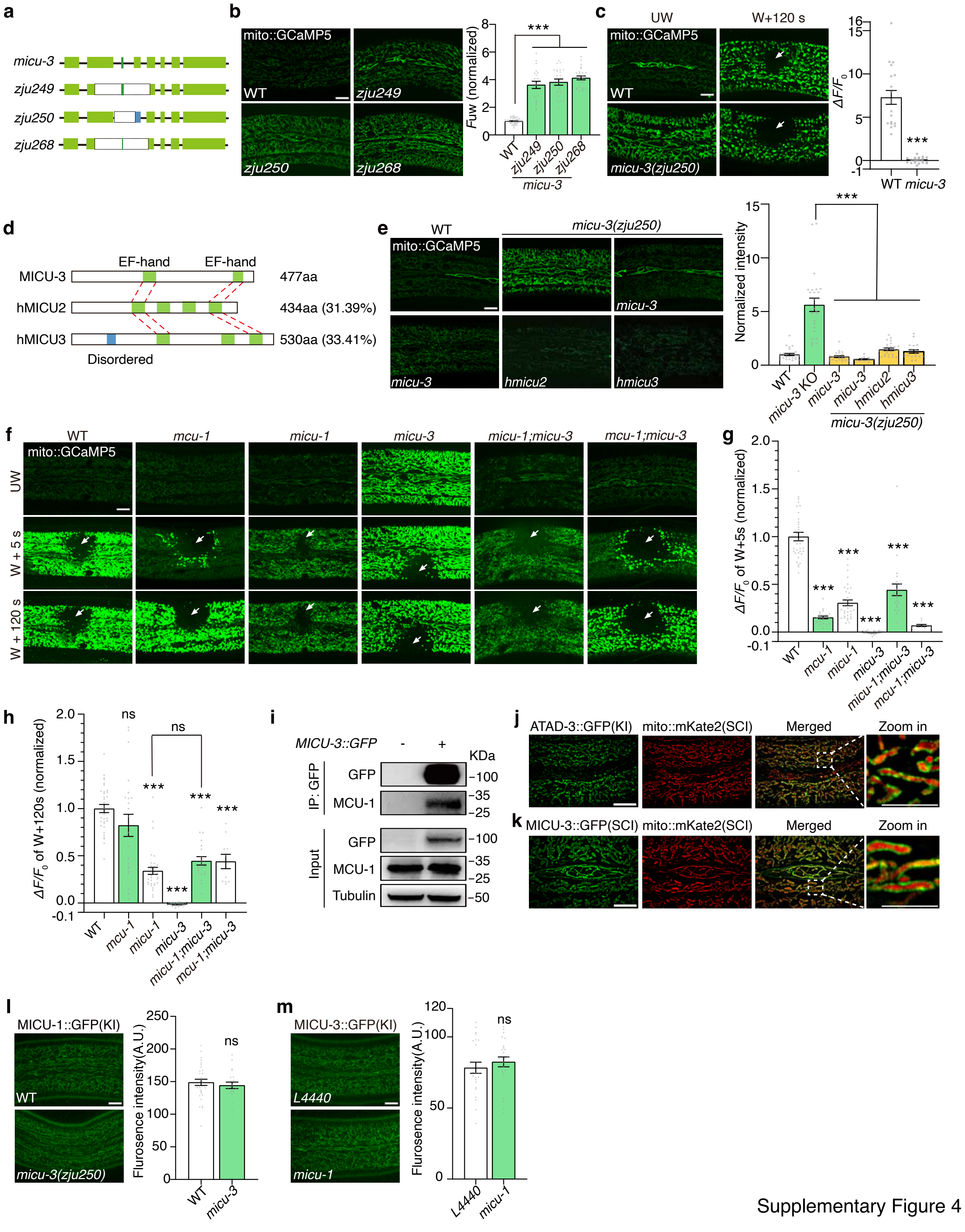

Supplementary Figure 4

Supplementary Figure 4. MICU-3 functions as a negative regulator of mitochondrial Ca^{2+} homeostasis

a. Schematic representation of the generated *micu-3* knockout mutants. Deleted regions are shown in white; inserted regions are highlighted in blue.

b. Left: Representative confocal images of mito::GCaMP5 fluorescence intensity in WT, *micu-3* deletion mutants (*zju249, zju250, and zju268*) under baseline conditions. Scale: 10 μm. Right: Quantification analysis of mito::GCaMP5 fluorescent intensity in WT, *micu-3* deletion mutants (*zju249, zju250, and zju268*) under baseline conditions, normalized to WT. n = 51, 21, 22, 26 worms. vs WT: *zju249,* $P < 0.0001$; *zju250,* $P < 0.0001$; *zju268,* $P < 0.0001$. One-way ANOVA with Dunnett's multiple comparisons test.

c. Left: Representative confocal images of mito::GCaMP5 intensity before and after large-scale laser wounding in WT and *micu-3 (zju250)* animals. The white arrows indicate the wound sites. Scale: 10 μm. Right: Quantification of $\Delta F/F_0$ in mito::GCaMP5 at 2 minutes post-wounding in WT and *micu-3(zju250)*. n = 19 worms. $P < 0.0001$. Two-sided Student's t-test.

d. Schematic representation of the protein structures of MICU-3 and hMICU2/3. The EF-hand domains are colored in green, with conserved EF-hand motifs highlighted by red dashed lines. The percentage identity of each human ortholog to *C. elegans* MICU-3 is indicated in parentheses.

e. Left: Representative confocal images of mito::GCaMP5 intensity in WT, *micu-3 (zju250)*, and *Pcol-19-micu-3(zjuEx2051)*, *Pcol-19-hmicu2(zjuEx2300)*, *Pcol-19-hmicu3(zjuEx2583)* transgenic animals. Scale: 10 μm. Right: Quantification analysis of mito::GCaMP5 fluorescent intensity in WT, *micu-3(zju250),* and rescue strains. n = 26, 24, 22, 14, 26, 19 worms. vs *micu-3* KO: $P < 0.0001$, < 0.0001, < 0.0001, < 0.0001. One-way ANOVA with Dunnett's multiple comparisons test.

f. Representative confocal images of mito::GCaMP5 signal in WT, *mcu-1(ju1154)*, *micu-1(ju1155)*, *micu-3(zju250)*, *micu-1(ju1155); micu-3(zju250)* double mutants*, and mcu-1(ju1154); micu-3(zju250)* double mutants before and 5, 120 seconds after large-scale laser wounding. The white arrows indicate the wound sites. Scale: 10 μm.

g. Quantification of basal mito::GCaMP5 intensity changes at 5 seconds post- large-scale laser wounding in WT, *mcu-1(ju1154)*, *micu-1(ju1155)*, *micu-3(zju250)*, *micu-1(ju1155); micu-3(zju250)* double mutants, and *mcu-1(ju1154); micu-3(zju250)* double mutants, normalized to WT. n = 33, 22, 34, 25, 22, 10 worms. vs WT: P < 0.0001, < 0.0001, < 0.0001, < 0.0001, < 0.0001. One-way ANOVA with Dunnett's multiple comparisons test.

h. Quantification of basal mito::GCaMP5 intensity changes at 120 seconds post-large-scale laser wounding in WT, *mcu-1(ju1154)*, *micu-1(ju1155)*, *micu-3(zju250)*, *mcu-1(ju1154); micu-3(zju250)* double mutants, and *micu-1(ju1155); micu-3(zju250)* double mutants, normalized to WT. n = 33, 22, 34, 25, 22, 10 worms. vs WT: P = 0.1026, P < 0.0001, P < 0.0001, P < 0.0001, P < 0.0001. *micu-1* vs *micu-1; micu-3*: P = 0.7484. One-way ANOVA with Tukey's multiple comparisons test.

i. Co-immunoprecipitation of MCU-1 with MICU-3. MICU-3::GFP (*zjuSi345*) SCI animals were used in this experiment. Lysates were incubated with GFP nanobody-conjugated beads, and precipitates and lysates were immunoblotted with antibodies against GFP, MCU-1, and Tubulin.

j. Representative HiS-SIM images of ATAD-3::GFP and mito::mKate2 colocalization in the epidermal cell. Scale: 10 μm and 5 μm (zoom-in).

k. Representative HiS-SIM images of MICU-3::GFP and mito::mKate2 colocalization in WT animals. Scale: 10 μm and 5 μm (zoom-in).

l. Left: Representative confocal images of MICU-1::GFP in WT and *micu-3(zju250)* animals. Scale: 10 μm. Right: Quantification of GFP intensity in WT and *micu-3(zju250)* animals. n = 32, 21 worms. P = 0.5214. Two-sided Student's t-test.

m. Left: Representative confocal images of MICU-3::GFP in control and *micu-1* RNAi animals. Scale: 10 μm. Right: Quantification of GFP intensity in control and *micu-1* RNAi animals. *n* = 24, 24 worms. *P* = 0.4350. Two-sided Student's t-test.

All data are presented as the mean ± SEM. ns, *P* > 0.05. ****P* < 0.001.

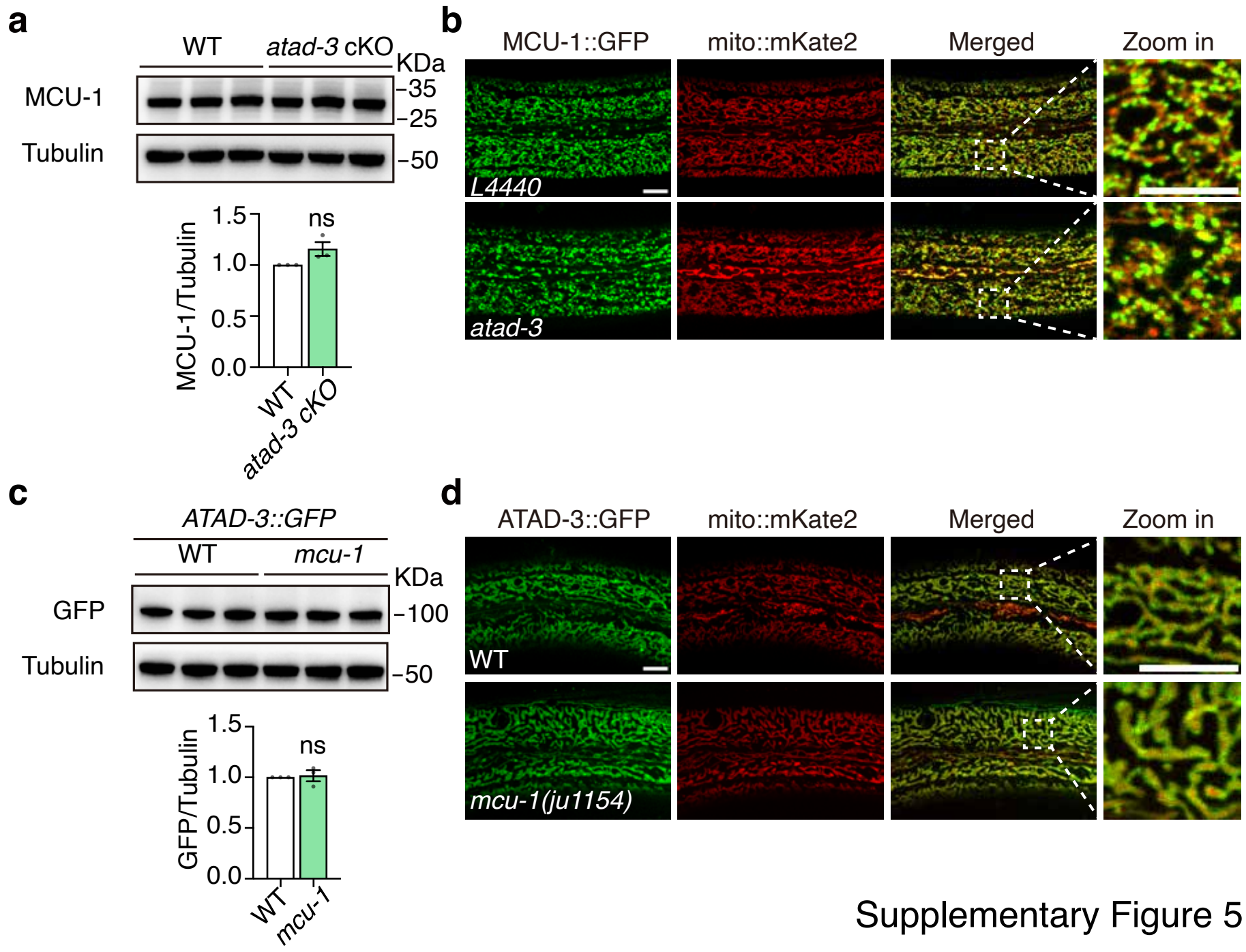

Supplementary Figure 5

Supplementary Figure 5. ATAD-3 does not interact with MCU-1 *in vivo*

a. Top: Western blot analysis of MCU-1 protein expression in WT and *atad-3* epidermal-specific knockout strains. MCU-1::GFP*(juSi236)* SCI was used in this experiment. Bottom: quantification of the western blot of MCU-1 protein expression. $n = 3$ independent experiments. $P = 0.0835$. Two-sided Student's t-test.

b. Representative confocal images of MCU-1::GFP and mito::mKate2 colocalization in control (*L4440*) and *atad-3* RNAi strains. Scale: 10 µm and 5 µm (zoom-in).

c. Top: western blot analysis of ATAD-3::GFP protein expression in WT and *mcu-1(ju1154)* deletion mutants. *ATAD::GFP (zju283)* knock-in was used in this experiment. Bottom: quantification of the western blot of ATAD-3::GFP protein expression. $n = 3$ independent experiments. $P = 0.7914$. Two-sided Student's t-test.

d. Representative confocal images of ATAD-3::GFP and mito::mKate2 colocalization in WT and *mcu-1(ju1154)* deletion mutants. Scale: 10 µm and 5 µm (zoom-in).

All data are presented as the mean ± SEM. ns, $P > 0.05$.

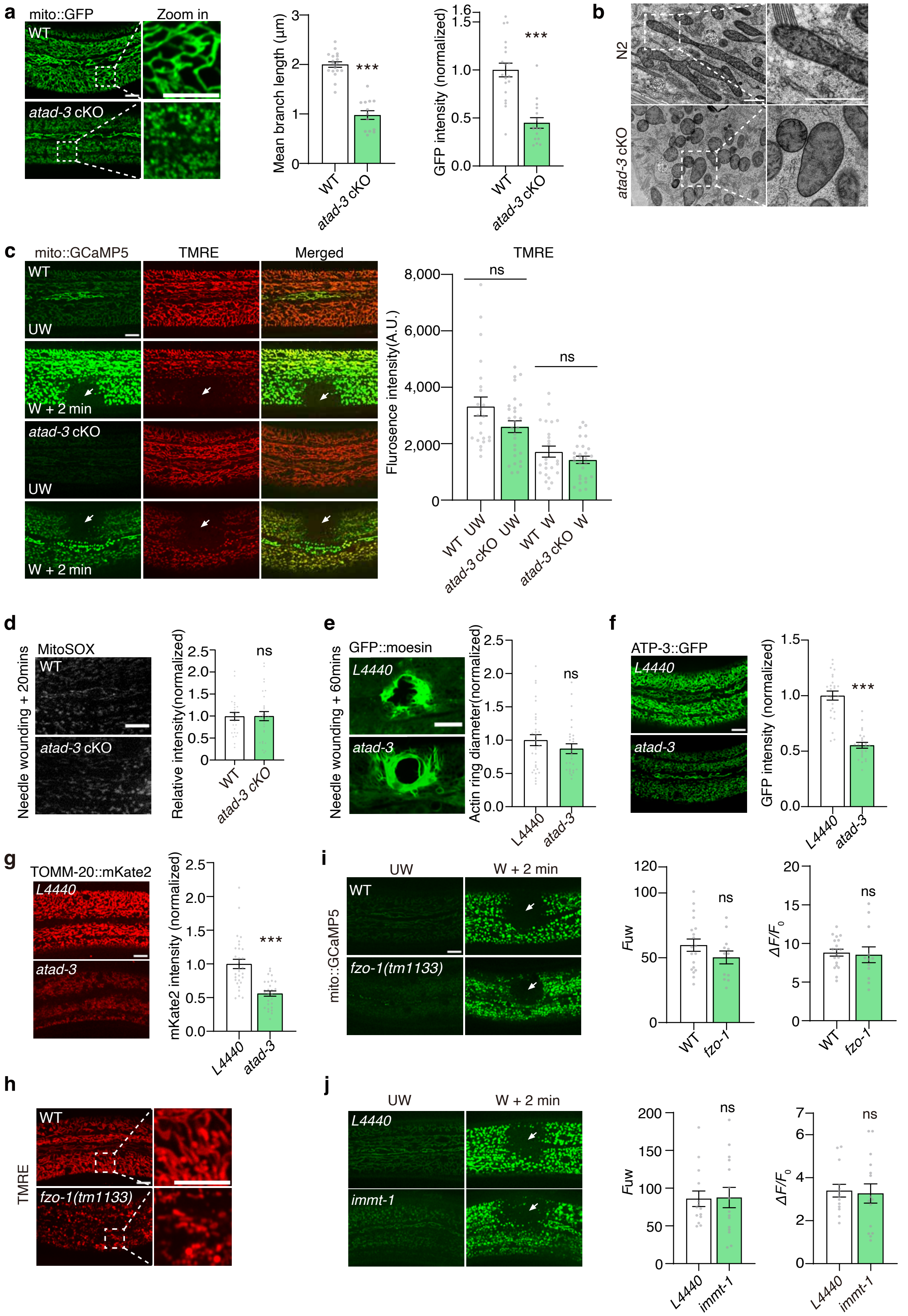

Supplementary Figure 6

Supplementary Figure 6. Mitochondrial Ca^{2+} uptake is independent of mitochondrial architecture and membrane potential

a. Left: Representative confocal images of mito::GFP in WT and *atad-3* epidermal-specific knockout strains. Scale: 10 μm and 5 μm (zoom-in). Middle: Quantification of mean branch length analyzed by the *ImageJ* plugin Mitochondrial Analyzer. *n* = 19, 13 worms. *P* < 0.0001. Two-sided Student's t-test. Right: Quantification of GFP intensity in WT and *atad-3* epidermal-specific knockout strains. *n* = 20, 16 worms. *P* < 0.0001. Two-sided Student's t-test.

b. TEM images of mitochondria in the epidermis of WT and *atad-3* epidermal-specific knockout strains. Scale: 1 μm.

c. Left: Representative confocal images of mito::GCaMP5 (green) signal and TMRE (red) staining in WT and *atad-3* cKO strains before and after large-scale laser wounding. The white arrows indicate the wound sites. Scale: 10 μm. Right: Quantification of TMRE intensity in WT and *atad-3* cKO strains before and after large-scale wounding. *n* = 23, 28, 24, 28 worms. WT vs *atad-3* cKO: UW, *P* = 0.1085; W, *P* = 0.7821. One-way ANOVA with Tukey's multiple comparisons test.

d. Left: Confocal images of mitoSOX staining in wild-type and epidermal-specific *atad-3 knockout* animals after needle wounding. Scale: 10 μm. Right: Quantification of mitoSOX intensity, normalized to control. *n* = 23, 29 worms. *P* = 0.9671. Two-sided Student's t-test.

e. Left: Confocal images of GFP::moesin assembly after needle wounding in control and *atad-3 RNAi* animals. Scale: 10 μm. Right: Quantification of actin ring diameter, r, normalized to control. *n* = 32, 27 worms. *P* = 0.2571. Two-sided Student's t-test.

f. Left: Representative confocal images of ATP-3::GFP in control and *atad-3* RNAi animals. Scale: 10 μm. Right: Quantification of GFP fluorescent intensity in control and *atad-3* RNAi animals, normalized to control. *n* = 25, 23 worms. *P* < 0.0001. Two-sided Student's t-test.

g. Left: Representative confocal images of TOMM-20::mKate2 in control and *atad-3* RNAi animals. Scale: 10 µm. Right: Quantification of mKate2 fluorescent intensity in control and *atad-3* RNAi animals, normalized to control. *n* = 30, 27 worms. $P <$ 0.0001. Two-sided Student's t-test.

h. Representative confocal images of TMRE staining in WT and *fzo-1 (tm1133)* animals. Scale: 10 µm and 5 µm (zoom-in).

i. Left: Representative confocal images of mito::GCaMP5 fluorescence intensity in WT and *fzo-1 (tm1133)* animals before and after large-scale laser wounding. The white arrows indicate the wound sites. Scale: 10 µm. Middle: Quantification of basal mito::GCaMP5 intensity in WT and *fzo-1 (tm1133)* animals. *n* = 19, 12 worms. $P =$ 0.1928. Two-sided Student's t-test. Right: Quantification of $\Delta F/F_0$ in mito::GCaMP5 intensity 120 s post-large-scale laser wounding in WT and *fzo-1 (tm1133)* animals. *n* = 19, 12 worms. $P = 0.7732$. Two-sided Student's t-test.

j. Left: Representative confocal images of mito::GCaMP5 fluorescence intensity in control and *immt-1* RNAi animals. The white arrows indicate the wound sites. Scale: 10 µm. Middle: Quantification of basal mito::GCaMP5 intensity in WT and *immt-1* RNAi animals. *n* = 14, 15 worms. $P = 0.9294$. Two-sided Student's t-test. Right: Quantification of $\Delta F/F_0$ in mito::GCaMP5 intensity 120s post-large-scale laser wounding in control and *immt-1* RNAi animals. *n* = 14, 15 worms. $P = 0.8158$. Two-sided Student's t-test.

All data are presented as the mean ± SEM. ns, $P > 0.05$. ***$P < 0.001$.

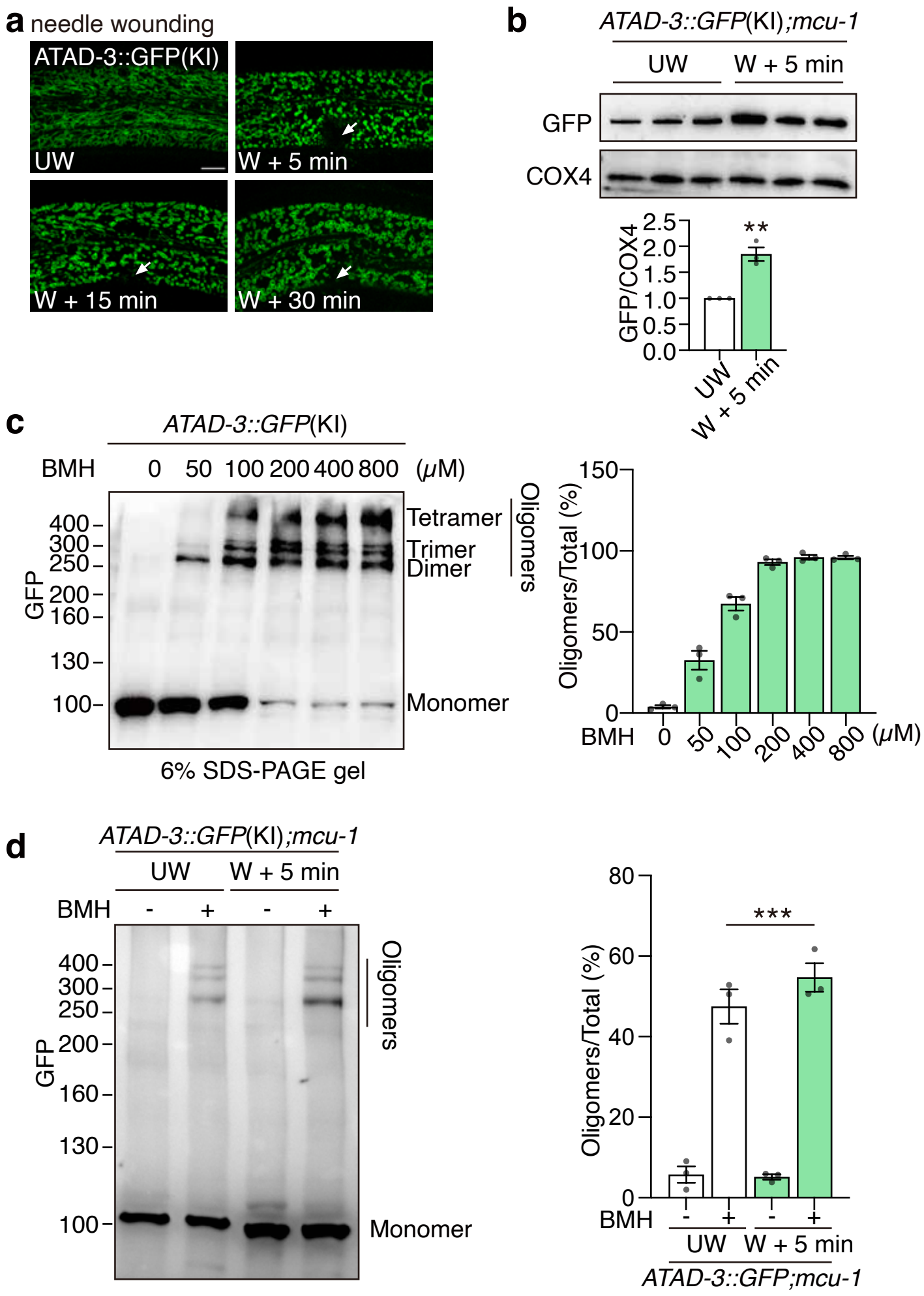

Supplementary Figure 7

Supplementary Figure 7. Wounding promotes ATAD-3 oligomerization and mitochondrial accumulation *in vivo*

a. Representative confocal images of ATAD-3::GFP before and 5, 15, and 30 minutes after needle wounding. The white arrows indicate the wound sites. Scale: 10 μm.

b. Left: Western blot analysis of ATAD-3::GFP expression in *mcu-1*(ju1154) mutants before and 5 minutes after ultrasonic wounding. *ATAD::GFP (zju283)* knock-in was used in this experiment. Right: Quantification of ATAD-3::GFP blots before and 5 minutes after ultrasonic wounding. *n* = 3 independent experiments. *P* = 0.0030. Two-sided Student's t-test.

c. Left: SDS-PAGE analysis (6% gel) of ATAD-3::GFP following treatment with increasing concentrations of BMH, indicating oligomer formation at 100 μM BMH. Right: Quantification of ATAD-3::GFP oligomer ratio following treatment with different BMH concentrations. *n* = 3 independent experiments.

d. Left: SDS-PAGE analysis (6% gel) of ATAD-3::GFP in the *mcu-1* mutant before and 5 minutes after needle wounding, showing enhanced oligomerization of ATAD-3::GFP in the *mcu-1(ju1154)* mutants. *ATAD::GFP (zju283)* knock-in was used in this experiment. Right: Quantification of ATAD-3::GFP oligomer ratio in the *mcu-1* mutants before and 5 minutes after needle wounding with BMH treatment. *n* = 3 independent experiments. UW with BMH vs W + 5 min with BMH: *P* < 0.0001, One-way ANOVA with Tukey's multiple comparisons test.

All data are presented as the mean ± SEM. ***P* < 0.01, ****P* < 0.001.

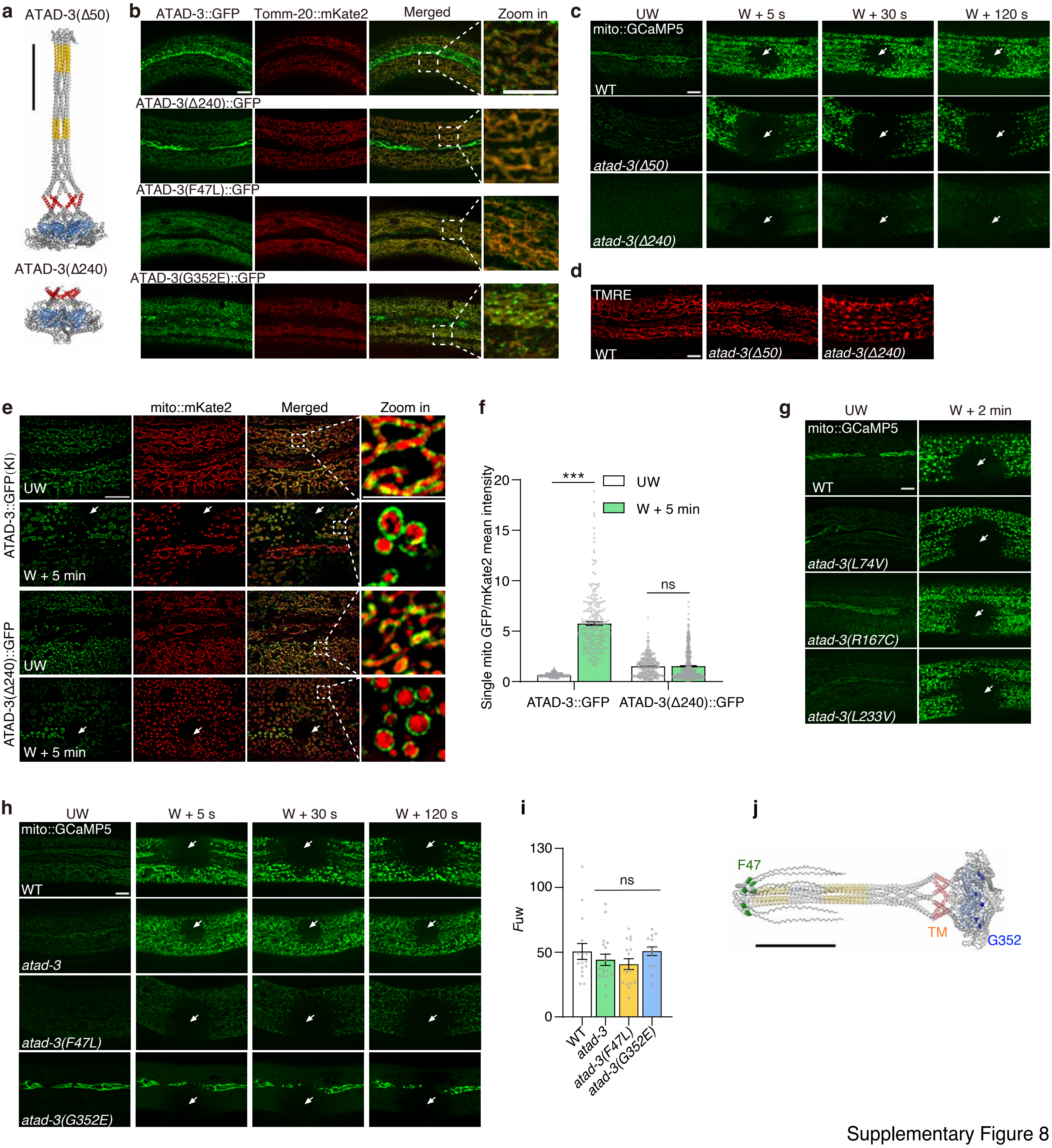

Supplementary Figure 8

Supplementary Figure 8. Disease-associated ATAD-3 variants impair mitochondrial Ca^{2+} uptake

a. Predicted protein structures of ATAD-3(Δ50) and ATAD-3(Δ240) truncation mutants.

b. Representative confocal images of transgenic worms expressing GFP-tagged ATAD-3 variants and Tomm-20::mKate2 colocalization. Scale: 10 µm and 5 µm (zoom-in).

c. Representative confocal images of mito::GCaMP5 fluorescence intensity in WT, *ATAD-3(Δ50)*, and *ATAD-3(Δ240)* overexpression strains before, 5, 30, and 120 seconds after large-scale laser wounding. The white arrows indicate the wound sites. Scale: 10 µm.

d. Representative confocal images of TMRE staining in WT, *ATAD-3(Δ50)*, and *ATAD-3(Δ240)* overexpression strains. Scale: 10 µm.

e. Representative HiS-SIM images of WT ATAD-3::GFP and ATAD-3(Δ240)::GFP co-labeled with mito::mKate2 before and 5 minutes after needle wounding. White arrows indicate the wound sites. Scale: 10 µm and 5 µm (zoom-in).

f. Quantification of the mean GFP/mKate2 fluorescence intensity ratio of individual mitochondria in ATAD-3::GFP and ATAD-3(Δ240)::GFP animals before and 5 minutes after needle wounding. Individual mitochondria were identified using the mito::mKate2 signal as described in the Methods. Each dot represents one mitochondrion. *n* =308, 277, 385, 491 mitochondria from three independent animals per condition. UW vs W + 5 min: ATAD-3::GFP, $P < 0.0001$; ATAD-3(Δ240)::GFP, $P = 0.9994$. Two-sided Student's t-test.

g. Representative confocal images of mito::GCaMP5 fluorescence intensity in WT, disease variants *ATAD-3(L74V)*, *ATAD-3(R167C)*, and *ATAD-3(L233V)*

overexpression strains before and after large-scale laser wounding. The white arrows indicate the wound sites. Scale: 10 µm.

h. Representative confocal images of mito::GCaMP5 fluorescence intensity in WT, *ATAD-3 cDNA*, disease variants *ATAD-3(F47L)*, and *ATAD-3(G352E)* overexpression strains before, 5, 30, and 120 seconds after large-scale laser wounding. The white arrows indicate the wound sites. Scale: 10 µm.

i. Quantification of basal mito::GCaMP5 intensity in WT, *ATAD-3 cDNA*, disease variants *ATAD-3(F47L)*, and *ATAD-3(G352E)* overexpression strains. n = 16, 18, 16, 14 worms. vs WT: P = 0.6371, 0.3285, >0.9999. One-way ANOVA with Dunnett's multiple comparisons test.

j. Predicted 3D structure of ATAD-3, highlighting the positions of key residues analyzed. Scale: 10 nm.

All data are presented as the mean ± SEM. ns, $P > 0.05$. ***$P < 0.001$.

Supplementary Table 1. A list of strains used in this study

**Strains and alleles**	**Source**	**Code**
C. elegans N2 Bristol	CGC	N2
P*col-19*-mito::GCaMP5 I	*Xu and Chisholm, 2014*	CZ19107
P*col-19*-mito(cox8)::GCaMP5*(juSi103)* I*; mcu-1(ju1154)* IV	CGC	CZ19983
P*col-19*-mito(cox8)::GCaMP5*(juSi103)* I*; emre-1(tm6230)* X	This study	SHX5328
P*col-19*-mito(cox8)::GCaMP5*(juSi103)* I; P*hyp-7*-tomm-20-linker-mKate2-tbb-2UTR*(zjuSi126)*	This study	SHX6135
P*col-19*-mt-NEMOs*(zjuEx3028)*	This study	SHX5116
P*col-19*-mt-NEMOs*(zjuEx3028)*; *mcu-1(ju1154)* IV	This study	SHX6196
FRT-hygR-FRT-P*col-19*-RDE-1-P*myo-2::*GFP*(zjuSi54)* IV *; rde-1(ne219)* V; P*col-19*-mito(cox8)::GCaMP5*(juSi103)* I	This study	SHX2690
atad-3-loxpA*(SYB6373);atad-3*-loxpB*(SYB6569)*	This study	SHX4153
P*col-19*-nCre*(juSi262)* V		CZ22396
P*col-19*-mito(cox8)::GCaMP5*(jusi103);atad-3*-loxpA*(SYB6373);atad-3*-loxpB*(SYB6569);*P*col-19*-nCre*(juSi262)*	This study	SHX4176
P*col-19*-mito(cox8)::GCaMP5*(juSi103);*P*dpy-7*-nCre*(zjuEx3261); atad-3*-loxpA*(SYB6373);atad-3*-loxpB*(SYB6569)*	This study	SHX5379
P*col-19*-mito(cox8)::GCaMP5*(juSi103);* P*col-19*-nCre*(juSi262);* P*dpy-7*-nCre*(zjuEx3261); atad-3*-loxpA*(SYB6373);atad-3*-loxpB*(SYB6569)*	This study	SHX5447
P*col-19*-mito(cox8)::GCaMP5*(jusi103);atad-3*-loxpA*(SYB6373);atad-3*-loxpB*(SYB6569);*P*col-*	This study	SHX5471

19-nCre*(juSi262)* ; P*col-182-atad-3* cDNA*(zjuEx3140)*		
P*col-19*-mito(cox8)::GCaMP5*(juSi103);* P*col-19*-nCre*(juSi262);* P*hyp-7*-nCre*(zjuEx3273); atad-3*-loxpA*(SYB6373);atad-3*-loxpB*(SYB6569)*	This study	SHX5401
micu-3 (C56A3.6) KO(zju249) V	This study	SHX3188
micu-3(C56A3.6) KO(zju250) V	This study	SHX3190
micu-3(C56A3.6) KO(zju268) V	This study	SHX3505
P*col-19*-mito(cox8)::GCaMP5*(juSi103)* I*; micu-3 (C56A3.6) KO(zju249)* V	This study	SHX3310
P*col-19*-mito(cox8)::GCaMP5*(juSi103)* I*; micu-3(C56A3.6) KO(zju250)* V	This study	SHX3311
P*col-19*-mito(cox8)::GCaMP5*(juSi103)* I*; micu-3(C56A3.6) KO(zju268)* V	This study	SHX4093
P*col-19*-mito(cox8)::GCaMP5*(juSi103);micu-3 KO(zju250);rde-1(ne219);*FRT-hygR-FRT-P*col-19*-RDE-1-P*myo-2*-GFP*(zjuSi54)*	This study	SHX4541
*mcu-1(ju1154);*P*col-19*-mito(cox8)::GCaMP5*(jusi103);atad-3*-loxpA*(SYB6373);atad-3*-loxpB*(SYB6569);*P*col-19*-nCre*(juSi262)*	This study	SHX5161
ATAD-3::GFP *(zju283)*	This study	SHX4467
loxP-P*col-19*-VDAC-1::GFP-myc*(zjuSi268)*	*Ren et al., 2023*	SHX2880
P*col-19*-MCU-1::GFP*(juSi236)* V	CGC	CZ21782
P*col-19*-MCU-1::GFP*(juSi236)* V ; *atad-3*-loxpA*(SYB6373);atad-3*-loxpB*(SYB6569);*P*col-19*-nCre*(juSi262)*	This study	SHX6172
P*col-19*-mito-mKate2*(zjuSi404);*P*col-19*-MCU-1::GFP*(juSi236);rde-1(ne219);*FRT-hygR-FRT-P*col-19*-RDE-1-P*myo-2*-GFP*(zjuSi54)*	This study	SHX4572
ATAD-3::GFP*(zju283);mcu-1(ju1154)* IV	This study	SHX4606

P*col-19*-mito(cox8)::GCaMP5*(juSi103);*P*col-19*-hMICU2::GFP-myc*(zjuEx2300); micu-3 KO(zju250)* V	This study	SHX4125
P*col-19*-mito(cox8)::GCaMP5*(juSi103);*P*col-19*-hMICU3::GFP-myc*(zjuEx2583);micu-3 KO(zju250)* V	This study	SHX4547
P*col-19*-mito(cox8)::GCaMP5*(juSi103); micu-3 KO(zju250)* V;P*col-182-micu-3* genome*(zjuEx2051)*	This study	SHX3780
P*col-19*-mito(cox8)::GCaMP5*(juSi103);* P*col-182-micu-3* genome*(zjuEx1890)*	This study	SHX3500
Loxp-P*col-19*-mito::mKate2*(zjuSi47);* LoxP-P*col-19*- MICU-3::GFP-myc*(zjuSi345)*	This study	SHX3810
ATAD-3::GFP*(zju283);*P*col-19*-mito::mKate2*(zjuSi404)*	This study	SHX4539
MICU-1::GFP*(ju1783)* IV	*Tang et al., 2020*	CZ27508
MICU-1::GFP*(ju1783)* IV; *micu-3 KO(zju250)* V	This study	SHX6330
ATAD-3::GFP*(zju283);*P*col-19*-mito::mKate2*(zjuSi404)*; *mcu-1(ju1154)* IV	This study	SHX5375
P*col-19*-mito(cox8)::GCaMP5*(juSi103);mcu-1(ju1154)* IV*;micu-3 KO(zju250)* V	This study	SHX3497
P*col-19*-mito-GFP*(zjuSi197)* I	This study	SHX2157
P*hyp-7*-tomm-20-linker-mKate2-tbb-2UTR*(zjuSi126)*	This study	SHX1821
ATP::GFP(zju133)	This study	SHX280
P*col-19*-mito(cox8)::GCaMP5*(juSi103);fzo-1(tm1133)*	This study	CZ19108
P*col-182*-mito(cox8)::GCaMP5*(juSi103)* I*;* P*col-182*-atad-3(50-595)*(zjuEx3136)*	This study	SHX5323
P*col-182*-mito(cox8)::GCaMP5*(juSi103)* I*;* P*col-1*-atad-3(240-595)*(zjuEx3144)*	This study	SHX5322

P*col-19*-ATAD-3-GFP*(zjuEx3147);*P*hyp-7*-tomm-20-linker-mKate2-tbb-2UTR*(zjuSi126)*	This study	SHX5306
P*col-19*-ATAD-3(240-595)-GFP*(zjuEx3129);* P*hyp-7*-tomm-20-linker-mKate2-tbb-2UTR*(zjuSi126)*	This study	SHX5254
P*col-19*-ATAD-3(F47L)-GFP*(zjuEx 3512);* P*hyp-7*-tomm-20-linker-mKate2-tbb-2UTR*(zjuSi126)*	This study	SHX5899
P*col-19*-ATAD-3(G352D)-GFP*(zjuEx3509);* P*hyp-7*-tomm-20-linker-mKate2-tbb-2UTR*(zjuSi126)*	This study	SHX5805
P*col-19*-ATAD-3(G352E)-GFP*(zjuEx3508);* P*hyp-7*-tomm-20-linker-mKate2-tbb-2UTR*(zjuSi126)*	This study	SHX5804
P*col-19*-mito(cox8)::GCaMP5*(juSi103)* I*;* P*col-182*-ATAD-3(F47L)*(zjuEx3126)*	This study	SHX5251
P*col-19*-mito(cox8)::GCaMP5*(juSi103)* I*;* P*col-182*-ATAD-3 cDNA*(zjuEx2008)*	This study	SHX3722
P*col-19*-mito(cox8)::GCaMP5*(juSi103)* I*;* P*col-182*-ATAD-3(G352E)*(zjuEx3156)*	This study	SHX5325
P*col-19*-mito(cox8)::GCaMP5*(juSi103)* I*;* P*col-182*-ATAD-3(L74V)*(zjuEx3120)*	This study	SHX5245
P*col-19*-mito(cox8)::GCaMP5*(juSi103)* I*;* P*col-182*-ATAD-3(R167C)*(zjuEx3127)*	This study	SHX5252
P*col-19*-mito(cox8)::GCaMP5*(juSi103)* I*;* P*col-182*-ATAD-3(L233V)*(zjuEx3124)*	This study	SHX5249

Supplementary Table 2. A list of plasmids used in this study

Plasmids	Source	Code
pU6-sgRNA-*atad-3* C-Ter-1	This study	pSX3187
pU6-sgRNA-*atad-3* C-Ter-2	This study	pSX3188
ATAD-3::GFP knock in repair template	This study	pSX3028
P*eft-3*-cas9-NLS-Pu6-*dpy-10* sgRNA	*Arribere et al., 2014*	pSX524
pDEST4641-SEC-P*col-19*-mito::mKate2	This study	pSX3275
pDEST4641-SEC-R1-R2 CRISPR modified for Chr III Single copy insertion template DEST	This study	pSX2601
P*ttx-3*-RFP	*Xu and Chisholm, 2016*	pSX35
P*myo-2*-mcherry	*Meng et al., 2020*	pSX375
pCFJ210-SEC-RIR2 CRISPR modified for Chr I Single copy insertion template DEST	*Meng et al., 2020*	pSX621
pU6-sgRNA-*micu-3* #1	This study	pSX2572
pU6-sgRNA-*micu-3* #2	This study	pSX2573
P*col-19*-hMICU-2 cDNA-GFP-myc	This study	pSX3234
P*col-19*-hMICU-3 cDNA-GFP-myc	This study	pSX3235
P*col-182-micu-3* genome	This study	pSX2768
pCFJ210-LoxP-SEC-LoxP-P*col-19*-MICU-3::GFP-myc Chr I	This study	pSX2789
pCR8-P*dpy-7*-nCre	This study	pSX3908
pCR8-P*hyp-7*-nCre	This study	pSX3909
pCR8-P*col-182-atad-3* cDNA	This study	pSX3215
pCR8-Pcol-19-mt-NEMOs	This study	pSX3751

P*col-182*-ATAD-3(50-595)	This study	pSX3902
P*col-182*-ATAD-3(240-595)	This study	pSX3903
pcr8-P*col-19*-ATAD-3-GFP-myc	This study	pSX2845
pCR8-P*col-19*-ATAD-3(50-595)-GFP	This study	pSX3789
pCR8-P*col-19*-ATAD-3(240-595)-GFP	This study	pSX3790
pCR8-P*col-19*-ATAD-3(F47L)-GFP	This study	pSX3970
pCR8-P*col-19*-ATAD-3(G352D)-GFP	This study	pSX3891
pCR8-P*col-19*-ATAD-3(G352E)-GFP	This study	pSX3892
P*col-182*-ATAD-3(F47L)	This study	pSX3809
P*col-182*-ATAD-3(G352E)	This study	pSX3812
P*col-182*-ATAD-3(L74V)	This study	pSX3810
P*col-182*-ATAD-3(R167C)	This study	pSX3811
P*col-182*-ATAD-3(L233V)	This study	pSX3803

Supplementary Table 3. A list of primers used in this study

Primers	Gene	Sequences
zju4926	pU6-sgRNA-*atad-3* C-Ter-1 for	GCACCTGCTTGAAAATCATCGT
zju4927	pU6-sgRNA-*atad-3* C-Ter-1 rev	GATGATTTTCAAGCAGGTGCCA
zju4930	pU6-sgRNA-*atad-3* C-Ter-2 for	GTGTTGCTGAAGAAGAACGGGT
zju4931	pU6-sgRNA-*atad-3* C-Ter-2 rev	CCGTTCTTCTTCAGCAACACCA
zju5494	*atad-3*::GFP KI test for	TATGCATCTGAAACCGGAGTGC
zju5495	*atad-3*::GFP KI test rev	ACGAAAAAAAACAATAGAAAAACAAGGGCC
zju5864	syb6373 for	AACGCTACTCCGCAGATTCC
zju5865	syb6569 for	AACGTCGTCTTCGTGATATTGC
zju3673	*ttTi4348* sgRNA	GAAATCGCCGACTTGCGAGG
zju4928	pU6-sgRNA-*micu-3(C56A3.6)* sgRNA #1 for	TATGGTCGGTTGTGACATTGGT
zju4929	pU6-sgRNA- *micu-3(C56A3.6)* sgRNA #1 rev	CAATGTCACAACCGACCATACA
zju4932	pU6-sgRNA-*micu-3(C56A3.6)* sgRNA #2 for	GACAGCGAAATGATAGTCGGGT
zju4933	pU6-sgRNA- *micu-3(C56A3.6)* sgRNA #2 rev	CCGACTATCATTTCGCTGTCCA
zju4937	*micu-3(C56A3.6)* mutation for	TTAACTGCCGCAGGAATCGG

zju4938	*micu-3(C56A3.6)* mutation rev	TGTAAGAGCAACGTGGTATCTTGC
zju4971	*micu-3(C56A3.6)* mutation internal for	ATCGATAGAGATGAATTTATGCTTGTGAG

Supplementary Table 4. A list of candidate genes for the RNAi screen of regulators of mitochondrial Ca^{2+} uptake

C. elegans gene	Human homolog	*C. elegans* gene	Human homolog
atad-3	ATAD3A	*stl-1*	STOML2
C16C10.1	SLC25A40	*T20D3.5*	SLC25A53
C24D10.6	CHCHD1	*taap-1*	FAM162A
C30F12.2	AFG1L	*timm-17B.1*	TIMM17A
C56A3.6	MICU2	*timm-17B.2*	TIMM17A
chch-3	CHCHD3	*timm-23*	TIMM23
ddp-1	TIMM8B	*tin-13*	TIMM13
dnj-18	DNAJC30	*tin-44*	TIMM44
dnj-21	DNAJC15	*tin-9.2*	TIMM10B
eat-3	OPA1	*ucp-4*	SLC25A27
efhd-1	EFHD1	*ucr-1*	UQCRC1
emre-1	SMDT1	*ucr-2.1*	UQCRC2
F45G2.8	PAM16	*ucr-2.2*	UQCRC2
F52C9.3	AGK	*ucr-2.3*	UQCRC2
F54A3.5	MICOS10	*W04C9.2*	MICOS13
immp-1	IMMP1L	*wah-1*	AIFM1
immp-2	IMMP2L	*Y32H12A.7*	ADCK2
immt-1	IMMT	*Y56A3A.22*	FAM210A
immt-2	IMMT	*ymel-1*	YME1L1
K11H12.8	GHITM	*ZC477.3*	CCDC51
kin-1	PRKACA	*ZK1010.2*	RMND1
letm-1	LETM1	*micu-1*	MICU1
mcu-1	MCU	*mppb-1*	UQCRC1

moma-1	APOOL	*ncx-6*	SLC8B1

***C. elegans* gene**	**Human homolog**
ncx-9	SLC8B1
pde-2	PDE2A
phb-2	PHB2
R04F11.5	TIMM29
R07B7.10	SLC25A48
romo-1	ROMO1
scpl-4	TIMM50
sfxn-2	SFXN2
slc-25A18.1	SLC25A22
slc-25A18.2	SLC25A22
slc-25A26	SLC25A26
spg-7	AFG3L2
