# Supplementary material for: ATAD-3 couples plasma membrane injury to mitochondrial Ca2+ signaling
Source: Nat Commun. 2026 Sep 10;17:10690. doi: 10.1038/s41467-026-77690-y (PMC13655164; doi:10.1038/s41467-026-77690-y)
Supplement: Supplementary file 4 [file 41467_2026_77690_MOESM2_ESM.pdf]

Description of Additional Supplementary Files

Supplementary Movie 1. Small-scale laser wounding induces mitochondrial Ca^{2+} uptake in the *C. elegans* epidermal cell hpy7.

Supplementary Movie 2. Small-scale laser wounding cannot induce mitochondrial Ca^{2+} uptake in *mcu-1(ju1154)* mutants.

Supplementary Movie 3. Large-scale laser wounding induces mitochondrial Ca^{2+} uptake in the *C. elegans* epidermal cell.

Supplementary Movie 4. Large-scale laser wounding induces delayed mitochondrial Ca^{2+} uptake in *mcu-1(ju1154)* mutants.

Supplementary Movie 5. Large-scale laser wounding induces delayed mitochondrial Ca^{2+} uptake in *emre-1(tm6232)* mutants.

Supplementary Movie 6. Large-scale laser wounding did not induce mitochondrial Ca^{2+} uptake in *atad-3* RNAi knockdown animals.

Supplementary Movie 7. Large-scale laser wounding did not induce mitochondrial Ca^{2+} uptake in *micu-3(zju250)* mutants.
